# New Approach to the Detection of Short-Lived Radical Intermediates

**DOI:** 10.1021/jacs.2c03618

**Published:** 2022-08-24

**Authors:** Peter J. H. Williams, Graham A. Boustead, Dwayne E. Heard, Paul W. Seakins, Andrew R. Rickard, Victor Chechik

**Affiliations:** † Department of Chemistry, University of York, Heslington, York YO10 5DD, U.K.; ‡ School of Chemistry, University of Leeds, Leeds LS2 9JT, U.K.; § National Centre for Atmospheric Science, University of York, Heslington, York YO10 5DD, U.K.

## Abstract

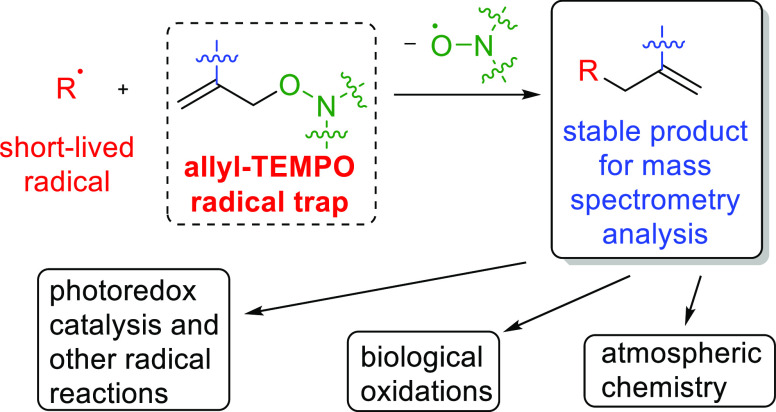

We report a new general method for trapping short-lived radicals, based on a homolytic substitution reaction S_H_2′. This departure from conventional radical trapping by addition or radical–radical cross-coupling results in high sensitivity, detailed structural information, and general applicability of the new approach. The radical traps in this method are terminal alkenes possessing a nitroxide leaving group (*e.g.*, allyl-TEMPO derivatives). The trapping process thus yields stable products which can be stored and subsequently analyzed by mass spectrometry (MS) supported by well-established techniques such as isotope exchange, tandem MS, and high-performance liquid chromatography-MS. The new method was applied to a range of model radical reactions in both liquid and gas phases including a photoredox-catalyzed thiol–ene reaction and alkene ozonolysis. An unprecedented range of radical intermediates was observed in complex reaction mixtures, offering new mechanistic insights. Gas-phase radicals can be detected at concentrations relevant to atmospheric chemistry.

## Introduction

Short-lived radical intermediates play a key role in many chemical processes, including synthetic chemistry (*e.g.*, polymerization^1^ and photoredox catalysis^2^), biochemistry (*e.g.*, oxidative stress^3^), and atmospheric chemistry (*e.g.*, photochemical oxidation cycles^4^ and secondary organic aerosol formation^5^). However, their detection is challenging due to their short lifetimes and hence low concentrations in real systems, which are often below the detection thresholds of conventional analytical techniques. In addition, one may want to detect radicals in environments where deployment of complex instrumentation can be difficult, for example, in atmospheric field measurements.

Electron paramagnetic resonance (EPR) spectroscopy detects radicals directly, but this can be very challenging for short-lived radicals, and gaseous radicals can only usually be observed at reduced pressure.^6^ In the gas phase, many radicals can also be directly detected using mass spectrometry (MS) techniques (*e.g.*, chemical ionization MS, CI-MS, or vacuum ultraviolet photoionization MS, VUV-PIMS) or laser spectroscopy (*e.g.*, laser-induced fluorescence, LIF). VUV-PIMS, in particular, can detect any radical and distinguish between isomers.^7^ High resolution and high sensitivity, required for analysis of complex systems, can be achieved with synchrotron VUV-PIMS. Direct spectroscopic radical detection, however, requires advanced instrumentation which may not be suitable for all scenarios such as field or atmospheric chamber work.

More commonly used methods with a broad application scope therefore usually detect radicals indirectly, following their conversion (*e.g.*, by trapping) to longer-lived species. For example, liquid-phase ^•^OH can be detected by UV–vis or fluorescence spectroscopy after addition to aromatic scavengers.^8^ In the gas phase, ROxLIF^9^ and peroxy radical chemical amplification (PERCA)^10^ are indirect techniques that predominantly measure total RO_2_^•^ following their chemical conversion to other species.

**Figure 1:**
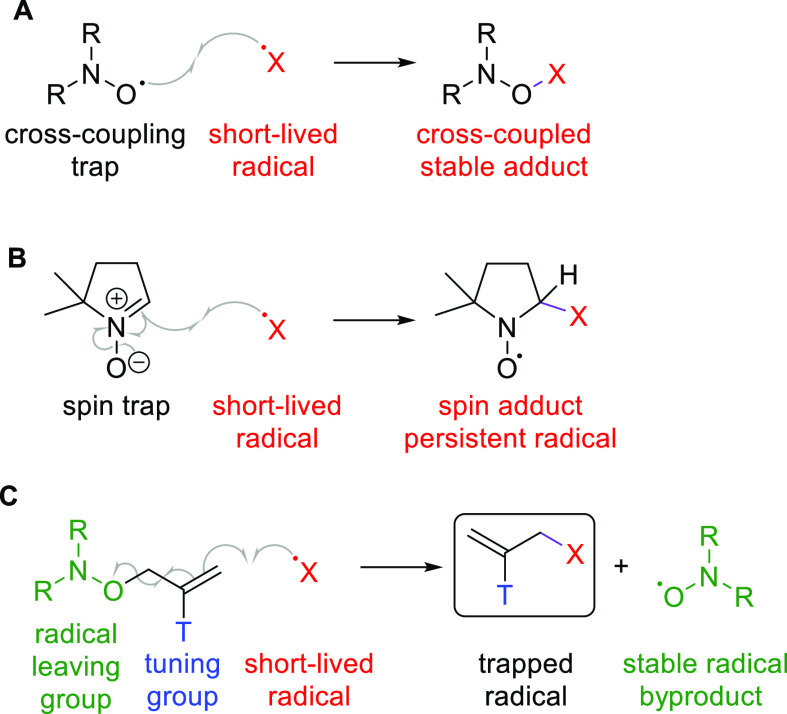
Radical traps. (A) Cross-coupling trapping. (B) Spin trapping. (C) Novel radical trap design and the S_H_2′ trapping mechanism.

A popular method for trapping carbon-centered radicals in the liquid phase is *via* cross-coupling with persistent radicals such as nitroxides (Figure 1A). The alkoxyamine adducts formed are then studied using common characterization techniques, including MS.^11−15^ This method is often applied to liquid-phase radicals, for example, in homogeneous catalysis. MS characterization is highly sensitive and provides structural information. However, nitroxide trapping is rarely applicable to heteroatom-centered radicals, which significantly limits its scope. In addition, the high reactivity of nitroxides makes them non-innocent components of many reaction mixtures.

Arguably the most common method of radical trapping, applicable to most short-lived radicals in both liquid and gas phases, is the spin trapping technique. This typically relies on fast and selective radical addition to the double bond in nitrone or nitroso traps, yielding persistent nitroxide radicals that accumulate to concentrations detectable by EPR spectroscopy (Figure 1B).^16^ Spin trapping has been widely used to study a variety of radical reactions using both EPR and MS detection.^17−20^ Unfortunately, this method has many well-documented drawbacks, including false positives caused by side reactions, limited structural information of the trapped radical, poor sensitivity, and often short lifetimes of radical adducts ranging from seconds to hours.^21−24^

Apart from addition and cross-coupling, radicals can also be trapped *via* a substitution reaction. For instance, ^•^Cl was trapped by aromatic ipso-substitution of a nitro group in 1,1-diphenyl-2-picrylhydrazyl (DPPH).^25^ However, the yields of ipso-substitution are often poor, and many radicals do not undergo this reaction,^26^ significantly limiting the scope of this approach.

Here, we report a new class of radical traps, which overcome many of the aforementioned drawbacks and enable facile detection of most short-lived radicals. Radical trapping proceeds *via* a homolytic substitution reaction S_H_2′. The key design feature is the presence of a good radical leaving group (a nitroxide) at the allylic position of a terminal alkene. Reaction of a short-lived radical with the trap releases the nitroxide radical and yields a stable, non-radical product (Figure 1C). The thermodynamic driving force for this reaction is the weakness of the C-ONR_2_ bond (typically <170 kJ/mol).^27^ Our design also incorporates a functional “tuning” group “T” that can be varied to optimize the chemical (*e.g.*, rate of radical addition) and physical (*e.g.*, solubility, MS ionization efficiency) properties of the traps. The concentration of released nitroxides is low compared to cross-coupling trapping (Figure 1A) as only one equivalent is released per trapped radical.

This paper aims to demonstrate the broad scope of applications of the new traps. The new method was used to trap radical intermediates in a range of systems, from relatively simple liquid- and gas-phase radical reactions to more complex processes such as terpene ozonolysis.

## Results and Discussion

### Synthesis of the New Traps

**Figure 2:**
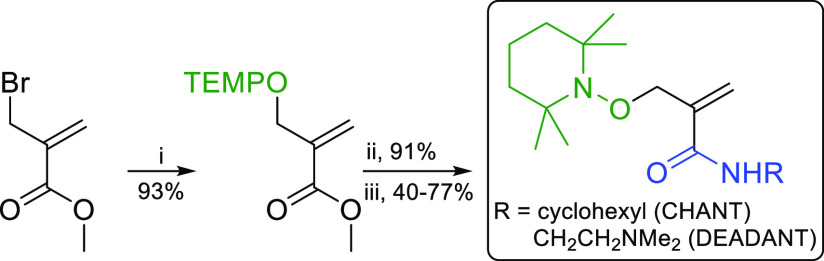
Synthetic procedure for amide-functionalized traps. TEMPO = (2,2,6,6-tetramethylpiperidin-1-yl)oxyl. (i) TEMPO, NaI, Na_2_SO_3_, MeCN, N_2_, 65 °C, 48 h. (ii) NaOH/1,4-dioxane, 24 h. (iii) H_2_NR, HBTU, DIPEA, DMF, 18 h.

Two allyl-(2,2,6,6-tetramethylpiperidin-1-yl)oxyl (TEMPO)-based traps containing alkyl (CHANT) or tertiary amine (DEADANT) functional groups were prepared from commercially available starting materials in three steps with acceptable yields (34–65% overall, Figure 2 and Supporting Information Sections S1–S3, S9.1). The conjugated amide group increased the reactivity of the traps, particularly toward electron-rich radicals. DEADANT yielded trapped products that could be ionized more efficiently, improving MS sensitivity. The new traps (particularly CHANT) showed good chemical stability in the presence of many non-radical reactive species (Supporting Information Section S4) and had a shelf life of at least 3 months when stored neat at room temperature. We note that no false positives (*i.e.*, compounds with the same structure as that of the trapped radicals but formed *via* a non-radical pathway) have been detected in reactions reported herein, which constitutes a marked improvement over conventional spin trapping.^21,23,24^

In order to detect short-lived radical intermediates, the new allyl-TEMPO-based traps are added to the reaction of interest, similarly as for radical–radical cross-coupling and spin trapping. The trapped radicals are then analyzed by electrospray ionization (ESI) MS (Supporting Information Section S6). As most trapped radicals are bench-stable, trapping can be performed without complex equipment (*e.g.*, for field measurements), and the products can be analyzed at a later date. This is an important advantage compared to conventional spin trapping where most radical adducts have limited lifetime (typically ranging from seconds to hours). Trapped radicals are detected as MS peaks with the *m*/*z* value corresponding to the mass of the radical plus that of the CHANT or DEADANT fragment (166.1232 or 155.1184, respectively) plus H^+^ (1.0078) or Na^+^ (22.9898).

### Radical Detection with Allyl-TEMPO-Based Traps in Liquid-Phase Reactions

**Figure 3:**
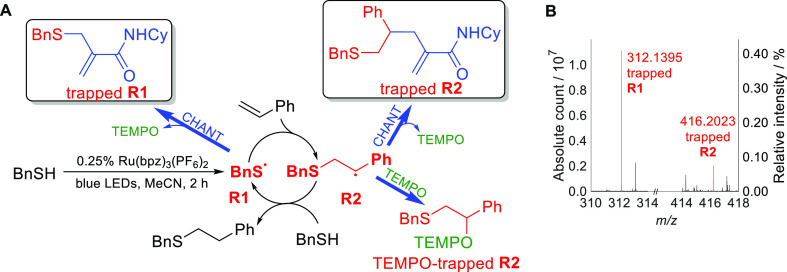
Trapping intermediate radicals in the photoinitiated thiol–ene addition using CHANT. Bn = CH_2_Ph, Cy = cyclohexyl. (A) Reaction mechanism and structures of trapped **R1** and **R2**. (B) Background-corrected mass spectrum of the thiol–ene trapping reaction, showing peaks corresponding to trapped radicals **R1** and **R2**. Intensity is relative to the CHANT standard (at the same concentration as that used in the reaction).

The feasibility of the novel radical-trapping method was first probed by applying it to a model radical reaction in the liquid phase, a Ru-catalyzed photoinitiated (blue light-emitting diode, LED) thiol–ene addition (Supporting Information Sections S5.1, S5.2).^28,29^ This reaction proceeds via a well-understood radical chain mechanism, with radical addition and hydrogen atom abstraction propagation steps (Figure 3A).^30^ CHANT was used as a radical trap due to its robustness. It also does not absorb light in the blue LED spectral output range (Supporting Information Section S4.4).

MS peaks corresponding to both trapped radical intermediates **R1** (BnS^•^) and **R2** (BnSCH_2_CH^•^Ph) were observed in the reaction mixtures (Supporting Information Sections S8.1, S8.2). Despite the large number of other products and non-radical intermediates, these peaks were clearly visible in the mass spectrum (Figure 3B). In conventional spin trapping, EPR spectra only show signals for trapped radicals, as all other compounds are usually EPR-silent, even in complex mixtures. MS spectra, however, show peaks for all constituents. This complexity is advantageous, as non-radical intermediates and products can be detected simultaneously with the trapped radicals. We note that unambiguous determination of elemental composition requires high-resolution MS instruments (*e.g.*, a standard time-of-flight mass spectrometer with a 10^4^ mass resolving power is sufficient for most systems), which are widely available to most research laboratories. Just like with any MS experiment, the elemental composition, supported by the knowledge of the likely constituents of the reaction mixture, makes it possible to assign the structures of the trapped radicals with a high degree of certainty.

Simultaneous detection of **R1** and **R2** made it possible to compare their relative concentrations. We note that concentrations of any short-lived radical intermediate depend on the rate of their formation and the rate of their decay. The concentration of the trapped species is additionally dependent on the rate of trapping (and the rate of decay of the trapped radicals if they are unstable, *e.g.*, in conventional spin trapping). This is true for any trapping methodology. Fortunately, the rates of radical addition to alkenes are well-studied, and the availability of kinetic data helps relate MS peak intensities of trapped radicals to the concentrations of the original radical intermediates.

MS peaks corresponding to trapped **R1** had similar but somewhat greater intensity compared to that of **R2** (Figure 3B). However, the rate of trapping of thiyl radical **R1** by CHANT is estimated to be at least 1000 times greater than that of carbon-centered **R2**.^31,32^ Assuming a similar ionization efficiency of the trapped **R1** and **R2** species, this would suggest that **R2** is the resting state for this radical chain process, which is consistent with the literature rate constants for related reactions (Supporting Information Section S8.2.2).

A more accurate quantitative comparison of MS peak intensities could not be made as ionization efficiency may depend on the composition of the analyte. We note that synthesis of an isotopically labeled trap (*e.g.*, by using perdeuterated cyclohexylamine in the CHANT synthesis) and independent synthesis of labeled trapped radicals (or their isolation from reaction mixtures) would allow for accurate quantification using isotope dilution analysis.

**R2** was also trapped with the TEMPO radical released during CHANT trapping (Figure 3A, Supporting Information Section S8.2.1). This TEMPO-trapped **R2** had much greater intensity than the CHANT-trapped **R2**, likely due to the faster trapping rate and better ionization efficiency of the alkoxyamine compound. However, TEMPO trapping is limited to carbon-centered radicals: no MS peak corresponding to TEMPO-trapped **R1** was observed. The ability of the new traps to simultaneously detect carbon- and heteroatom-centered radicals such as **R1** constitutes a significant advantage over TEMPO trapping. Successful radical capture with the new traps was further unambiguously confirmed by the isolation of the pure product of PhS^•^ trapping with CHANT from a reaction mixture optimized for its formation from PhSH, in a 63% yield (Supporting Information Sections S5.2.2, S9.2.1).

**Figure 4:**
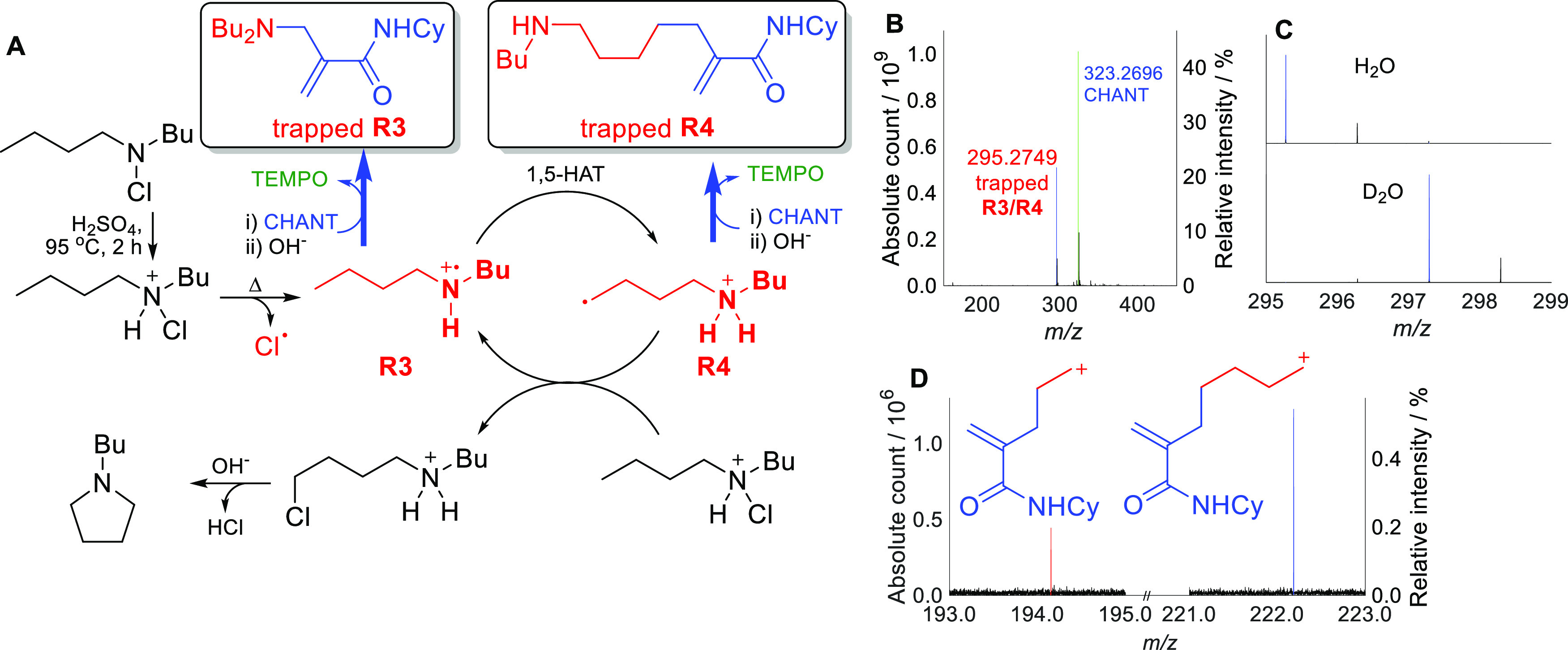
Radical trapping in the HLF reaction using CHANT. (A) Reaction mechanism and structures of trapped **R3** and **R4**. (B) Background-corrected mass spectrum of the HLF trapping reaction, showing peaks corresponding to unreacted CHANT and trapped radicals **R3**/**R4**. Intensity is relative to the CHANT peak before the reaction. (C) Background-corrected mass spectra of the HLF trapping reaction in H_2_O and D_2_O, indicating trapped **R3**. (D) Tandem mass spectrum of the *m*/*z* 295 peak (Figure 4B) showing two peaks which could only be attributed to fragments of trapped **R4**. Intensity is relative to the parent ion.

S_H_2′-based radical trapping was next applied to the Hofmann–Löffler–Freytag (HLF) reaction, a cyclization of N-halogenated amines which is believed to proceed by homolysis of the N-halogen bond (Figure 4A, Supporting Information Sections S5.3, S8.3, S9.2.2).^33,34^ MS peaks corresponding to trapped nitrogen- and/or carbon-centered radicals **R3** and/or **R4** were clearly visible in the mass spectrum at *m*/*z* 295.2749 (Figure 4B). Although these two species have identical molecular formulae, they could be distinguished using D_2_O exchange experiments, as trapped **R4** has three exchangeable protons in the protonated ion (two on the ammonium and one in the CHANT residue), whereas trapped **R3** has two. The shift of the *m*/*z* 295 peak by 2 mass units upon D_2_O exchange was thus consistent with the structure of the trapped **R3** (Figure 4C). The peak corresponding to trapped **R4** was not observed. However, tandem MS of the *m*/*z* 295 peak showed two low-intensity peaks that could only be attributed to trapped **R4** (Figure 4D). Most other (strong) tandem MS peaks could be attributed to either species. We conclude that the new traps enabled detection of not only the dominant **R3** but also a small amount of **R4**. This is consistent with the literature evidence that the 1,5-hydrogen atom transfer (1,5-HAT) is rate-determining^35,36^ and confirms trapping of the nitrogen-centered radical **R3**.^37,38^ In addition, observation of strong MS peaks of CHANT-trapped radicals suggests that they are stable in mild acids and at high temperature (95 °C).

The above-mentioned results demonstrated the potential of the new method for the detection of radicals in relatively simple liquid-phase reactions. We have also applied it to a range of other reactions including aqueous ^•^OH-initiated degradation of alcohols (Supporting Information Sections S5.9, S8.9), nucleotides (Supporting Information Sections S5.10, S8.10), saccharides (Supporting Information Sections S5.10, S8.11), and antioxidants (Supporting Information Sections S5.10, S8.12); synthetically useful Barton (Supporting Information Sections S5.6, S8.6) and Hunsdiecker reactions (Supporting Information Sections S5.7, S8.7); and decarboxylative iodination (Supporting Information Sections S5.8, S8.8). In all reactions, which included complex mixtures, we detected a range of radical intermediates. New mechanistic information was obtained. For instance, the latter reaction (iodination of aromatic carboxylic acids) was previously suggested to proceed *via* a concerted decarboxylation/iodination of the intermediate hypoiodite.^39^ However, the observation of a strong MS peak for the trapped carboxylate radical (>4% intensity relative to the trap before initiation) points to the viability of an alternative radical pathway (Supporting Information Section S8.8).

### Radical Detection with Allyl-TEMPO-Based Traps in Complex Gas-Phase Reactions

**Figure 5:**
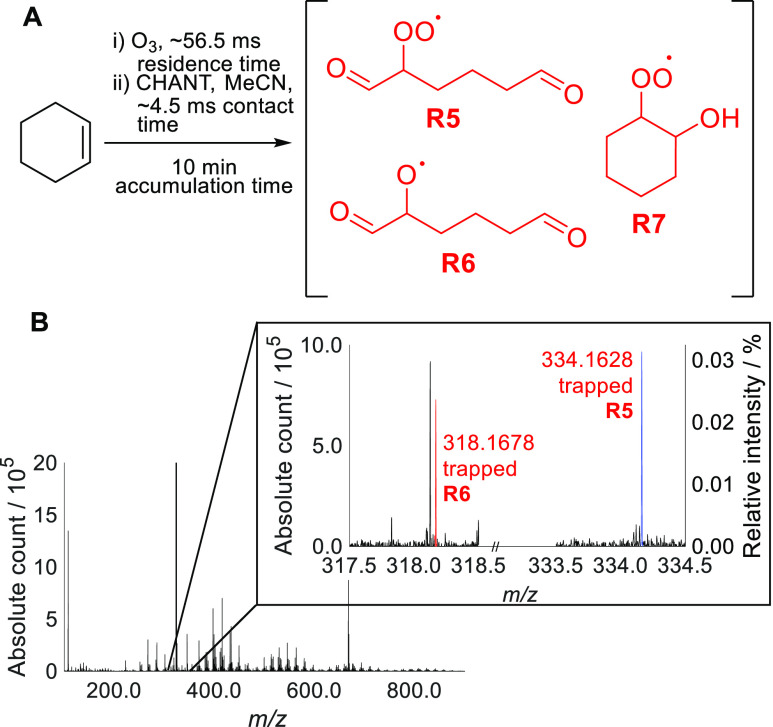
(A) Selected radical intermediates in cyclohexene ozonolysis. (B) Background-corrected mass spectrum of cyclohexene ozonolysis with CHANT trapping, showing trapped **R5** and **R6**. Intensity is relative to the CHANT peak before the reaction.

We next investigated gas-phase alkene ozonolysis, which is relevant to atmospheric chemistry.^40^ This reaction proceeds through the formation of an unstable ozonide that fragments into a primary carbonyl and an excited Criegee intermediate.^41^ The latter typically decomposes through several steps to form a complex array of radical intermediates such as ^•^OH, HO_2_^•^, peroxyl (RO_2_^•^), and alkoxyl (RO^•^) radicals and a large number of oxidized products including highly oxidized multifunctional (HOM) compounds.^5,42,43^ A model cyclohexene ozonolysis system was initially investigated (Figure 5A). O_3_ was generated by UV photolysis of O_2_ (Supporting Information Section S5.4), followed by mixing with a gaseous alkene. The gas stream was then allowed to react for a set residence time (typically 56.5 ms) before being bubbled through a solution of CHANT. We estimate the contact time of the gas bubbles with the trapping solution as *ca.* 4.5 ms. The bubbling continued for a set accumulation time (typically 10 min). CHANT showed negligible reaction with ozone under the reaction conditions (Supporting Information Section S8.4.1).

**Figure 6:**
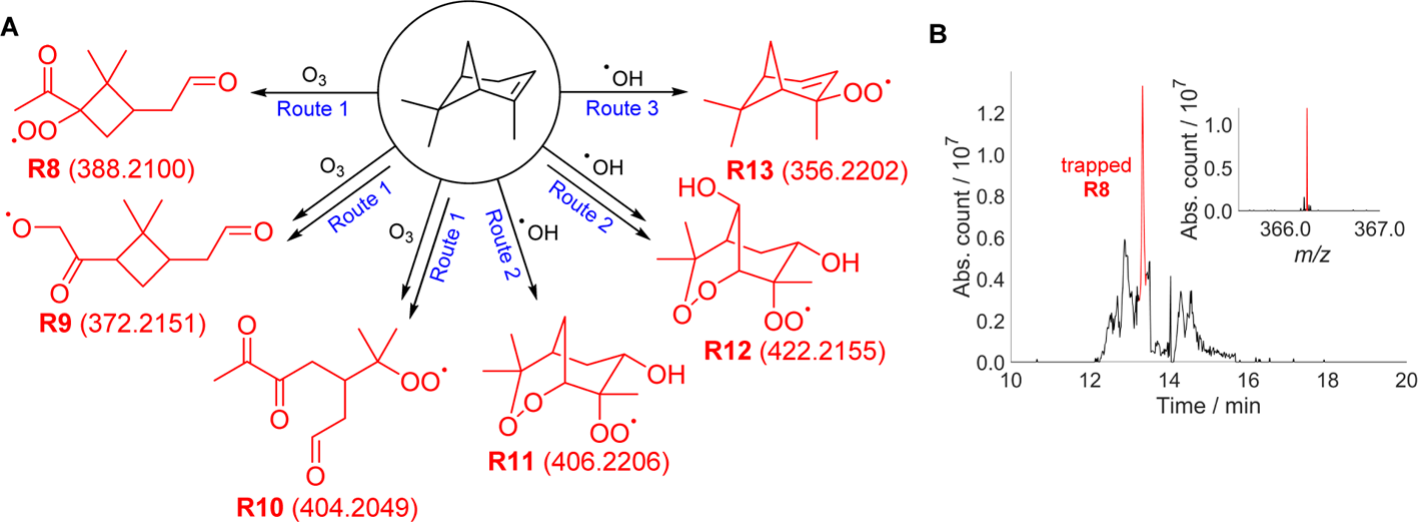
(A) Hypothesized structures of detected radicals (predicted *m*/*z* values of sodiated CHANT adducts) in Table 1. (B) HPLC-MS chromatogram and the mass spectrum (inset) of the *m*/*z* 366.228 peak corresponding to trapped **R8** detected in the α-pinene ozonolysis gas stream bubbled through CHANT solution. The MS source was sent to waste between 13.5–14.0 min, to remove unreacted CHANT. The mass spectrum of trapped **R8** (observed as a protonated ion as these gave the strongest peaks in HPLC-MS experiments) is at time of the maximum intensity. Other structural isomers were also predicted for these molecular formulae (Supporting Information Section S8.4.3.1).

In relatively simple reactions (*e.g.*, synthetic liquid-phase radical chemistry), the signals of trapped radicals could be among the strongest peaks in the spectra (*e.g.*, Figure S15). Ozonolysis reactions, on the other hand, are much more complex, where MS spectra were heavily dominated by a wide range of products and non-radical intermediates (Figure 5B). Despite the presence of a large number of other species, MS peaks corresponding to trapped alkoxyl and peroxyl radicals **R5**, **R6**, and **R7** were clearly visible in the mass spectrum of the reaction mixture (Figure 5B and Supporting Information Section S8.4.2). Encouraged by these results, we used CHANT to detect radical intermediates formed during α-pinene ozonolysis (Supporting Information Sections S5.4, S8.4.3). Atmospheric ozonolysis of this biogenic monoterpene is an important non-photolytic contributor to the formation of ^•^OH and other radicals and secondary organic aerosols (SOAs).^40,42,44,45^ Owing to the complexity of this system, MS analysis involved automated prediction of molecular formulae corresponding to observed *m*/*z* peaks. These molecular formulae were then assigned to products and trapped radicals. Molecular formula limits were set to only identify monomeric non-fragmented CHANT-trapped radicals (Table 1). Using the molecular formulae thus obtained, radical structures for all but 3 out of the 10 most intensely observed species were identified (Figure 6A, Supporting Information Section S8.4.3.2).

**Table 1:** Identified Radicals from the Ten Most Intense MS Peaks Attributed to Monomeric Non-Fragmented Trapped Radicals from α-Pinene Ozonolysis CHANT Trapping (Figure 6A)^a^

route	observed *m*/*z*	relative intensity/%	corresponding radical molecular formula^b^	example identified structure
3	356.2198	0.097	C_10_H_15_O_2_^•^	**R13**
2	422.2153	0.070	C_10_H_17_O_6_^•^	**R12**
2	406.2196	0.063	C_10_H_17_O_5_^•^	**R11**
1	388.2097	0.035	C_10_H_15_O_4_^•^	**R8**
3	334.2378	0.024	C_10_H_15_O_2_^•^^c^	**R13**
1	372.2147	0.020	C_10_H_15_O_3_^•^	**R9**
1	404.2049	0.014	C_10_H_15_O_5_^•^	**R10**

a Molecular formula limits were set as C_20_H_0–38_N_1_O_1–10_Na_0–1_ and *m*/*z* limits as 100–500. Unreasonable molecular formulae were eliminated. Intensity is quoted relative to the MS intensity of a trap standard.

b Observed as a sodiated adduct with the CHANT residue unless stated otherwise.

c Observed as a protonated adduct.

Three molecular formulae were attributed to radicals formed following ozone addition across the α-pinene double bond (**R8**, **R9**, and **R10**, route 1).^46^ Two molecular formulae were attributed to radicals formed following ^•^OH addition to the α-pinene double bond (**R11** and **R12**, route 2). β-Hydroxyperoxyl radicals analogous to **R7** were not observed, despite the literature indicating that this pathway constitutes ∼45–70% ^•^OH reactivity with α-pinene.^46,47^ A final molecular formula was attributed to radicals formed following ^•^OH-initiated H-atom abstraction (**R13**, route 3). The literature indicated that this pathway constitutes ∼10% ^•^OH reactivity with α-pinene, despite its corresponding CHANT-trapped radical being observed with the greatest intensity.^46−48^ The structural similarity of the trapped peroxyl radicals suggests that they have similar ionization efficiencies. The trapping rates of all peroxyl radicals are likely to be comparable, and their rates of decay should be similar. Therefore, the relatively high intensity of trapped **R13** suggests that ^•^OH-initiated abstraction of an allylic H-atom may play a more significant role in α-pinene ozonolysis than previously thought. The new traps thus enabled simultaneous detection of many radical intermediates and products in a very complex gaseous reaction mixture.

Elemental compositions obtained from high-resolution MS spectra do not distinguish between isomers. A range of further MS techniques are available to make structure assignment more certain, and they were used here to validate the hypothesized structures of trapped radicals (Figure 6A). The ratio between monoisotopic MS peaks and their first ^13^C satellites was used to estimate the number of carbon atoms in species corresponding to these peaks (Supporting Information Section S8.4.3.3). Fragmentation peaks observed using tandem MS aided structure elucidation of parent MS peaks (Supporting Information Section S8.3.3). D_2_O exchange studies were used to determine the number of labile protons associated with each peak which made it possible to differentiate some structural isomers. In particular, the structure of trapped **R13** was confirmed as a peroxyl radical rather than an isomeric ω-hydroxylated alkoxyl radical which would have contained an extra labile proton (Supporting Information Section S8.4.3.5). High-performance liquid chromatography (HPLC)-MS was used to significantly clean mass spectra, improve MS peak detection, and indicate the number of species for each MS peak. In relatively simple liquid- and gas-phase reactions, the number of HPLC peaks matches the number of expected isomers (Supporting Information Figures S30, S31, S36). In highly complex systems (*e.g.*, α-pinene ozonolysis), the chromatograms could show several peaks of isomers contributing to the same *m*/*z.* In these cases, the structure assignment is less certain, but it could be strengthened by additional information. For example, the chromatogram of the *m*/*z* 366.228 peak showed several peaks (Figure 6B). This *m*/*z* value is consistent with the trapped **R8** (as a protonated ion), predicted to be a major intermediate in the Master Chemical Mechanism (MCM).^46,49−51^ The corresponding sodiated ion (*m*/*z* 388.2097) observed with direct-injection MS (without HPLC) is one of the strongest trapped radical peaks in the spectrum (Table 1), confirming the significant role of this radical in the reaction.

### Sensitivity of the New Method

**Figure 7:**
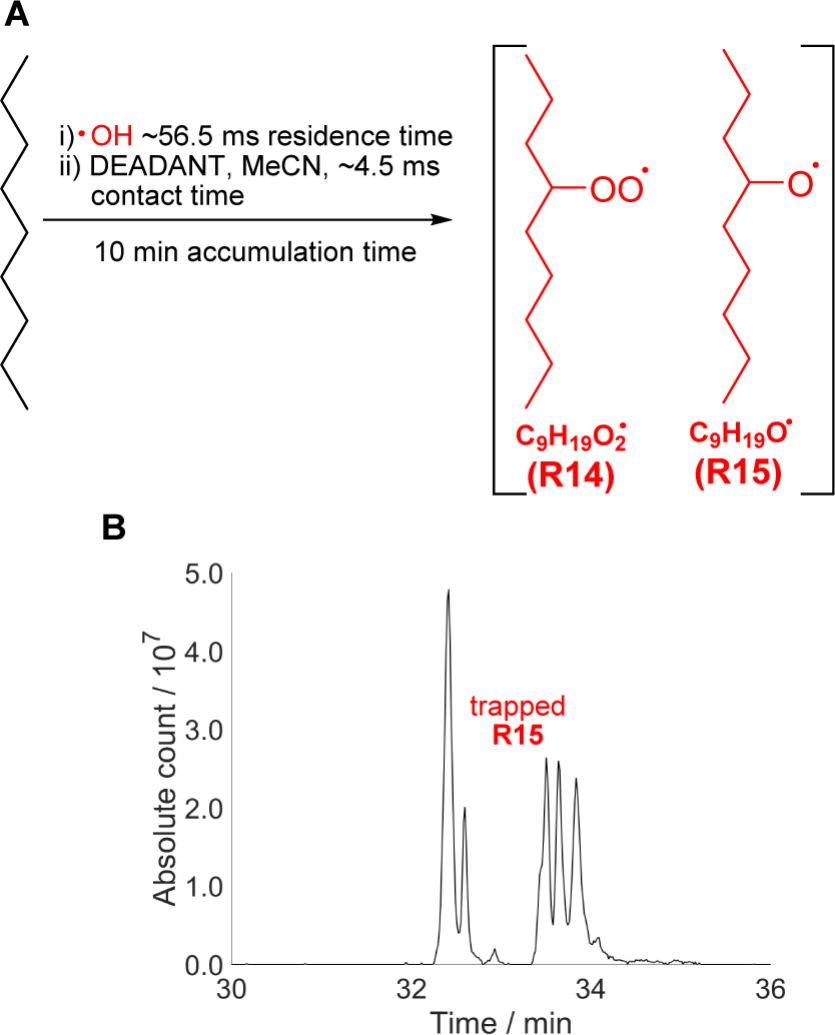
(A) Selected radical intermediates in ^•^OH-initiated autoxidation of *n*-nonane. (B) HPLC-MS chromatogram of the peak corresponding to CHANT-trapped RO^•^ (*m*/*z* 299.270, detected in ^•^OH-initiated *n*-nonane degradation). The five distinct peaks observed are believed to correspond to the five possible RO^•^ structural isomers.

Finally, we set out to determine the minimum concentration of gaseous radicals which the new traps could detect, in a model alkane oxidation system, *n*-nonane + ^•^OH (Figure 7A). ^•^OH was generated by water photolysis and calibrated using laser-induced fluorescence (LIF) detection at low pressure, known as fluorescence assay by gas expansion (FAGE), as described by Onel *et al.*(52) (Supporting Information Sections S5.5, S8.5). Since this system generated minimal ozone, DEADANT (which is degraded by ozone but gives higher MS ionization efficiencies of trapped radicals) was used as the trap. MCM modeling (Supporting Information Sections S8.5, S7.1, S10) was employed to calculate gaseous [C_9_H_19_O_2_^•^] (**R14**) as 1.7 × 10^11^ molec cm^–3^ and [C_9_H_19_O^•^] (**R15**) as 2.1 × 10^3^ molec cm^–3^. Unfortunately, MS peaks corresponding to trapped **R14** could not be detected after a 10 min accumulation time. RO_2_^•^ radicals such as **R14** are trapped much slower than most other radical intermediates (*e.g.*, they undergo addition to double bonds at least 10^8^ times slower than RO^•^ radicals like **R15**, Supporting Information Section S7.2). Therefore, RO_2_^•^ accumulated in the trapping solution, and their reaction with the trap was outcompeted by other reactions in the liquid phase, including self-reaction (Supporting Information Section S7.2). The sensitivity of detection of trapped RO_2_^•^ was further reduced by their partial degradation in the HPLC column (Supporting Information Section S7.2). Reduction of peroxides commonly used in sample preparation for MS analysis would not have been helpful here as it would have made it impossible to distinguish between trapped RO^•^ and RO_2_^•^.

One product of the RO_2_^•^ (*e.g.*, **R14**) self-reaction is the corresponding RO^•^ (*e.g.*, **R15**). In fact, modeling showed that almost all **R15** in the trapping solution was produced by the **R14** self-reaction rather than by absorption from the gas phase (Supporting Information Section S7.2). Trapped **R15** can thus be used as a proxy for **R14**. The HPLC-MS chromatogram of the *m*/*z* 299.270 peak (matching trapped **R15**) showed five distinct peaks (Figure 7B). As there are five possible **R15** isomers and no other reasonable compounds have the same elemental composition, the HPLC-MS peaks were attributed to the five isomers of trapped **R15**. The new traps can thus be used to indirectly detect gaseous RO_2_^•^ radicals (*via* trapped RO^•^) with the detection threshold estimated as *ca.* 1.5 × 10^9^ molec cm^–3^ (Supporting Information Section S7.2) which is comparable to the peak RO_2_^•^ concentrations observed in polluted urban environments. These results confirm the high sensitivity of the radical-trapping method and highlight the importance of (self-)reactions of less-reactive gaseous radicals, such as RO_2_^•^, following their accumulation in the trapping solution. We note that issues associated with the slow rate of RO_2_^•^ trapping are equally important in other trapping methods including conventional spin trapping.

## Conclusions

Although direct detection of free radical intermediates has many advantages, there are occasions when it is impractical. For instance, radical concentrations in real systems are often too low for direct EPR detection. Equipment availability and the requirement to sample reaction mixtures directly into the instrument limit the scope of MS-based techniques such as VUV-PIMS. In these cases, radical trapping becomes a method of choice as it allows one to accumulate products and provides temporal separation of sampling from analysis.

We have developed a new class of radical traps (allyl-TEMPO derivatives), which enable conversion of most short-lived radical intermediates into stable products. Coupled with MS analysis, this radical trapping approach combines the best features of the two most common alternatives: spin trapping with EPR detection (applicability to most short-lived radicals) and TEMPO cross-coupling with MS detection (high sensitivity, detailed structural information).

The new traps can be applied to both gas- and liquid-phase reactions. Simultaneous detection of trapped radicals, intermediates, and (by)products in the same reaction mixture makes this method an excellent mechanistic tool for studying radical reactions in highly complex systems. Just like with any other trapping technique, kinetics of the trapping reaction needs to be considered, and for some relatively longer-lived radicals (*e.g.*, RO_2_^•^), the trapping reaction can be outcompeted by other reactions such as the self-reaction. Nonetheless, the excellent sensitivity of MS makes the new method suitable for trapping radical intermediates in a diverse range of complex systems, including reactions relevant to atmospheric chemistry.

An important feature of the allyl-TEMPO-based traps is that trapped radicals are highly unlikely to be formed *via* non-radical pathways, thus reducing the probability of artefacts. Although allyl-TEMPO-based traps can undergo slow nucleophilic addition with strong nucleophiles, the resulting adducts do not have the same structure as that of the products of radical trapping and hence do not lead to false positives commonly seen in conventional spin trapping.

## Supporting Information Available

Experimental data including synthesis details, radical trapping procedures and mass spectrometry protocols, additional data, and spectra (PDF)

## Notes

The data supporting this research is openly available from the University of York data repository at https://doi.org/10.15124/907fd184-1a00-4aff-ab43-315392e42c46.
