# Supplementary material for: New Approach to the Detection of Short-Lived Radical Intermediates
Source: J Am Chem Soc. 2022 Aug 24;144(35):15969–76. doi: 10.1021/jacs.2c03618 (PMC9460783; doi:10.1021/jacs.2c03618)
Supplement: Supplementary file 1 — ja2c03618_si_001.pdf [file ja2c03618_si_001.pdf]

Supplementary Information

A New Approach to the Detection of Short-Lived Radical Intermediates

Peter J. H. Williams[1], Graham A. Boustead[2], Dwayne E. Heard[2], Paul W. Seakins[2], Andrew R. Rickard[1,3]*, Victor Chechik[1]*

[1]Department of Chemistry, University of York, Heslington, York YO10 5DD, UK

[2]School of Chemistry, University of Leeds, Leeds LS2 9JT, UK

[3]National Centre for Atmospheric Science, University of York, Heslington, York YO10 5DD, UK

*Corresponding author. Email: victor.chechik@york.ac.uk; andrew.rickard@york.ac.uk

Contents

S1. General information 4
S2. Chemicals 5
S3. Trap synthesis 6
S3.1. Methyl 2-(TEMPOmethyl)acrylate 6
S3.2. 2−(TEMPOmethyl)acrylic acid 6
S3.3. Standard amide coupling reaction procedure 7
S3.4. CHANT 7
S3.5. DEADANT 8
S4. Trap properties and stability studies 10
S4.1. Free TEMPO concentration 10
S4.2. Oxidation and reduction 10
S4.3. Michael addition 11
S4.4. Absorbance of blue LED light (455 nm) 13
S4.5. MS calibration curves 14
S5. Radical trapping procedures 17
S5.1. General 17
S5.2. Thiol-ene addition 17
S5.2.1. Trapping reaction 17
S5.2.2. Isolation of CHANT-trapped PhS• (*N*-cyclohexyl-2-[(phenylsulfanyl)methyl]acrylamide) 17
S5.3. Hofmann-Löffler-Freytag (HLF) reaction 18
S5.3.1. Precursor synthesis 18
S5.3.2. Trapping reaction 19
S5.4. Alkene ozonolysis 19
S5.5. Gaseous •OH-initiated *n*-nonane degradation 20
S5.6. Barton reaction 21

S5.7. Hunsdiecker reaction 21
S5.7.1. Precursor synthesis 21
S5.7.2. Trapping reaction 22
S5.8. Decarboxylate aromatic iodination reaction 22
S5.9. Aqueous •OH-initiated degradation of methanol 22
S5.10. Aqueous •OH-initiated degradation of thymine, glucose and ascorbic acid 23
S6. MS procedures for trapping reaction analysis 24
S6.1. Characterisation 24
S6.2. Analysis 24
S6.3. Parameters 25
S7. Kinetic modelling of nonane oxidation 27
S7.1. Gas phase modelling 27
S7.2. Modelling reactions in the trapping solution 27
S8. Additional trapping reaction schemes and results 29
S8.1. General 29
S8.2. Thiol-ene addition 30
S8.2.1. MS results 30
S8.2.2. Analysis of relative steady-state concentrations of R1 and R2 31
S8.3. Hofmann-Löffler Freytag (HLF) reaction 32
S8.3.1. MS results 32
S8.3.2. D_2O exchange 33
S8.3.3. Tandem MS 34
S8.4. Alkene ozonolysis 37
S8.4.1. CHANT ozonolysis 37
S8.4.2. Cyclohexene ozonolysis 38
S8.4.3. α-Pinene ozonolysis 40
S8.4.3.1. Reaction schemes 40
S8.4.3.2. MS data 45
S8.4.3.3. Monoisotopic peak to ^{13}C satellite ratio 47
S8.4.3.4. Tandem MS 48
S8.4.3.5. D_2O exchange 48
S8.5. Gaseous •OH-initiated *n*-nonane degradation 49
S8.6. Barton reaction 51
S8.7. Hunsdiecker reaction 52
S8.8. Decarboxylate aromatic iodination reaction 52
S8.9. Aqueous •OH-initiated degradation of methanol 54
S8.10. Aqueous •OH-initiated degradation of thymine 54

S8.11. Aqueous •OH-initiated degradation of glucose ... 56
S8.12. Aqueous •OH-initiated degradation of ascorbic acid ... 59
S9. Spectra ... 61
S9.1. Trap synthesis ... 61
S9.1.1. Methyl 2-(TEMPOmethyl)acrylate ... 61
S9.1.2. 2−(TEMPOmethyl)acrylic acid ... 63
S9.1.3. CHANT ... 66
S9.1.4. DEADANT ... 68
S9.2. Trapping reactions ... 70
S9.2.1. Thiol-ene addition: Isolation of CHANT-trapped PhS• (*N*-cyclohexyl-2-[(phenylsulfanyl)methyl]acrylamide) ... 70
S9.2.2. Hofmann-Löffler-Freytag (HLF) reaction: characterisation of precursor ... 72
S10. Kinetic modelling equations ... 73
S11. References ... 74

S1. General information

Except where stated, all reagents and solvents were purchased from commercial sources and used without further purification. Anhydrous solvents were obtained from an Innovative Technology Inc. PureSolv® solvent purification system.

All chemical reactions and analyses were acquired at room temperature (293 K) and pressure unless stated otherwise. Thin layer chromatography was carried out on Merck silica gel 60F254 pre-coated aluminium foil sheets and were visualised using a basic aqueous potassium permanganate stain, as indicated. Flash column chromatography was carried out using slurry packed Fluka silica gel (SiO_2), 35–70 µm, 60 Å, eluting with the specified solvent system. Melting points were determined using Stuart Scientific SMP3 apparatus and are uncorrected. CHN microanalysis was obtained using an Exeter Analytical Inc. CE-440 analyser. Infrared (IR) spectra were recorded on a PerkinElmer UATR 2 spectrometer, either as a compressed solid or neat oil. ^{1}H and ^{13}C nuclear magnetic resonance (NMR) spectra were recorded in $CDCl_3$ on a JEOL ECX400 or JEOL ECS400 spectrometer, operating at 400 MHz and 100 MHz, respectively. Chemical shifts (δ) are quoted in parts per million (ppm). The residual solvent peaks were used as references in ^{1}H and $^{13}C\{^{1}H\}$ NMR were δH 7.26 ppm and δC 77.0 ppm respectively. Coupling constants (J) are reported in Hertz (Hz) to the nearest 0.1 Hz. The multiplicity abbreviations used are: s singlet, d doublet, t triplet, br broad, dd double doublet, dt double triplet, td triple doublet, ddd double double doublet and m multiplet. Signal assignment was achieved by analysis of DEPT, COSY and HMQC experiments where required. Mass spectra of trap synthesis products were recorded using positive electrospray ionisation (Pos ESI) on a Bruker compact QTOF MS (compact) mass spectrometer (*m/z* ±0.001 precision, 30000 resolution), unless otherwise stated. Mass spectra of trapping reactions were recorded using positive electrospray ionisation (Pos ESI) on a high resolution solariX XR FTMS (solariX) mass spectrometer (*m/z* ±0.0001 precision, $>10^{7}$ maximum resolution, mass accuracy 600 ppb (internal)), unless stated otherwise.

S2. Chemicals

Methyl 2-(bromomethyl)acrylate (>97%, TCI), 2,2,6,6-tetramethylpiperidine 1-oxyl (TEMPO, 98%, Acros Organics), sodium iodide (>99%, Thermo Scientific), sodium sulfite (≥98%, Sigma-Aldrich), *O*-(benzotriazol-1-yl)-*N*,*N*,*N'*,*N'*-tetramethyluronium hexafluorophosphate (HBTU, 98%, Alfa Aesar), *N*,*N*-diisopropylethylamine (DIPEA, ≥99%, Sigma-Aldrich) cyclohexylamine (>98%, Alfa Aesar) and *N*,*N*-dimethylethylenediamine (≥98%, Sigma-Aldrich) were used for trap synthesis without further purification.

Styrene (99%, Acros Organics), tris(2,2'-bipyrazine)ruthenium(II) hexafluorophosphate (95%, Strem), benzyl mercaptan (99%, Sigma-Aldrich), thiophenol (>99%, Alfa Aesar), dibutylamine (≥98%, Sigma-Aldrich), *N*-chlorosuccinimide (NCS, 98%, Sigma-Aldrich), cyclohexene (≥98%, Fischer Chemical), α-pinene (98%, Sigma-Aldrich), *n*-nonane (99%, Acros Organics), isopentyl nitrite (97%, Alfa Aesar), diisopropylamine (99%, Acros Organics), octanoic acid (≥99%, Sigma-Aldrich), silver nitrate (≥99%, Sigma-Aldrich), bromine (≥95%, Sigma-Aldrich), iodine (≥99.8%, Sigma-Aldrich), *p*-anisic acid (≥99%, Sigma-Aldrich), tribasic potassium phosphate (>97%, Thermo Scientific), sodium acetate (≥99%, Alfa Aesar), iron(II) sulfate heptahydrate (≥99%, Sigma-Aldrich), hydrogen peroxide (30wt.%, Fischer Chemical), thymine (97%, Alfa Aesar), D(+)-glucose (≥99.5%, Sigma-Aldrich) and L-ascorbic acid (99%, Sigma-Aldrich) were used for radical trapping without further purification.

Water (LC-MS grade, ≥99.9%, Fischer Chemical), acetonitrile (LC-MS grade, ≥99.9%, Fischer Chemical), formic acid (LC-MS grade, ≥99%, Fischer Chemical), tetrabutylammonium hexafluorophosphate (98%, Sigma-Aldrich), water-d_2 (99.9 atom % D, Sigma-Aldrich), formic acid-d_2 (95 wt.% in D_2O, 98% atom % D, Sigma-Aldrich) were used for MS characterisation without further purification.

S3. Trap synthesis

S3.1. Methyl 2-(TEMPOmethyl)acrylate

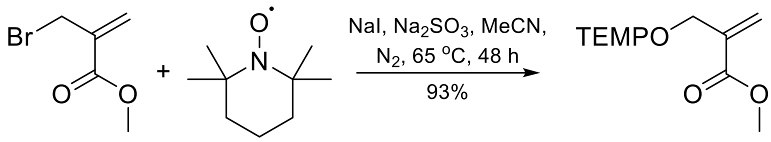

TEMPO **Methyl 2-(TEMPOmethyl)acrylate**

Methyl 2-(bromomethyl)acrylate (1.79 g, 10.0 mmol, 1.0 eq.), (2,2,6,6-tetramethylpiperidin-1-yl)oxyl (TEMPO, 1.88 g, 12.0 mmol, 1.2 eq.), NaI (3.00 g, 20.0 mmol, 2.0 eq.), Na_2SO_3 (3.78 g, 30.0 mmol, 3.0 eq.) were dissolved in MeCN (100 mL). This solution was purged with N_2 and stirred at 65 °C for 48 h. MeCN was then removed *in vacuo*. H_2O (100 mL) was added and product extracted with EtOAc (3×100 mL). The organic phase was dried with $MgSO_4$ and filtered. The solvent was removed *in vacuo*, yielding crude orange oil. This was purified using flash silica column chromatography (visualised using $KMnO_4$ stain) yielding methyl 2-([(2,2,6,6-tetramethylpiperidin-1-yl)oxyl]methyl)acrylate as yellow oil (methyl 2-(TEMPOmethyl)acrylate, 2.37 g, 93%).

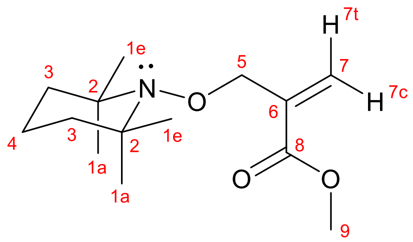

R_f: 0.33 (2%Et_2O/DCM). CHN: C, 65.4; H, 9.9; N, 5.1 (found); C, 65.9; H, 9.9; N, 5.5 (calc. for $C_{14}H_{25}NO_3$). IR: ν_{max} / cm^{-1} 2980 (CH), 2900 br (OH), 1720 (C=O), 1630 (C=C), 1150 (C−O), 1060 (C−O). ^1H, ^{13}C NMR (400, 100 MHz, $CDCl_3$): δ_H 6.28 (dt, *J* 1.8, 1.6 Hz, 1H, H_{7c}), 5.91 (td, *J* 1.9, 1.8 Hz, 1H, H_{7t}), 4.49 (dd, *J* 1.9, 1.6 Hz, 2H, H_5), 3.75 (s, 3H, H_9), 1.55-1.48 (m, 1H, H_4), 1.47-1.42 (m, 4H, H_3) 1.35-1.28 (m, 1H, H_4), 1.15 (s, 6H, H_{1a}), 1.10 (s, 6H, H_{1e}); δ_C 166.5 (C_8), 137.0 (C_6), 125.4 (C_7), 74.7 (C_5), 60.1 (C_2), 51.9 (C_9), 39.8 (C_3), 32.9 (C_{1a}), 20.4 (C_{1e}), 17.2 (C_4). MS (Pos ESI): *m/z* 256.191 ($[M+H]^+$, 100%); 256.191 (calc. for $C_{14}H_{26}NO_3$, $[M+H]^+$).

S3.2. 2−(TEMPOmethyl)acrylic acid

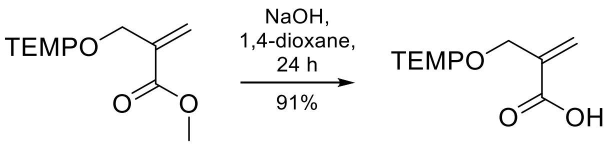

Methyl 2-(TEMPOmethyl)acrylate **2-(TEMPOmethyl)acrylic acid**

Methyl 2-(TEMPOmethyl)acrylate (1.58 g, 6.20 mmol, 1.0 eq.) was placed in 1,4-dioxane (40 mL), aqueous NaOH (1.0 M, 40 mL, 40 mmol, 6.5 eq.) was added and the solution was stirred for 24 h. The resultant solution was acidified (pH 5) with aqueous HCl (2.0 M, 20 mL, 40 mmol, 6.5 eq.) and the product was extracted with EtOAc (3×40 mL). The organic phase was washed with brine (40 mL), dried with $MgSO_4$ and filtered. The solvent was removed *in vacuo*, yielding crude golden oil. This was purified using flash silica column chromatography

(visualised using $KMnO_4$ stain) yielding white crystalline 2-(TEMPOmethyl)acrylic acid (1.36 g, 91%).

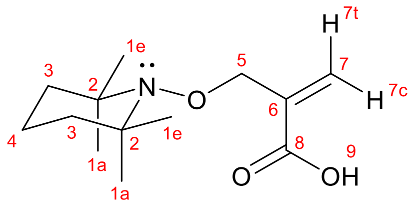

R_f: 0.32 (0.1%AcOH/5%MeOH/DCM). Mp: 94.7-96.1 °C. CHN: C, 64.3; H, 9.8; N, 5.8 (found); C, 64.7; H, 9.6; N, 5.8 (calc. for $C_{13}H_{23}NO_3$). IR: ν_{max} / cm^{-1} 2980 (CH), 1700 (C=O), 1640 (C=C), 1360 (C−O); ^1H, ^{13}C NMR (400, 100 MHz, $CDCl_3$): δ_H 6.40 (dt, *J* 3.3, 1.8 Hz, 1H, H_{7c}), 5.96 (dt, *J* 3.3, 1.6 Hz, 1H, H_{7t}), 4.53 (dd, *J* 1.8, 1.6 Hz, 2H, H_5), 1.61-1.55 (m, 1H, H_4), 1.53-1.48 (m, 4H, H_3) 1.38-1.34 (m, 1H, H_4), 1.19 (s, 6H, H_{1a}), 1.14 (s, 6H, H_{1e}); δ_C 173.3 (C_8), 137.5 (C_6), 128.1 (C_7), 74.6 (C_5), 60.6 (C_2), 39.6 (C_3), 32.6 (C_{1a}), 20.6 (C_{1e}), 17.1 (C_4). MS (Pos ESI): *m/z* 242.175 ($[M+H]^+$, 100%), 264.157 ($[M+Na]^+$, 16%); 242.176 (calc. for $C_{13}H_{24}NO_3$, $[M+H]^+$); (Neg ESI): *m/z* 240.160 ($[M-H]^-$, 100%); 240.160 (calc. for $C_{13}H_{22}NO_3$, $[M-H]^-$).

S3.3. Standard amide coupling reaction procedure

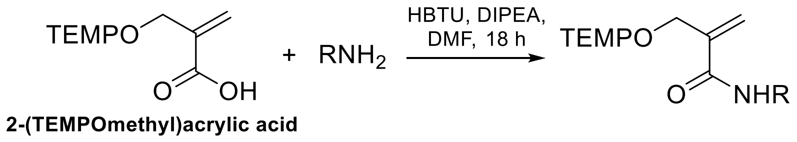

2-(TEMPOmethyl)acrylic acid (1.0 eq.), *O*-(benzotriazol-1-yl)-*N*,*N*,*N'*,*N'*-tetramethyluronium hexafluorophosphate (HBTU, 1.1 eq.), *N*,*N*-diisopropylethylamine (DIPEA, 2.0 eq.) and amine (1.0 eq.) were dissolved in *N,N*-dimethylformamide (DMF, 5.0 L mol^{-1}) and stirred for 18 h. The solvent was then removed *in vacuo*. Saturated aqueous $NaHCO_3$ (10 L mol^{-1}) was added and product was extracted with EtOAc (3×10 L mol^{-1}). The combined organic layers were washed with brine (3×10 L mol^{-1}), dried with $MgSO_4$ and filtered. The solvent was removed *in vacuo* yielding crude product. This was purified using flash silica column chromatography (visualised using $KMnO_4$ stain) yielding pure product.

S3.4. CHANT

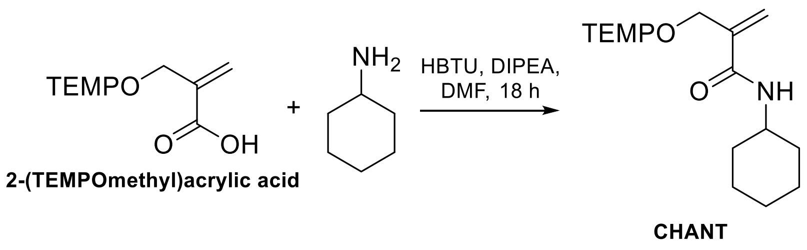

Standard amide coupling procedure (S3.3) was performed with 2-(TEMPOmethyl)acrylic acid (483 mg, 2.00 mmol, 1.0 eq.), HBTU (834 mg, 2.20 mmol, 1.1 eq.), DIPEA (517 mg, 4.00 mmol, 2.0 eq.), cyclohexylamine (198 mg, 2.00 mmol, 1.0 eq.) and DMF (10.0 mL) yielding white *N*-cyclohexyl-2-{[(2,2,6,6-tetramethylpiperidin-1-yl)oxy]methyl}acrylamide needles (CHANT, 497 mg, 77% or 65% overall).

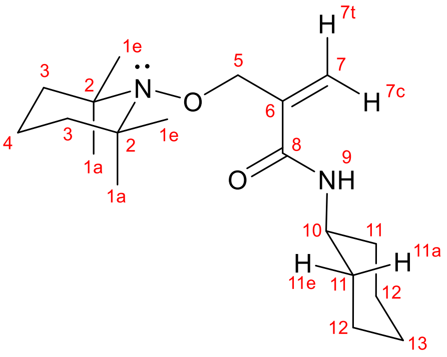

R_f: 0.32 (20%EtOAc/PET ether). Mp: 99.0-100.1 °C. CHN: C, 70.6; H, 10.7; N, 8.4 (found); C, 70.8; H, 10.6; N, 8.7 (calc. for $C_{19}H_{34}N_2O_2$). IR: ν_{max} / cm^{-1} 3340 (NH), 2930 (CH), 1650 (C=O), 1610 (C=C), 1540 (NH). ^{1}H, ^{13}C NMR (400, 100 MHz, $CDCl_3$): δ_H 6.62 (br d, *J* 6.9 Hz, 1H, H_9), 6.09 (d, *J* 1.8 Hz, 1H, H_{7c}), 5.48 (dt, *J* 1.8, 1.0 Hz, 1H, H_{7t}), 4.48 (d, *J* 1.0 Hz, 2H, H_5), 3.90-3.79 (m, *J* 7.2, 6.9 Hz, 1H, H_{10}), 2.00 (ddd, *J* 12.4, 7.2, 3.7 Hz, 2H, H_{11e}), 1.74 (dt, *J* 13.7, 3.7 Hz, 2H, H_{12e}), 1.60-1.53 (m, 1H, H_{13e}), 1.52-1.48 (m, 4H, H_3), 1.48-1.44 (m, 1H, H_4), 1.39 (dt, *J* 13.7, 3.7 Hz, 2H, H_{12a}), 1.38-1.32 (m, 1H, H_4), 1.20 (s, 6H, H_{1a}), 1.16-1.13 (m, 1H, H_{13a}), 1.13 (m, *J* 12.4, 7.2 Hz, 2H, H_{11a}) 1.11 (s, 6H, H_{1e}); δ_C 165.7 (C_8), 139.7 (C_6), 123.7 (C_7), 77.5 (C_5), 59.8 (C_2), 48.0 (C_{10}), 39.5 (C_3), 33.2 (C_{1a}), 32.9 (C_{11}), 25.4(C_{12}), 24.8 (C_{13}), 20.1 (C_{1e}), 16.8 (C_4). MS (Pos ESI): *m/z* 323.270 ($[M+H]^+$, 100%), 345.252 ($[M+Na]^+$, 4%); 323.270 (calc. for $C_{19}H_{35}N_2O_2$, $[M+H]^+$).

S3.5. DEADANT

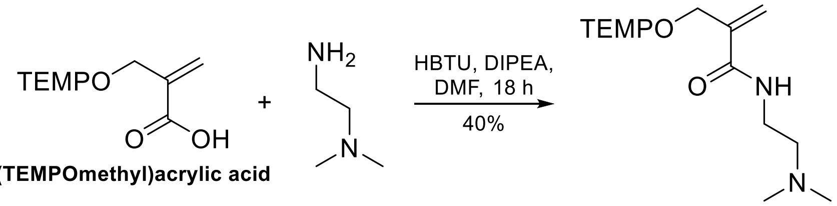

2-(TEMPOmethyl)acrylic acid

DEADANT

Standard amide coupling procedure (S3.3) was performed with 2-(TEMPOmethyl)acrylic acid (482 mg, 2.00 mmol, 1.0 eq.), HBTU (835 mg, 2.20 mmol, 1.1 eq.), DIPEA (517 mg, 4.00 mmol, 2.0 eq.) *N,N*-dimethylethylenediamine (177 mg, 2.00 mmol, 1.0 eq.) and DMF (10.0 mL) yielding colourless *N'*-*N,N*-dimethylethyleneamino-2-{[(2,2,6,6-tetramethylpiperidin-1-yl)oxy]methyl}acrylamide oil (DEADANT, 251 mg, 40% or 34% overall).

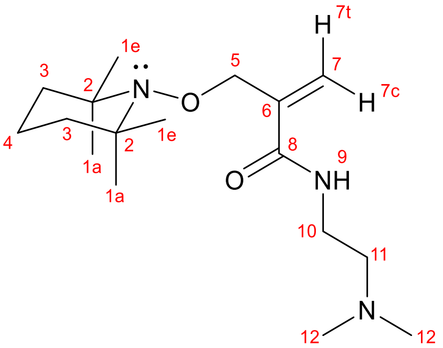

R_f: 0.29 (2%NH_4OH/5%MeOH/DCM). CHN: C, 65.7; H, 11.0; N, 13.5 (found); C, 65.6; H, 10.7; N, 13.5 (calc. for $C_{17}H_{33}N_3O_2$). IR: ν_{max} / cm^{-1} 3345 (NH), 2932 (CH), 1660 (C=O), 1616 (C=C), 1532 (NH), 1056 (CN), 1043 (CN). ^{1}H, ^{13}C NMR (400, 100 MHz, $CDCl_3$): δ_H 7.01 (br s, 1H,

H_9), 6.07 (dt, *J* 1.5, 1.1 Hz, 1H, H_{7c}), 5.51 (dt, *J* 1.5, 0.6 Hz, 1H, H_{7t}), 4.50 (dd, *J* 1.1, 0.6 Hz, 2H, H_5), 3.42 (td, *J* 6.0, 5.2 Hz, 2H, H_{10}), 2.45 (t, *J* 6.0 Hz, 2H, H_{11}), 2.20 (s, 6H, H_{12}), 1.65-1.50 (m, 1Hf, H_4), 1.50-1.43 (m, 4H, H_3) 1.37-1.30 (m, 1H, H_4), 1.18 (s, 6H, H_{1a}), 1.10 (s, 6H, H_{1e}); δ_C 167.0 (C_8), 139.9 (C_6), 123.4 (C_7), 77.5 (C_5), 60.0 (C_2), 57.8 (C_{11}), 45.2 (C_{12}), 39.8 (C_3), 37.1 (C_{10}), 33.1 (C_{1a}), 20.2 (C_{1e}), 17.2 (C_4). MS (Pos ESI): *m/z* 312.266 ($[M+H]^+$, 100%), 334.247 ($[M+Na]^+$, 4%); 312.265 (calc. for $C_{17}H_{34}N_3O_2$, $[M+H]^+$).

S4. Trap properties and stability studies

S4.1. Free TEMPO concentration

Concentration of free TEMPO in pure CHANT was quantified using electron paramagnetic resonance (EPR) spectroscopy. This was to determine if initial side reactions could be caused by free TEMPO, potentially making traps non-innocent in main reaction cycles.

Double integrals of EPR spectra of CHANT solution (1.00 mM) and standard TEMPO solution (0.100 mM) in MeCN were used to determine radical concentration. EPR spectra were recorded at X-band on a JEOL X320 spectrometer using 1 G modulation width and 1 mW power.

EPR spectra indicated ~0.05mol.% free TEMPO relative to CHANT (Figure S1). This low relative concentration implied that initial side reactions caused by TEMPO would be minimal. 0.05% TEMPO concentration relative to trap was assumed to be constant for all unreacted traps.

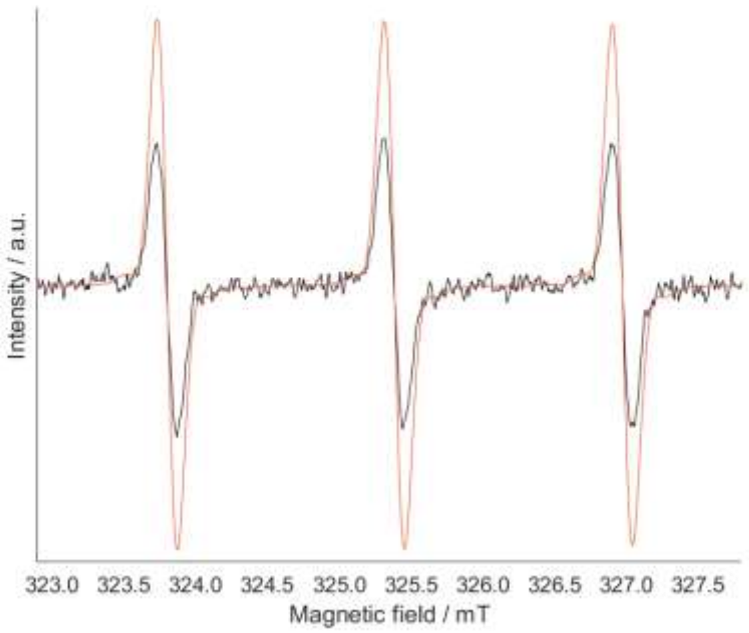

Figure S1: EPR spectra recorded for CHANT (1.00 mM, scaled×100, black) and TEMPO (0.100 mM, orange), indicating CHANT had free TEMPO content ~0.05%.

S4.2. Oxidation and reduction

Oxidation and reduction processes of CHANT were investigated using cyclic voltammetry (CV), to determine CHANT resistance to oxidation and reduction and hence robustness.

Cyclic voltammetry was performed using a 5 mL electrochemical cell containing platinum wire working and counter electrodes and a Ag/AgCl reference electrode. CHANT analyte (1.00 mM) and Bu_4NPF_6 electrolyte (100 mM) in MeCN (2.5 mL total volume) were added to the cell and a cyclic voltammogram was recorded over a range of -1 V to +2 V at a scan rate of 100 mV s^{-1}.

Cyclic voltammograms showed that CHANT did not undergo any significant oxidative or reductive processes under these conditions. This indicated CHANT was robust under mildly oxidative and reductive conditions.

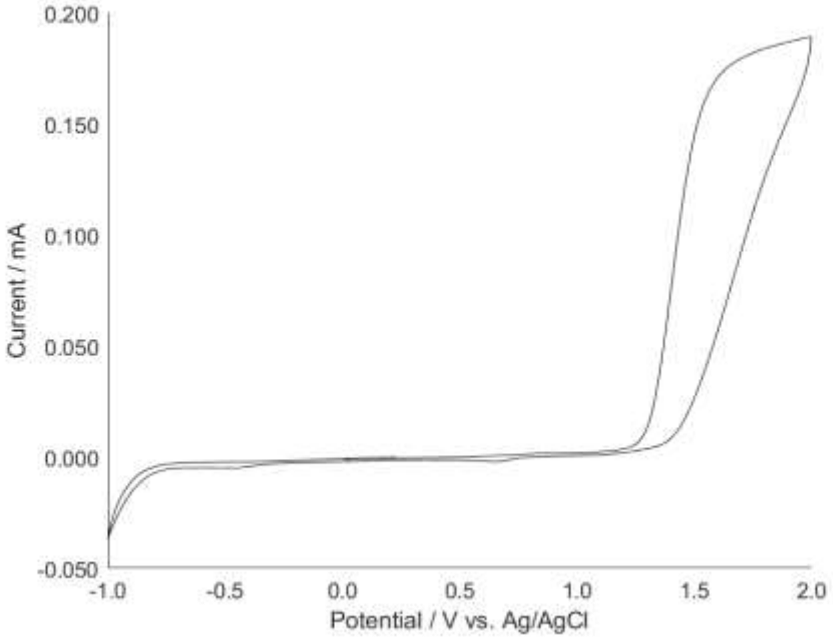

Figure S2: Cyclic voltammogram of CHANT (1.00 mM), using MeCN as solvent and Bu_4NPF_6 (100 mM) as electrolyte and Pt wire working and counter electrodes and a Ag/AgCl reference electrode.

S4.3. Michael addition

CHANT susceptibility to Michael addition by polar solvents and weak bases was explored. For this, CHANT was subjected to polar solvents and weak bases and monitored using ^1H NMR spectroscopy, to determine trap robustness and ensure non-radical species did not produce false positives.

CHANT (~10 mmol dm^{-3}) dissolved in CD_3OD showed no detectable decay after three weeks.

CHANT (~10 mmol dm^{-3}) in the presence of diisopropylamine (~1:5 molar ratio) and dissolved in CD_3OD, showed no detectable decay after 24 h but some decay after three weeks (Figure S5). From NMR spectra observations, it was believed that diisopropylamine added to the CHANT double bond, without the loss of TEMPO (Figure S3). This was because NMR spectra showed decreasing intensity exclusively for peaks corresponding to alkene and allyl hydrogen atoms, whilst new peaks were observed with increasing intensity and hence were assigned to the decay product (Figure S4). These new peaks were assigned as follows: ^1H NMR (400 MHz, CD_3OD): δ_H 3.92 (d, *J* 8.2 Hz, 1H, H_{5p}), 3.82 (d, *J* 8.2 Hz, 1H, H_{5p}), 2.90-2.74 (m, 1H, H_6), 2.82 (d, *J* 11.9 Hz, 1H, H_{7p}), 2.68 (d, *J* 11.9 Hz, 1H, H_{7p}). Meanwhile, intensity of the peaks corresponding to the 4×CH_3 in the TEMPO moiety did not change.

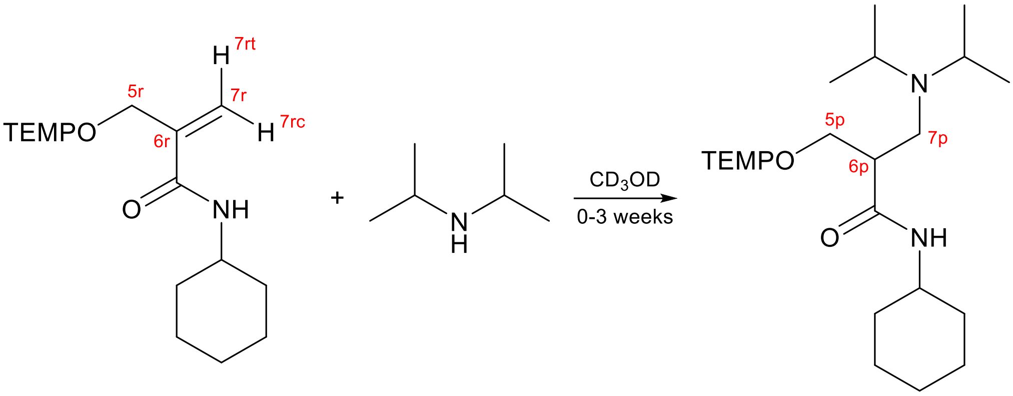

Figure S3: Michael addition of diisopropylamine to CHANT, forming a decay product.

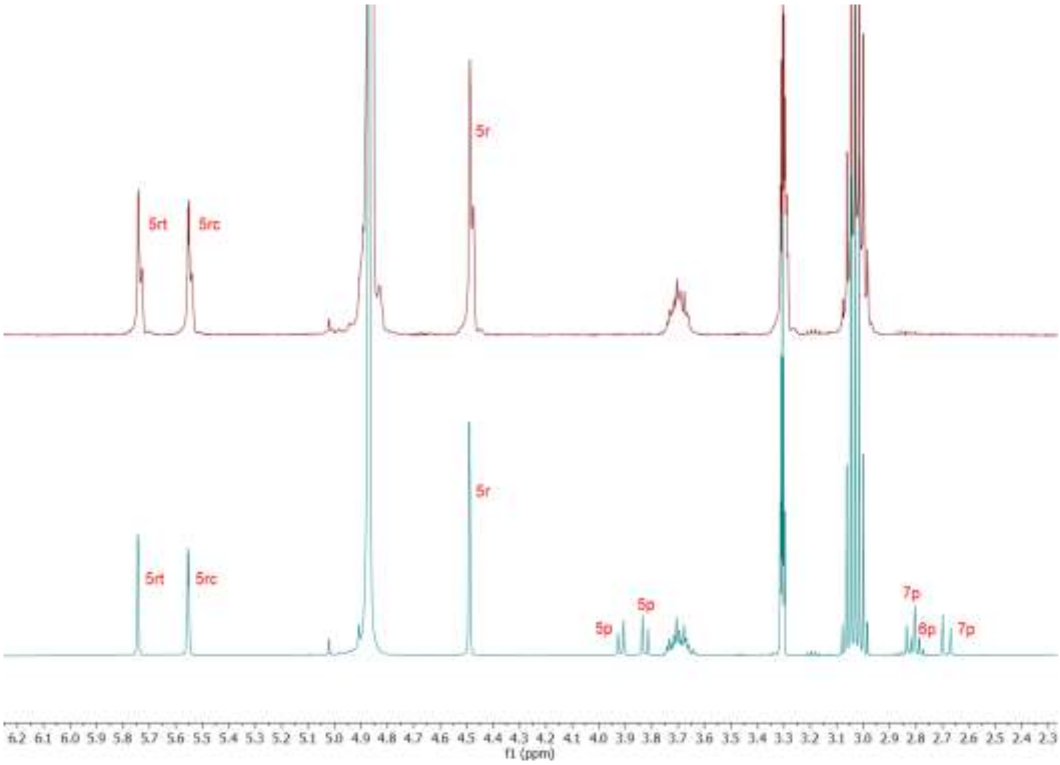

Figure S4: Stacked ^{1}H NMR spectra of CHANT in the presence of diisopropylamine after 0 h (top) and 3 weeks (bottom), showing peaks which decrease in intensity (5r-7r) and new signals corresponding to the decay product (5p-7p).

Relative intensities of CHANT and its decay product, referenced to CHD_2OD solvent peak, were mapped over time, indicating ~25% CHANT decay and ~25% decay product yield after three weeks (Figure S5). This showed that CHANT was resistant to polar solvents and weak nucleophiles but more importantly, indicated that TEMPO loss, i.e. novel radical trapping, did not occur for non-radicals. This was assumed to be true for all traps.

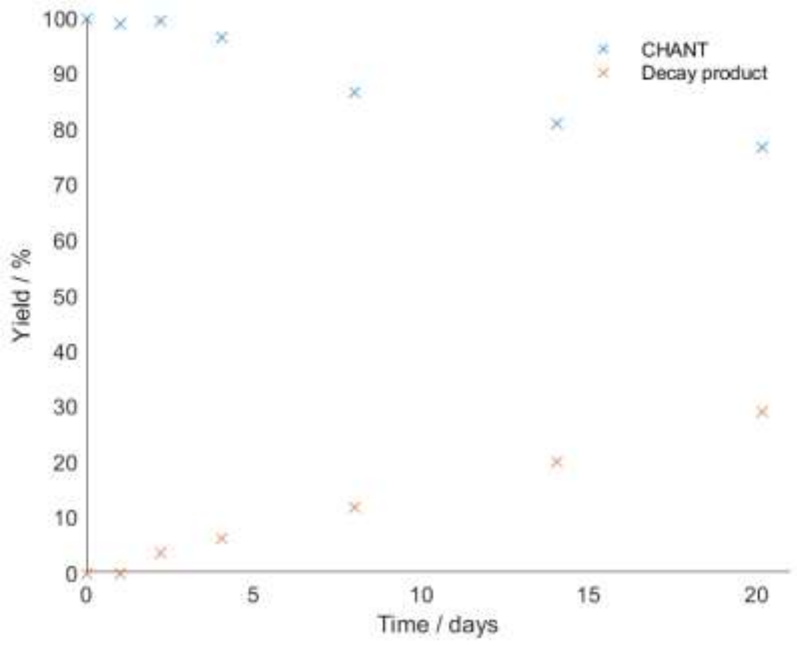

Figure S5: Decay of CHANT in the presence of diisopropylamine dissolved in CD_3OD, monitored using NMR spectroscopy.

S4.4. Absorbance of blue LED light (455 nm)

Absorbance of ultraviolet-visible light (250-800 nm) by CHANT (10.0 mM) was measured using ultraviolet-visible (UV-Vis) spectroscopy. This was to ensure CHANT did not absorb light from the blue LEDs (455 nm) used for thiol-ene addition photoinitiation, which may have caused CHANT to be non-innocent in the thiol-ene addition radical cycle.

MeCN was used as solvent in all thiol-ene addition reactions and therefore, was used as solvent in the sample and a blank reference in the UV-Vis spectroscopy conducted upon CHANT. UV-Vis spectra were recorded using a Shimadzu UV-Vis Spectrophotometer UV-1800 system.

The resulting UV-Vis spectrum showed CHANT did not absorb light above 350 nm, ensuring CHANT could not contribute to photoinitiation (Figure S6).

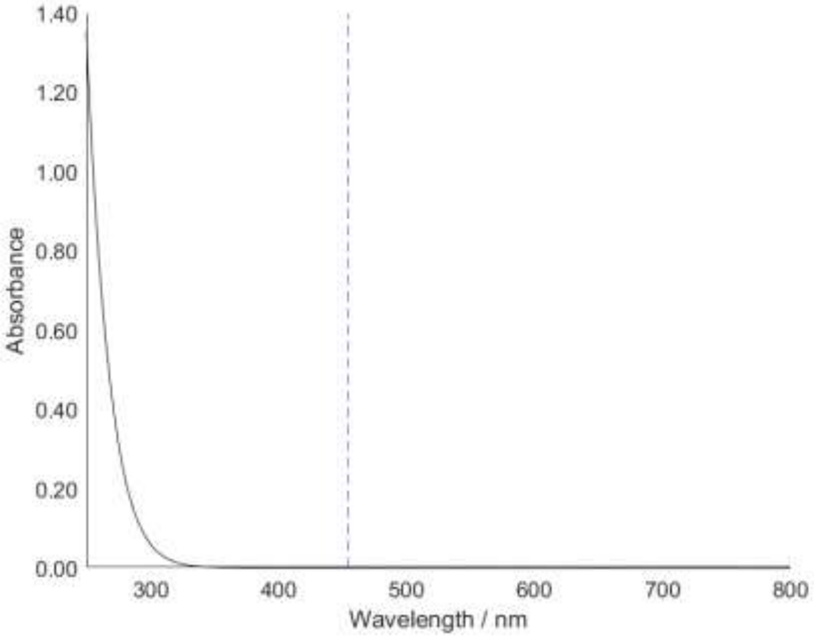

Figure S6: UV-Vis spectrum of CHANT (10.0 mM) in MeCN. Wavelength of blue LED irradiation (455 nm), used for the radical trapping in thiol-ene addition reaction, is indicated (blue dashed line).

S4.5. MS calibration curves

MS calibration curves were obtained for CHANT, where CHANT concentration was mapped against MS intensity of $[CHANT+H]^+$ (Figure S7). Mass spectra showed a linear relationship between CHANT concentration and MS intensity until ~1.0 μM and a gently curved relationship between ~1.0-10.0 μM. Unreacted trap standard was typically 10.0 μM. At concentrations of >25 μM, $[CHANT+H]^+$ intensity decreased rapidly and a fragment was observed, corresponding to a TEMPO ion. This indicated that at high concentrations, trap was unstable in MS.

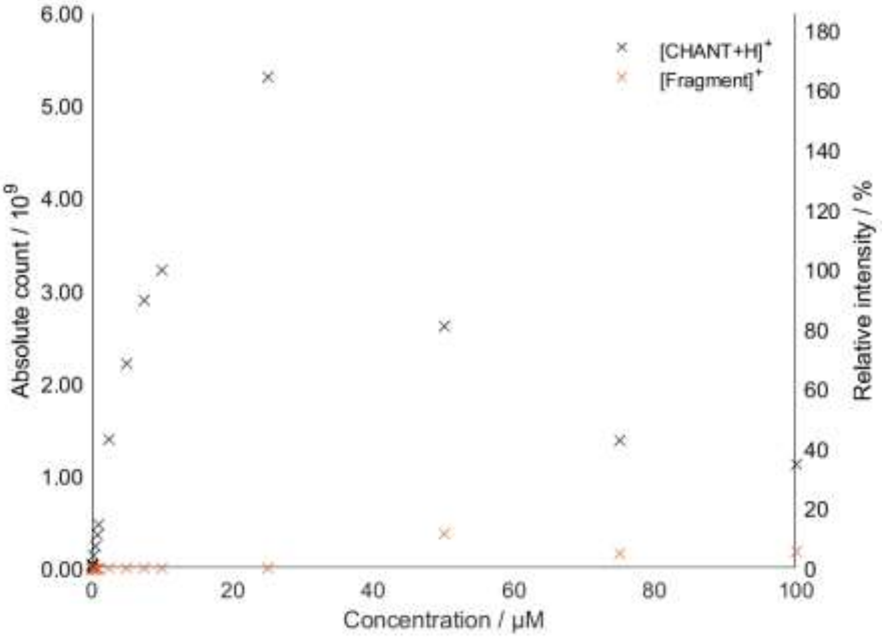

Figure S7: Calibration curve of MS intensity of MS peak corresponding to [CHANT+H]$^+$ (*m/z* 323.270) at different CHANT concentrations (black), showing rapidly decreasing intensity >25 μM due to CHANT fragmentation. The most intensely observed fragment peak (*m/z* 140.144) appears to correspond to a 2,2,6,6-tetramethylpiperidine cation (orange). Intensity is relative to 10 μM CHANT standard.

An MS calibration curve was also obtained for trapped PhS$^{\bullet}$. Trapped PhS$^{\bullet}$ was mainly observed as a sodiated MS adduct, [PhS−ART+Na]$^+$ (*m/z* 298.124). From henceforth, ART (allyl radical trap) denotes the trap residue, so for instance PhS−ART represents a trapped PhS$^{\bullet}$ radical (e.g., −ART is $-CH_2C(CONHC_6H_{11})=CH_2$ for CHANT). [CHANT+H]$^+$ (*m/z* 323.270) had a similar *m/z* to [PhS−ART+Na]$^+$ and therefore, mass spectrometer parameters should not have caused significant differences in ionisation efficiency of these adducts. However, when compared to the CHANT calibration curve, MS adducts had significantly poorer intensity than for CHANT. This suggested that PhS−ART and similarly low basicity CHANT-trapped radicals ionised significantly less efficiently than CHANT itself, likely due to CHANT having a basic 3° N in its TEMPO group (Figure S8). Likewise, this suggested that TEMPO-trapped radicals ionised more efficiently than low basicity CHANT-trapped radicals. This had important implications for using MS intensity for quantification of trapped radical concentration.

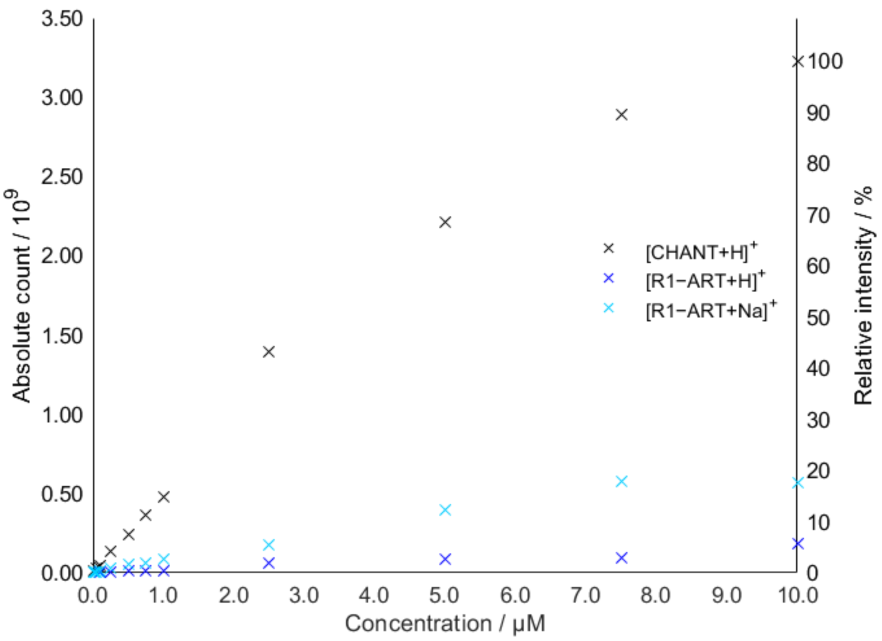

Figure S8: Calibration curve of MS intensity of MS peaks corresponding to $[CHANT+H]^+$ (*m/z* 323.270, black), $[PhS{-}ART+H]^+$ (*m/z* 276.142, dark blue, PhS = R1) and $[PhS{-}ART+Na]^+$ (*m/z* 298.124, light blue) at different analyte concentrations. This indicated CHANT ionised more efficiently than low basicity CHANT-trapped radicals. ART denotes the trap residue, so R−ART represents a trapped $R^\bullet$ radical (e.g., −ART is $-CH_2C(CONHC_6H_{11})=CH_2$ for CHANT). Intensity is relative to 10 μM CHANT standard.

S5. Radical trapping procedures

S5.1. General

Liquid phase trapping usually involved including trap into a procedure for a liquid phase radical reaction sourced from the literature. Gas phase trapping usually involved bubbling a gaseous radical reaction stream through a solution containing trap, where a procedure for the gas stream was sourced from literature. MS characterisation and analysis were then performed upon resultant reaction mixtures (S6).

Five reactions are discussed in detail in the main paper: thiol-ene addition (S5.2); Hofmann-Löffler-Freytag (HLF) reaction (S5.3); cyclohexene and α-pinene ozonolysis (S5.4) and •OH-initiated *n*-nonane degradation (S5.5). The results of several additional reactions are given in this supplementary information: Barton reaction (S5.6); Hunsdiecker reaction (S5.7); decarboxylative aromatic iodination (S5.8) and aqueous •OH-initiated degradation of methanol (S5.9), thymine, glucose and ascorbic acid (S5.10).

S5.2. Thiol-ene addition

S5.2.1. Trapping reaction

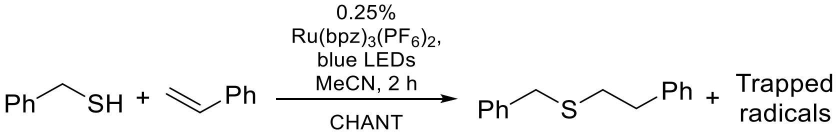

The procedure for trapping in radical thiol-ene addition was adapted from a literature procedure[S1]. Styrene (115 µL, 100 µmol, 1.00 eq.) and $Ru(bpz)_3(PF_6)_2$ (0.22 mg, 0.25 µmol, 0.0025 eq.) were placed in a transparent 2 mL sample vial and dissolved in dry MeCN (0.50 mL, 0.20 M). If undertaking radical trapping, CHANT (32.2 mg, 10 µmol, 0.10 eq.) was also added. Benzyl mercaptan (235 µL, 200 µmol, 2.00 eq.) was added and an aliquot removed (5.0 µL). The remaining solution was exposed to blue LEDs (60 W, 455 nm) for 2 h. An aliquot was removed (5.0 µL) and all aliquots were MS characterised.

S5.2.2. Isolation of CHANT-trapped PhS• (*N*-cyclohexyl-2-[(phenylsulfanyl)methyl]acrylamide)

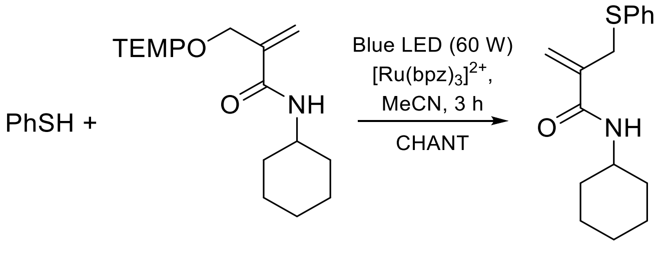

The procedure for CHANT-trapped PhS• synthesis was adapted from literature[S1]. Thiophenol (41 µL, 400 µmol, 4.0 eq.), CHANT (32.2 mg, 100 µmol, 1.0 eq.) and $Ru(bpz)_3(PF_6)_2$ (2.22 mg, 2.50 µmol, 0.0025 eq.) were placed in a transparent 2 mL sample vial and dissolved in dry MeCN (0.50 mL, 0.20 M). The resultant solution was irradiated with blue LEDs (60 W, 455 nm). Aliquots (5.0 µL) were removed regularly to monitor the reaction using MS, with eight aliquots having been removed in total (40.0 µL) when the reaction was stopped after 3 h. Solvent was removed *in vacuo* and the resultant purified using flash silica column chromatography

(visualised using UV light or $KMnO_4$ stain) yielding white *N*-cyclohexyl-2-[(phenylsulfanyl)methyl]acrylamide semi-solid (17.3 mg, 63%).

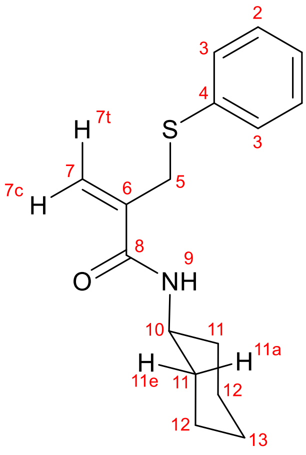

R_f: 0.23 (2%Et_2O/DCM). ^1H, ^{13}C NMR (400, 100 MHz, $CDCl_3$): δ_H 7.35-7.23 (m, 4H, H_2, H_3), 7.22-7.16 (m, 1H, H_1), 6.00 (br d, *J* 6.4 Hz, 1H, H_9), 5.58 (s, 1H, H_{7c}), 5.23 (s, 1H, H_{7t}), 3.77 (s, 2H, H_5), 3.87-3.78 (m, *J* 7.2, 6.4 Hz, 1H, H_{10}), 1.92 (ddd, *J* 12.4, 7.2, 3.7 Hz, 2H, H_{11e}), 1.69 (dt, *J* 13.7, 3.8 Hz, 2H, H_{12e}), 1.59 (dt, *J* 12.8, 3.8 Hz, 1H, H_{13e}), 1.37 (dt, *J* 13.7, 3.8 Hz, 2H, H_{12a}), 1.25-1.09 (m, 3H, H_{11a} , H_{13a}); δ_C 166.7 (C_8), 140.7 (C_6), 131.0 (C_2), 129.0 (C_3), 127.1 (C_1), 120.7 (C_7), 48.4 (C_{10}), 36.7 (C_5), 33.1 (C_{11}), 25.6(C_{13}), 24.9 (C_{12}), 134.9 (C_4). MS (Pos ESI): *m/z* 298.125 ($[M+Na]^+$, 100%), 276.145 ($[M+H]^+$, 33%), 573.261 ($[2M+Na]^+$, 8%); 298.124 (calc. for $C_{16}H_{21}NOSNa$, $[M+Na]^+$).

S5.3. Hofmann-Löffler-Freytag (HLF) reaction

S5.3.1. Precursor synthesis

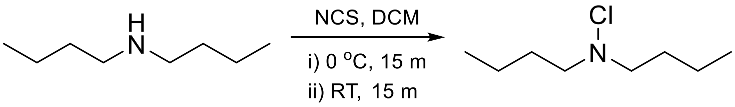

The procedure for *N*-chlorodibutylamine synthesis was adapted from literature[S2, S3]. Dibutylamine (1.29 g, 10.0 mmol, 1.0 eq.) was sparged in dry degassed DCM (25 mL) and placed under N_2. *N*-Chlorosuccinimide (NCS, 1.47 g, 11.0 mmol, 1.1 eq.) was added and the reaction stirred at 0 °C for 15 min, then at room temperature for 15 min. Solvent was then removed *in vacuo* and the residue was washed in *n*-pentane (2×25 mL) and filtered, yielding colourless *N*-chlorodibutylamine oil (1.48 g, 90%).

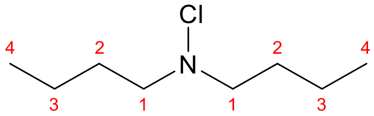

^1H, ^{13}C NMR (400, 100 MHz, $CDCl_3$): δ_H 2.94-2.88 (m, *J* 7.3 Hz, 4H, H_1), 1.69-1.59 (m, *J* 7.3, 7.3 Hz, 4H, H_2), 1.36 (dt, *J* 7.3, 7.3 Hz, 4H, H_3), 0.93 (t, *J* 7.3 Hz, 6H, H_4); δ_C 64.2 (C_1), 30.2 (C_2), 20.2 (C_3), 14.1 (C_4). MS (Pos ESI): *m/z* 130.159 ($[M-Cl+2H]^+$, 100%); 163.113 (calc. for $C_8H_{18}NCl$). Obtained values were consistent with literature[S4].

S5.3.2. Trapping reaction

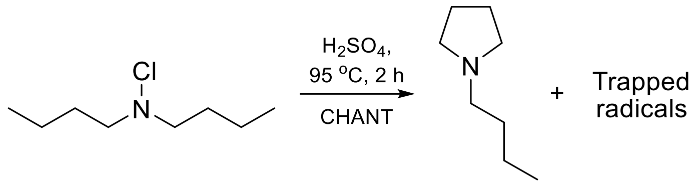

The procedure for trapping in the HLF reaction was adapted from literature[S5]. *N*-Chlorodibutylamine (81.8 mg, 0.500 mmol, 1.0 eq.) was dissolved in H_2SO_4 solution (0.25 M, 0.50 mL, 0.125 mmol, 0.25 eq.). If undertaking radical trapping, CHANT (16.1 mg, 0.050 mmol, 0.10 eq.) was added. For all reaction mixtures, an aliquot was removed (50 µL) and the remaining solution heated at 95 °C for 2 h, whilst stirring. This was let cool and an aliquot removed (50 µL). All aliquots were diluted with water (0.5 mL) and neutralised (pH 7) with NaOH (1.0 M, 13 µL, 0.125 mmol, 0.25 eq.), extracted with Et_2O (2×0.5 mL), solvent removed *in vacuo* and MS characterised.

S5.4. Alkene ozonolysis

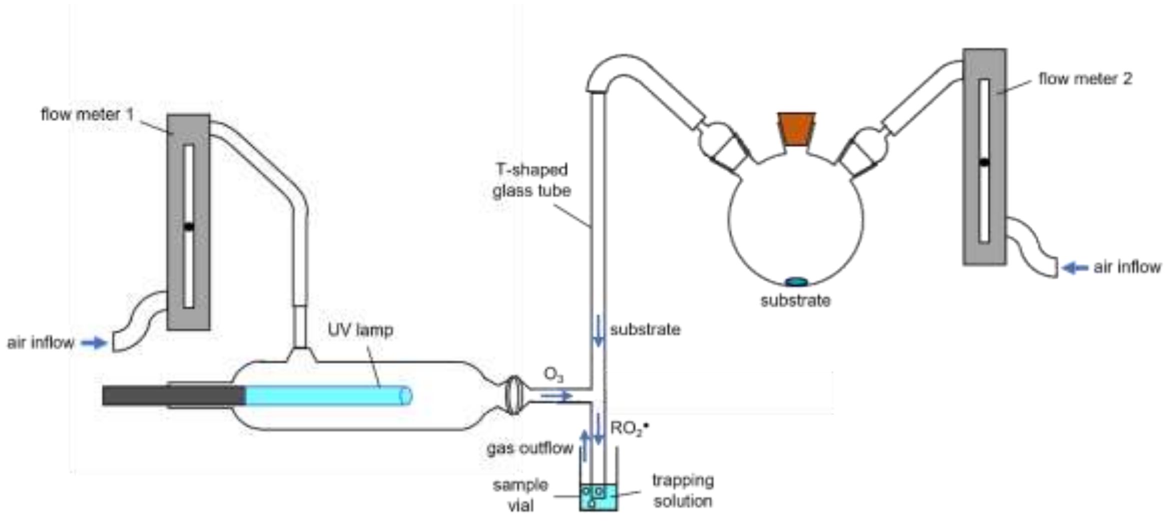

Figure S9: Set-up used for radical trapping in alkene ozonolysis.

A set-up was designed for radical trapping in alkene ozonolysis (Figure S9). The system was used at room temperature (293 K) and pressure (RTP). The T-shaped glass tube used for mixing ozone with alkene vapour had a 0.60 cm internal diameter and sample vial with trapping solution had a 2.0 cm internal diameter. Different length T-shaped glass tubes could be used to increase residence time for substrate reaction with O_3, with typical residence length set at 5.0 cm, resulting in a residence volume of 1.41 cm^3. Liquid substrate was placed as shown, with a sufficient volume to avoid complete evaporation. Trap functionality was chosen as required, however typically CHANT was used. [Trap] was set between 50-5000 µM in 2.0 mL MeCN but was typically 500 µM. The T-shaped glass tube outlet was placed at the bottom of the trapping solution, meaning the outlet lay 6 mm below the trapping solution surface. Ozone was prepared using a low-pressure mercury Pen-Ray UV lamp, with the current and power output kept constant for all experiments. The flow rate through the trapping solution was set at 1.5 L min^{-1}, to ensure rapid but controllable bubbling. This typically resulted in a residence time of 56.5 ms. Flow rate through each bubbler was adjusted as required, but was typically set at an equal 0.75 L min^{-1} through each flow meter. Under these standard conditions and in absence of substrate or trapping solution, $[O_3]$ was measured to be 118 ppmv

(2.96×10^{15} molec. cm^{-3}), using a pre-calibrated Thermo Scientific model 49i ozone meter. Reaction time was varied as required, but was typically 10 min. After trapping completion, trapping solution solvent was carefully removed *in vacuo* (30 ^{o}C, 100 mbar, ~10 min) and the residue was MS characterised. These standard alkene ozonolysis conditions had been optimised.

The standard alkene ozonolysis set-up was used with cyclohexene (3 mL) and α-pinene (1 mL), respectively. For control experiments, one required component for trapped radical generation was omitted at a time: substrate, air (replaced with N_2), UV and trap.

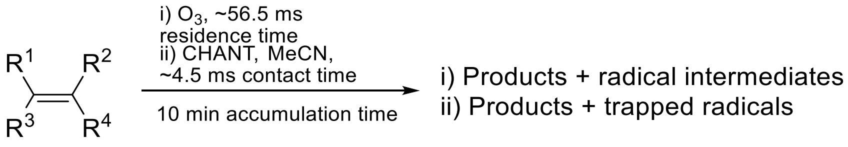

S5.5. <u>Gaseous •OH-initiated *n*-nonane degradation</u>

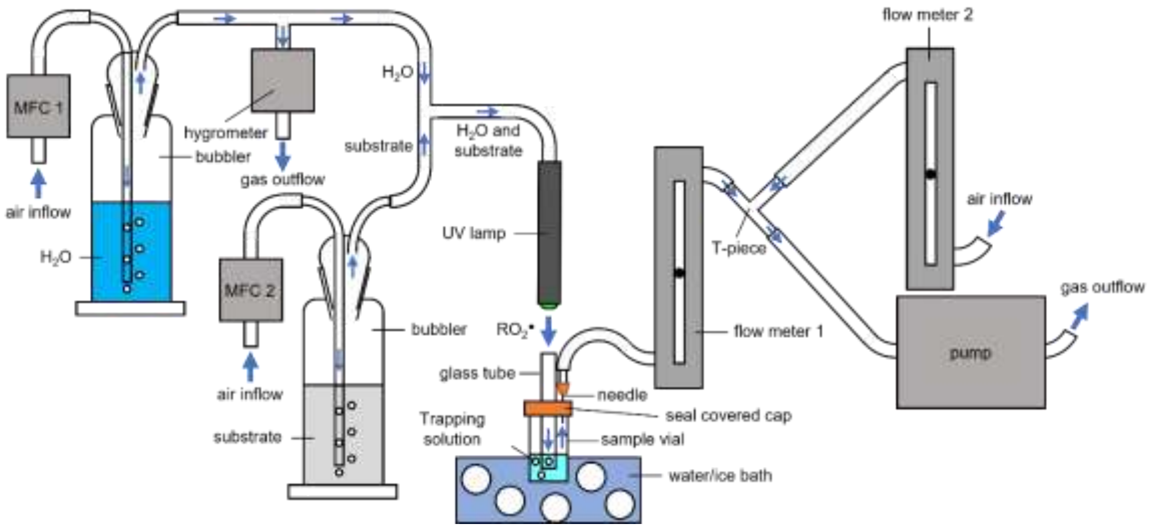

Figure S10: Set-up used for radical trapping of •OH-initiated *n*-nonane degradation.

A set-up was designed for radical trapping in •OH-initiated *n*-nonane degradation (Figure S10). The water photolysis system (the set-up prior and including UV lamp) was calibrated offline in the absence of substrate. This involved using fluorescence assay by gas expansion (FAGE) to measure the range of [$HO_x^•$] ($HO_x^•$ = •OH + $HO_2^•$) produced depending on photon flux (measured using N_2O actinometry), irradiation time and [H_2O] (measured using a hygrometer), as described by Onel *et al.*[S6]. This calibration allowed accurate online [$HO_x^•$] calculation.

The system was used at room temperature (293 K) and pressure (RTP). The Hg Pen-Ray UV lamp (184.9 nm) had a 1.6 cm^2 cross-sectional area, the glass tube had a 0.60 cm internal diameter and the sample vial had a 2.0 cm internal diameter. The length between the UV lamp centre and outflow was 3.8 cm, resulting in a 6.1 cm^3 average residence volume for [$HO_x^•$] inside the UV lamp. Different length glass tubes could be used to increase the residence time for •OH reaction with the substrate, with typical residence length set at 8.0 cm, resulting in a residence volume 2.3 cm^3. The radical trap was selected as discussed in the main paper. [Trap] was set between 50-5000 μM in 2.0 mL MeCN. The glass tube outlet was placed at the bottom of the trapping solution, meaning the outlet lay 6 mm below the trapping solution surface. UV lamp current was set at 20 mA for all experiments. Flow rate through mass flow controller (MFC) 1 was set at 10 L min^{-1}, with 1 L min^{-1} being passed through the hygrometer

to measure [H_2O]. Flow rate through MFC 2 was set at 1 L min^{-1}. Mixing these two gas streams created a flow rate through the UV lamp of 10 L min^{-1}. This resulted in a 37 ms lamp residence time. Under these standard conditions, in the total flow [H_2O] was measured to be ~3.0×10^{17} molec. cm^{-3} and [*n*-nonane] was calculated to be ~1.0×10^{16} molec. cm^{-3}, using its partial volume, vapour pressure (408 Pa, 293 K)[S7] and the ideal gas law. The pump suction rate was set at 6.0 L min^{-1}, with gas intake through flow meters 1 and 2 set at 1.5 and 4.5 L min^{-1} respectively. This 1.5 L min^{-1} flow rate through the trapping solution ensured rapid but controllable bubbling. This resulted in a residence time of 92 ms and therefore total residence time 129 ms. Under these standard conditions, the initial gaseous [$HO_x^\bullet$] was calculated to be ~$6.8\pm1.0\times10^{11}$ (~28±4 ppbv) and therefore, [$^\bullet$OH] and [$HO_2^\bullet$] were each calculated to be ~$3.4\pm0.5\times10^{11}$ (~14±2 ppb). This was orders of magnitude lower than [*n*-nonane], ensuring nearly all $^\bullet$OH was converted into $RO_2^\bullet$. The sampling time was set between 10-100 min. After trapping completion, the trapping solution solvent was carefully removed *in vacuo* (30 ^{o}C, 100 mbar, ~10 min) and the residue was MS characterised. Alkanes do not absorb light at the wavelengths emitted by the UV lamp and therefore nonane degradation was assumed to be initiated exclusively by reaction with $^\bullet$OH[S8]. Therefore, nonane could be inputted through the UV lamp stream without compromising the experiment.

In the main paper, DEADANT (50 µM) was used for trapping over 10 min. In a supplementary reaction (Figure S31), CHANT (50 µM) was used for trapping over 10-100 min. For control experiments, the trap was omitted.

S5.6. Barton reaction

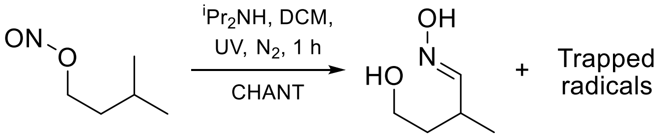

The procedure for trapping radicals in the Barton reaction was adapted from literature[S9]. Isopentyl nitrite (58.6 mg, 0.500 mmol, 1.0 eq.) was placed in a UV transparent polypropylene centrifuge tube and purged with N_2. DCM (30 mL) and diisopropylamine (202 mg, 2.00 mmol, 4.0 eq.) were added. If undertaking radical trapping, CHANT (32.2 mg, 0.100 mmol, 0.2 eq.) was added. For all reaction mixtures, an aliquot was removed (6 mL), and the remaining solution was irradiated using a UV lamp (100 W, 405 nm) for 1 h whilst stirring. An aliquot was then removed (6 mL). All aliquots had solvent removed *in vacuo* and were MS characterised.

S5.7. Hunsdiecker reaction

S5.7.1. Precursor synthesis

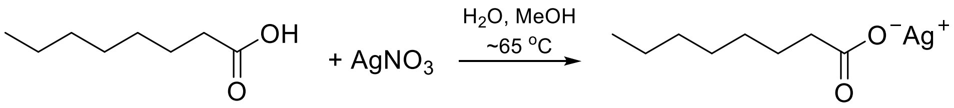

The procedure for silver octanoate synthesis was adapted from literature[S10]. Octanoic acid (288 mg, 2.0 mmol, 1.0 eq.) and $AgNO_3$ (340 mg, 2.0 mmol, 1.0 eq.) dissolved in MeOH (5.0 mL) and NaOH (2.0 M, 1.0 mL) were heated to reflux (~65 ^{o}C) to aid dissolution and allowed to cool. Precipitated silver octanoate was filtered and washed with water (5 mL) and MeOH (5 mL). Residual solvent was removed *in vacuo* overnight, yielding grey silver octanoate flakes (407 mg, 81%). Product was used in trapping reactions without characterisation or further purification.

S5.7.2. Trapping reaction

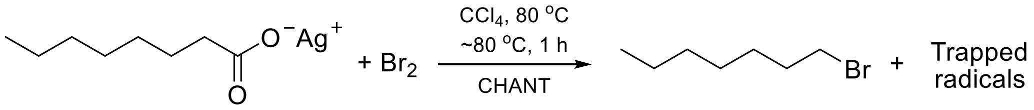

The procedure for trapping radicals in the Hunsdiecker reaction was adapted from literature[S11]. Silver octanoate (126 mg, 0.500 mmol, 1.0 eq.) was dissolved in CCl_4 (1.5 mL). If undertaking radical trapping, CHANT (32.2 mg, 0.100 mmol, 0.2 eq.) was added. This solution was then cooled in an ice bath (~0 °C) and Br_2 (2.0 M in CCl_4, 0.25 mL, 1.0 eq.) added dropwise over 15 min. For all reaction mixtures, an aliquot was removed (0.35 mL) and the remaining solution heated to reflux (~80 °C) for 1 h, whilst stirring. This was let cool and filtered before another aliquot was removed (0.35 mL). All aliquots had solvent removed *in vacuo* and were MS characterised.

S5.8. Decarboxylate aromatic iodination reaction

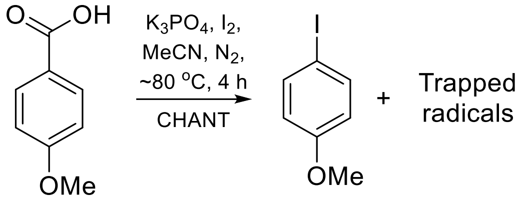

The procedure for trapping in a decarboxylate aromatic iodination reaction was adapted from literature[S12]. I_2 (508 mg, 2.00 mmol, 4.0 eq.) was placed under N_2 and *p*-anisic acid (76.1 mg, 0.500 mmol, 1.0 eq.), K_3PO_4 (106 mg, 0.500 mmol, 1.0 eq.) and MeCN (2.5 mL) were added. If undertaking radical trapping, CHANT (32.2 mg, 0.100 mmol, 0.2 eq.) was added. For all reaction mixtures, this solution was resealed and an aliquot removed (0.50 mL). The remaining solution was heated to reflux (~80 °C) and stirred for 4 h. This was let cool before another aliquot was removed (0.50 mL). All aliquots had solvent removed *in vacuo* and were MS characterised.

S5.9. Aqueous •OH-initiated degradation of methanol

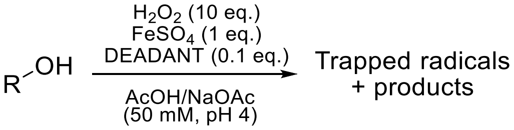

The procedure for trapping in aqueous •OH-initiated biochemical degradation was adapted from literature[S13-S15]. AcOH/NaOAc buffer (50 mM, pH 4, 580 µL) and methanol (10.0 mM in AcOH/NaOAc buffer, 1.0 mL, 10.0 µmol, 1.0 eq.) were mixed and stirred. $Fe_2SO_4{\cdot}7H_2O$ solution (500 mM in AcOH/NaOAc buffer, 20 µL, 10.0 µmol, 1.0 eq.) was added. If undertaking radical trapping, DEADANT solution (5.0 mM in AcOH/NaOAc buffer, 200 µL, 1.0 µmol, 0.10 eq.) was added but otherwise, AcOH/NaOAc buffer (200 µL) was added. For all reaction mixtures, H_2O_2 solution (500 mM in AcOH/NaOAc buffer, 200 µL, 100 µmol, 10 eq.) was added slowly over 5 min and the solution stirred overnight unsealed. This was MS characterised.

S5.10. Aqueous •OH-initiated degradation of thymine, glucose and ascorbic acid

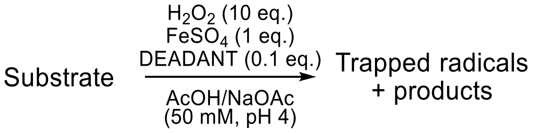

The procedure for trapping in aqueous •OH-initiated degradation was adapted from literature[S13-S15]. Substrate (1.26 mg, 10.0 µmol, 1.0 eq.) was dissolved in AcOH/NaOAc buffer (50 mM, pH 4, 1580 µL) with stirring. $Fe_2SO_4 \cdot 7H_2O$ solution (500 mM in AcOH/NaOAc buffer, 20 µL, 10.0 µmol, 1.0 eq.) was added and if undertaking radical trapping, DEADANT solution (5.0 mM in AcOH/NaOAc buffer, 200 µL, 1.0 µmol, 0.1 eq.) was also added. H_2O_2 solution (500 mM in AcOH/NaOAc buffer, 200 µL, 100 µmol, 10 eq.) was added slowly over 5 min and the solution was stirred overnight unsealed. This was then MS characterised.

S6. MS procedures for trapping reaction analysis

S6.1. Characterisation

A high resolution solariX XR FTMS (solariX) mass spectrometer (*m/z* ±0.0001 precision, >10^7 maximum resolution, mass accuracy 600 ppb (internal)) using positive ion mode electrospray ionisation (Pos ESI-MS) was used for MS characterisation of radical trapping samples, unless stated otherwise.

Dissolution solvent (1:1 CH_3CN:H_2O), MS solvent (0.1%/1:1 HCOOH/CH_3CN:H_2O), deuterated dissolution solvent (1:1 CH_3CN:D_2O) and deuterated MS solvent (0.1%/1:1 DCOOD/CH_3CN:D_2O) were prepared when required. Samples not in solution were dissolved in dissolution solvent (0.0-2.0 mL). An appropriate aliquot was then extracted from samples in solution using a Hamilton syringe and diluted with MS solvent to form a solution of ~10 μM trap concentration, assuming the number of moles of trap were equal before reaction and after reaction. Dilution of trapping reaction samples and control samples were scaled to ensure equivalent concentrations between samples, assuming no reaction had taken place. The *m/z* of the mass spectrometer was calibrated using observed and known peaks for sodium trifluoroacetate in MS solvent.

Standard direct injection was the first MS characterisation recorded for most samples. Parameters were initially optimised and then kept constant for all experiments, including *m/z* recording range of *m/z* 100-1000. MS solvent was injected (2 μL min^{-1}) and once signal stabilised, an average background spectrum was recorded (typically 16 scans). The same process was then performed for diluted sample and an average sample spectrum was recorded. The system was then flushed clean with MS solvent. D_2O exchange MS was performed similarly, however deuterated solvents were used instead. Samples were allowed to equilibrate in deuterated solvent for at least 1 h prior to MS characterisation. Tandem MS was also performed similarly to standard MS, but sometimes required parameter adjustments, such as ion acquisition time. Tandem MS involved using source CID isolation to isolate a peak of interest. Subsequent source CID fragmentation was used to fragment the peak. If source CID isolation could not obtain clean peak isolation, then additional in-cell SORI-CID was used to remove nearby offending peaks. Fragmentation was then similarly carried out as before, but utilising in-cell SORI-CID fragmentation instead of source CID fragmentation.

HPLC-MS used an Agilent-1200 HPLC instrument, equipped with a reverse phase column, connected to the mass spectrometer. Columns used were Eclipse Plus Phenyl-Hexyl 1.8 um, 4.6×50 mm reverse phase column, Atlantis C18 3 um, 4.6×150 mm and Waters Symmetry C18 3.5 μm, 4.6×75 mm, depending on the investigated system (S6.3). MS method parameters were optimised for HPLC-MS conditions and single sequential scans were recorded. HPLC combined 0.1%HCOOH/H_2O and 0.1%HCOOH/MeCN into a mobile phase injected at 300 μL min^{-1} or 500 μL min^{-1} for samples related to CHANT and DEADANT respectively. Solvent gradient varied from high H_2O to high MeCN content as required. During the run, column output was sometimes sent to waste, to prevent permanent contamination of the mass spectrometer by overly concentrated species.

S6.2. Analysis

Standard MS analysis involved either screening mass spectra for MS peaks corresponding to predicted structures (Peak Pick) or predicting structures corresponding to observed MS peaks (Formula Find). In both of these home-written MATLAB scripts, molecular formula

assignments were formulated on the accurate mass of pseudomolecular ions $[M+H]^+$, $[M+Na]^+$ or $[M+K]^+$.

In the Peak Pick programme, species were hypothesised and inputted into a Microsoft Excel table, along with their predicted *m/z* when protonated, sodiated and potassiated. Peak Pick then imported these *m/z* values and screened mass spectra for them. Observed peak m/z and intensity were then exported into the Microsoft Excel table. Additionally, Peak Pick outputted any peaks above a defined fraction of maximum peak intensity, which had not been inputted into the original Microsoft Excel table. This was to find any intense peaks which had not been hypothesised. Peak Pick was also adapted to include carbon content estimation.

The Formula Find programme used a reverse strategy to Peak Pick and aimed to find molecular formulae from observed peaks. Species could then be hypothesised for obtained molecular formulae.

In both methods, background spectra were subtracted from sample spectra during automated processing. Minor fluctuations in *m/z* accuracy required alignment of background and sample peaks with the same predicted *m/z* but different measured *m/z*, within set acceptance limits. Background peak intensities were then subtracted from sample peak intensities. If no background peak was observed within the accepted *m/z* range of the sample peak, intensity of the nearest background *m/z* value was subtracted instead. Both analysis methods used acceptance limits to prevent false positives. These included random *m/z* error (typically *m/z* <0.0000-0.0015), systematic *m/z* error (typically *m/z* -0.0006-0.0003) and minimum acceptable intensity. The Formula Find Method used additional acceptance limits to increase method speed. These were intensity of background peak compared to sample peak (typically <50%), *m/z* search range (e.g. *m/z* 100-500), species molecular formulae range (e.g., $C_{0-20}H_{0-100}N_{0-2}O_{0-10}$) and unsaturation range. Both methods outputted observed peak *m/z*, difference from predicted *m/z* and intensity. The Formula Find Method additionally outputted predicted *m/z* and molecular formulae. D_2O exchange MS and tandem MS analysis were usually performed using Peak Pick and Formula Find, respectively. HPLC-MS analysis was usually performed manually. Peak Pick and Formula Find programmes are available upon request.

S6.3. Parameters

MS characterisation was recorded using a solariX XR FTMS mass spectrometer in positive-ion mode ESI, unless stated otherwise. Mass spectra were recorded over an *m/z* range of *m/z* 100-1000. If multiple scans were used, these were averaged. Ion transfer time (TOF) was set to 1.0 ms. In general, ESI settings were as follows: drying gas flow = 4.0 L min^{-1}; nebulizer pressure: 2.0 bar; capillary voltage = 4500 V; spray shield voltage = -500 V; skimmer voltage = 15 V.

For standard MS (including D_2O exchange), other settings used were: injection speed = 2 µL min^{-1}; scans = 16; ion accumulation time = 0.2 s; drying gas temperature = 160 °C.

For tandem MS, other settings used were: scans = 16-64; ion accumulation time = 0.2-5 s; drying gas temperature = 160 °C; quadrupole MS/MS CID energy = 0-20 V, and in-source collision energy = 0-20 V. Number of scans and amount of ion accumulation time was set depending on the intensity of the desired MS peak. Quadrupole MS/MS CID energy and in-source collision energy were set to cause an appropriate amount of fragmentation.

For HPLC-MS, other settings used were: injection speed = 300 or 500 µL min^{-1} for samples relating to CHANT and DEADANT respectively; scans = single sequential; ion accumulation time = 0.2 s; drying gas temperature = 180 °C. HPLC conditions used for HPLC-MS are shown

for samples related to CHANT trapping (Table S1) and samples related to DEADANT trapping (Table S2).

Table S1: HPLC conditions of sample analysis from α-pinene ozonolysis and •OH-initiated *n*-nonane degradation when bubbled through CHANT solution. Column = Eclipse Plus Phenyl-Hexyl. Injection speed = 300 uL min^{-1}.

Time / m	0.1%HCOOH/ H_2O / %	0.1%HCOOH/ MeCN / %	MS or waste
0.0	95	5	MS
5.0	95	5	MS
10.0	20	80	MS
13.5	↓	↓	Waste
14.0	↓	↓	MS
25.0	5	95	MS
35.0	5	95	MS
39.0	95	5	MS
40.0	95	5	MS

Table S2: HPLC conditions of sample analysis from •OH-initiated *n*-nonane degradation when bubbled through DEADANT solution. Column = Atlantis. Injection speed = 500 uL min^{-1}.

Time / m	0.1%HCOOH/ H_2O / %	0.1%HCOOH/ MeCN / %	MS or waste
0.0	100	0	MS
1.0	↓	↓	Waste
5.0	100	0	Waste
15.0	90	10	MS
25.0	70	30	MS
30.0	30	70	MS
31.0	5	95	MS
35.0	5	95	MS
39.0	100	0	MS
40.0	100	0	MS

Table S3: HPLC conditions of sample analysis from radical decarboxylative aromatic iodination. Column = Waters Symmetry. Injection speed = 300 μL min^{-1}.

Time / m	0.1%HCOOH/ H_2O / %	0.1%HCOOH/ MeCN / %
0.0	95	5
1.0	95	5
2.0	50	50
8.0	5	95
22.0	5	95
24.0	95	5
25.0	95	5

S7. Kinetic modelling of nonane oxidation

S7.1. Gas phase modelling

Atmospheric reactions of *n*-nonane were imported into the Kintecus chemical simulation programme[S16] from the MCM[S17] and truncated to remove late stage pathways (Table S20). Potential wall reactions were ignored and simulation conditions were set at standard temperature (293 K) and pressure. Rate constants are calculated using the Arrhenius equation $k = Ae^{-\frac{E_a}{RT}}$, where *A* is the pre-exponential factor, E_a is activation energy, *R* is the universal gas constant (8.314 J K^{-1} mol^{-1}) and *T* is absolute temperature (K). Key Kintecus parameters: E_a units = K; concentration units = molec. cm^{-3}; temperature = 293 K; accuracy = 10^{-10}.

Simulation parameters were set to emulate experimental conditions immediately prior to bubbling through the trapping solution. Simulation time was set as calculated residence time (129 ms). As measured or calculated, initial [$^{\bullet}OH$], [$HO_2^{\bullet}$] and [*n*-nonane] were set as $3.4\pm0.5\times10^{11}$ molec. cm^{-3}, $3.4\pm0.5\times10^{11}$ molec. cm^{-3} and 1.0×10^{16} molec. cm^{-3} respectively. Simulation results yielded final gaseous [$C_9H_{19}O_2^{\bullet}$] and [$C_9H_{19}O^{\bullet}$] to be $1.73\pm0.14\times10^{11}$ molec. cm^{-3} and $2.1\pm0.3\times10^{3}$ molec. cm^{-3} respectively, allowing detection limits to be estimated (Figure S32).

S7.2. Modelling reactions in the trapping solution

Peroxyl radicals ($RO_2^{\bullet}$) are much less reactive than the $R^{\bullet}$ and $RO^{\bullet}$ radicals formed in the nonane oxidation. The rate of the reaction with the trap (S1) can be estimated as *ca.* 1 L mol^{-1} s^{-1} [S18]. These radicals therefore accumulate in the trapping solution faster than they react with the trap. The main other reactions they undergo are self-reactions S2-S4.

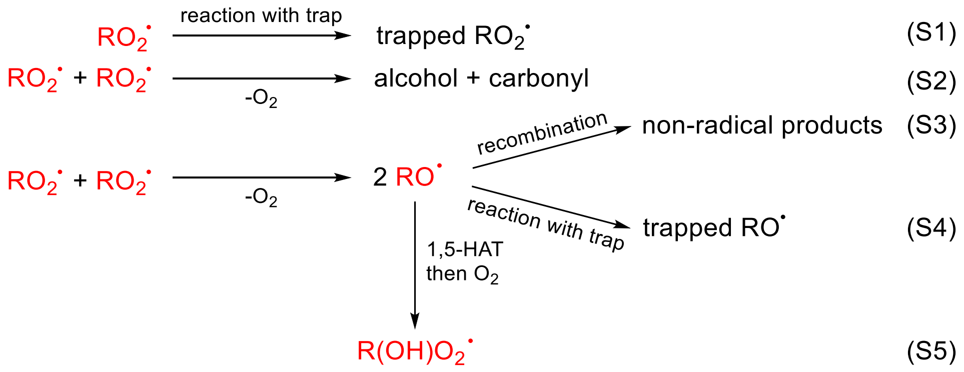

Reaction S2 (Russell mechanism) only affects primary and secondary $RO_2^{\bullet}$ radicals. The rate constant for this reaction is *ca.* 10^7 L mol^{-1} s^{-1} [S19-S21]. Alternatively, $RO_2^{\bullet}$ radicals self-react to form two $RO^{\bullet}$ radicals. The rate constant for this reaction is 10^4-10^5 L mol^{-1} s^{-1} [S22]. Reaction S3 can then proceed as an in-cage recombination or after the radicals have escaped from the solvent cage. Reaction S4 is the trapping of the $RO^{\bullet}$ radical with the rate constant of *ca.* 10^8-10^9 L mol^{-1} s^{-1} [S23]. A competing process for nonyl-oxyl radicals is intramolecular 1,5-hydrogen atom transfer (*ca.* 10^7 s^{-1})[S24] which, after reaction with oxygen, yields $R(OH)O_2^{\bullet}$ radicals (reaction S5). These reactions were imported into Kintecus programme[S16]. The overall process was modelled by adding $RO_2^{\bullet}$ radicals at experimental concentration (1.73×10^{11} molec. cm^{-3}), flow rate (1.5 slm) and accumulation time (10 min). The simulation was then run for a further 50 min (Figure S11).

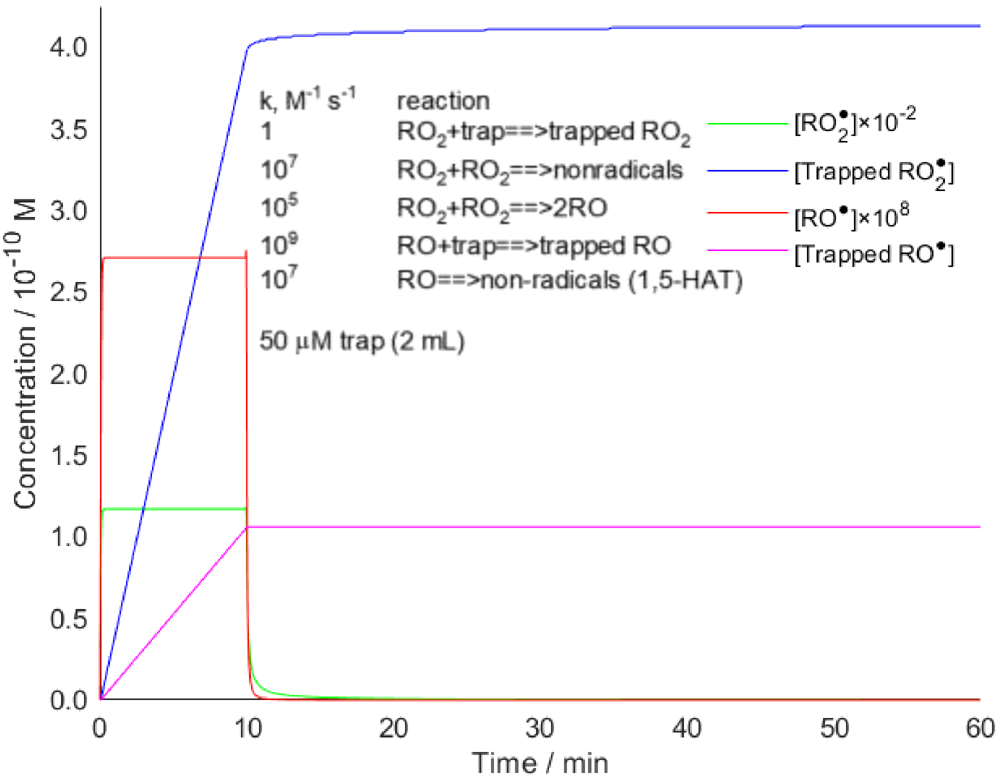

Figure S11. Kinetic modelling of $RO_2^•$ reactions in the trapping solution.

The simulation predicts end concentrations of trapped $RO_2^•$ and $RO^•$ radicals as $4×10^{-10}$ and $1×10^{-10}$ M, respectively. However, control experiments with $RO_2^•$ (nonane peroxyl) radicals prepared by a different route, showed significantly higher MS intensity for the direct injection of the trapped $RO_2^•$ as compared to the HPLC-MS analysis of the same mixture (the intensity was relative to trapped $RO^•$ in both cases). This suggests that a significant proportion of trapped $RO_2^•$ radicals degraded in the HPLC column preventing their detection. The concentration of trapped radicals can be increased by increasing the concentration of trap, however free TEMPO content in the purified trap must be sufficiently low to prevent non-radical degradation of $RO_2^•$ radicals (TEMPO reacts with $RO_2^•$ radicals to yield non-radical products with a rate constant *ca.* 10^8 L mol^{-1} s^{-1})[S25].

Experimentally, trapped $RO^•$ radicals were detected at $1×10^{-10}$ M concentration with a signal-to-noise ratio *ca.* 200 (Figure 7B in the main text). A similar simulation shows that trapping a gas stream with $1.5×10^9$ molec. cm^{-3} concentration of $RO_2^•$ will yield signal-to-noise ratio for trapped $RO^•$ radical of *ca.* 2 thus allowing us to estimate a detection limit.

A simulation involving trapping $RO^•$ radicals from the gas stream at their predicted concentration of $2.1×10^3$ molec. cm^{-3}, gives the end concentration of trapped $RO^•$ as $1.3×10^{-16}$ M, six orders of magnitude lower than that formed due to $RO_2^•$ self-reaction. We therefore conclude that nearly all trapped $RO^•$ is hence formed in the $RO_2^•$ self-reaction rather than absorbed from the gas phase.

S8. Additional trapping reaction schemes and results

S8.1. General

Liquid phase trapping involved including trap into a procedure for a liquid phase radical reaction sourced from literature. Gas phase trapping usually involved bubbling a gaseous radical reaction stream through a solution containing trap, where a procedure for the gas stream was sourced from literature.

For the five reactions discussed in detail in the main paper, some additional reaction schemes and results are given: thiol-ene addition (S8.2); Hofmann-Löffler-Freytag (HLF) reaction (S8.3); cyclohexene and α-pinene ozonolysis (S8.4) and $^\bullet$OH-initiated *n*-nonane degradation (S8.5). For the reactions principally discussed in this supplementary information, reaction schemes and results are shown: Barton reaction (S8.6); Hunsdiecker reaction (S8.7); Perry reaction (S8.8) and aqueous $^\bullet$OH-initiated degradation of methanol (S8.9), thymine (S8.10), glucose (S8.11) and ascorbic acid (S8.12).

A radical $R^\bullet$ can react with radical traps in several ways. $R^\bullet$ trapped through normal radical trapping was dubbed R−ART, since $R^\bullet$ replaced cleaved TEMPO. ART (allyl radical trap) denotes the trap residue, so for instance R−ART represents a trapped $R^\bullet$ radical (e.g., −ART is $-CH_2C(CONHC_6H_{11})=CH_2$ for CHANT). This type of trapping could theoretically occur with any radical. $R^\bullet$ trapped through recombination trapping with TEMPO was dubbed R−TEMPO. This reaction would not occur for heteroatom-centred radicals, such as nitrogen-centred, oxygen-centred or sulfur-centred radicals, due to the weak bond formed between the radical and TEMPO. Finally, hydrogen atom abstraction (HAA) from allylic C−H in CHANT/DEADANT to form an allylic carbon-centred radical and subsequent recombination trapping with $R^\bullet$ was dubbed R−CHANT or R−DEADANT, since TEMPO was not cleaved. It was feared that HAA from allylic C−H of the traps could cause significant side reactions. However, radical trapping experiments indicated that this reaction did not occur significantly. This is shown in radical trapping of thiol-ene addition (S8.2) and the HLF reactions (S8.3). R−ART could only be formed by $R^\bullet$, whilst R−TEMPO and R−CHANT/DEADANT could additionally be formed through non-radical side reactions. All species were searched for as protonated, sodiated and potassiated MS adducts, however only significantly observed MS peaks are shown. Peaks corresponding to CHANT-trapped radicals and DEADANT-trapped radicals were usually most intensely observed as MS adducts $[R{-}ART{+}Na]^+$ and $[R{-}ART{+}H]^+$, respectively, depending on the ionisation efficiency of R. Peaks corresponding to R−TEMPO and R−CHANT/DEADANT were usually most intensely observed as MS adducts $[R{-}TEMPO{+}H]^+$ and $[R{-}CHANT/DEADANT{+}H]^+$.

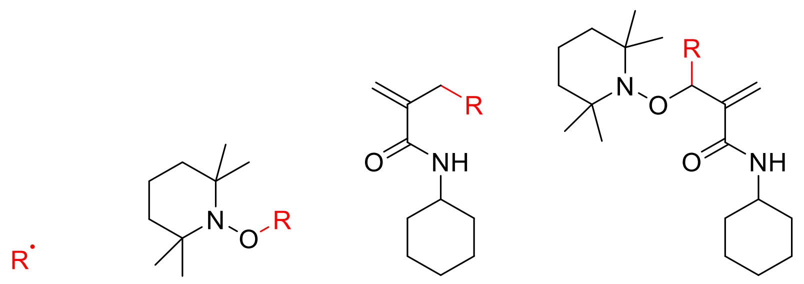

Reactive radical R R-TEMPO R-ART R-CHANT

S8.2. Thiol-ene addition

S8.2.1. MS results

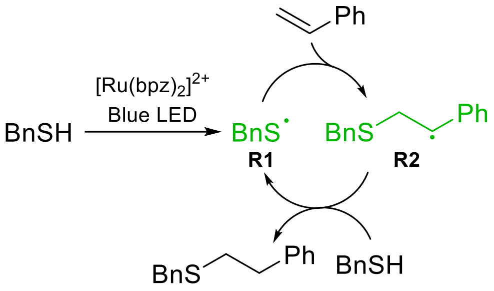

Figure S12: Mechanism of photoinitiated thiol-ene addition. Bn = CH_2Ph. Trapped radicals are shown in green.

Table S4: Species identified from radical trapping of photoinitiated thiol-ene addition, using MS for characterisation (S5.2). Systematic *m/z* error = -0.0003; random error *m/z* = ±0.0002; 100% intensity = 2.27x10^9 absolute count. ART denotes the trap residue, so R−ART represents a trapped R• radical i.e., −ART is $-CH_2C(CONHC_6H_{11})=CH_2$ for CHANT).

Species		Predicted *m/z*	Intensity relative to CHANT standard / % Trapless control	Pre-initiation	Post-trapping
Trap	$[CHANT+H]^+$	323.2698	0.078	70.5	82.5
Product	$[Thioether+Na]^+$	251.0870	0.091	0	0.090
Trapped radicals	$[R1-ART+Na]^+$	312.1398	0	0.025	0.490
	$[R1-CHANT+H]^+$	445.2889	0	0	0
	$[R2-ART+Na]^+$	416.2024	0	0	0.090
	$[R2-TEMPO+H]^+$	384.2361	0	0	2.01
	$[R2-CHANT+H]^+$	549.3515	0	0	0

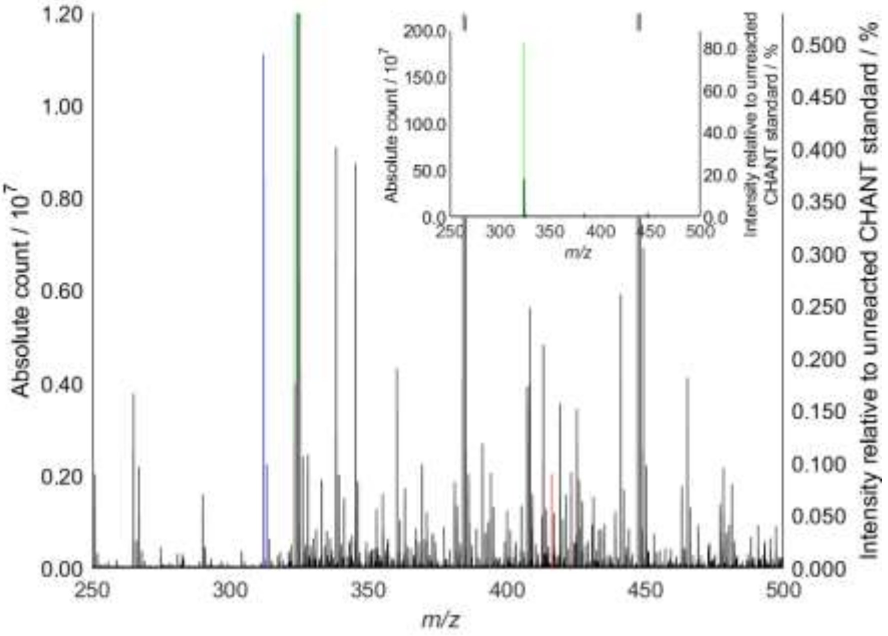

Figure S13: Background-corrected mass spectrum from post-trapping of thiol-ene addition (S5.2), showing peaks corresponding to unreacted CHANT (green) and trapped radicals R1−ART (blue) and R2−ART (red).

MS peaks corresponding to R1−ART, R2−ART and R2−TEMPO were observed exclusively or with much greater intensity post-trapping. Peaks corresponding to R1−TEMPO were not observed, as expected. Peaks corresponding to R1−CHANT and R2−CHANT were not observed, indicating side reactions involving hydrogen atom abstraction from allylic C−H in CHANT did not occur significantly.

S8.2.2. Analysis of relative steady-state concentrations of R1 and R2

The steady-state concentration of radicals R1 and R2 in the reaction mixture is determined by the ratio of rate constants k_2/k_3 and k_4/k_5 (scheme below).

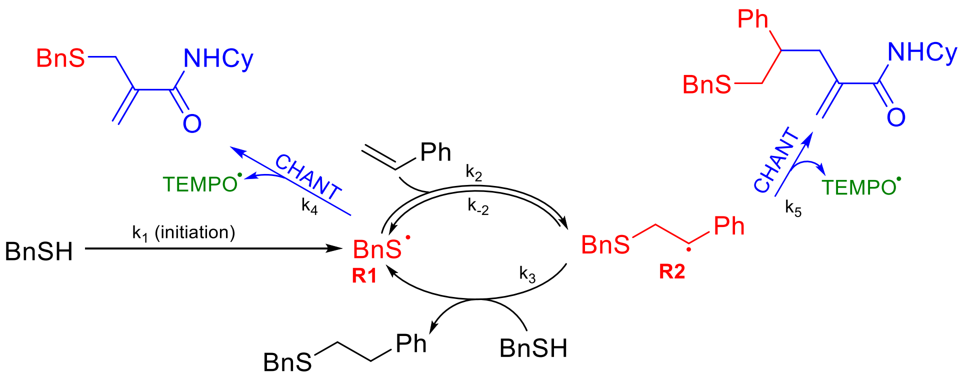

This is because the initiation step (k_1) is much slower than the propagation steps and hence it does not appreciably influence the concentration of R1. Addition of thiyl radicals to alkenes is in principle reversible, and the rate of the reverse reaction (k_{-2}) needs to be taken into consideration. For aliphatic thiyl radicals and terminal alkenes, however, the rate of addition k_2 is significantly faster than the reverse process (k_{-2}), and therefore this reaction can be considered essentially irreversible under reaction conditions[S26-S29]. As $k_4 >> k_5$ [S30, S31] and the intensities of trapped R1 and R2 are similar, we conclude that the steady-state concentration of R2 is higher than that of R1.

S8.3. Hofmann-Löffler Freytag (HLF) reaction

S8.3.1. MS results

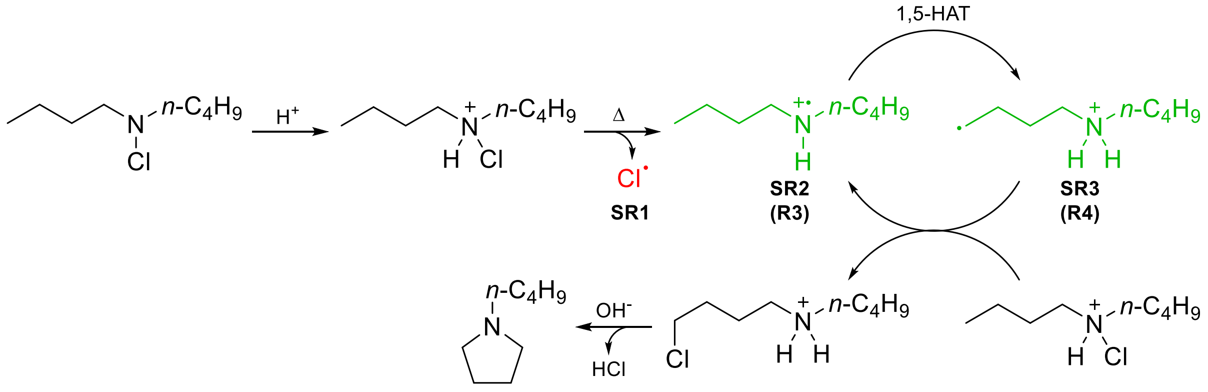

Figure S14: Widely accepted mechanism of the HLF reaction for *N*-chlorodibutylamine, first proposed by Corey *et al.*[S32]. Radicals detected by trapping are shown in green whilst the presence of others (red) could not be confirmed. SR2 = R3, SR3 = R4.

Table S5: Species identified from radical trapping of the HLF reaction, using MS for characterisation (S5.3). Cl containing species are shown with ^{35}Cl only. Systematic *m/z* error = 0.0000; random error *m/z* = ±0.0009; 100% intensity = 2.14x10^9 absolute count. ART denotes the trap residue, so R−ART represents a trapped R$^•$ radical i.e., −ART is $-CH_2C(CONHC_6H_{11})=CH_2$ for CHANT).

	Species	Predicted *m/z*	Intensity relative to unreacted CHANT pre-trapping reaction / %: Trapless control	Pre-initiation	Post-trapping
Trap	[CHANT+H]$^+$	323.2698	0	100	47.2
Product	[*N*-Butylpyridine+H]$^+$	128.1439	0.003	0	0.157
Trapped radicals	[SR1−ART+H]$^+$	202.0999	0	0	0
	[SR1−ART+Na]$^+$	224.0818	0	0	0
	[SR1−CHANT+H]$^+$	357.2309	0	0	0.287
	[R3/R4−ART+H]$^+$	295.2749	0	0	23.8
	[R4−TEMPO+H]$^+$	285.2906	0.001	0	0.008
	[R3/R4−CHANT+H]$^+$	450.4059	0	0	0.021
Side products	[CHANT+HCl+H]$^+$	359.2465	0.002	0	0
	[CHANT+HOCl+H]$^+$	375.2414	0.009	0	0.312

A MS peak corresponding to R3/R4−ART was observed exclusively post−trapping. This peak was clearly visible in the mass spectrum and was also easily observed using a lower resolution compact mass spectrometer (Figure S15). A peak corresponding to R4−TEMPO was likewise observed with significantly greater intensity post−trapping reaction compared to other samples. No peak corresponding to SR1−ART was observed. This was likely due to this SR1 only being produced during initiation, causing low [SR1] and hence, low [SR1−ART]. Peaks corresponding to R3/R4−CHANT were observed with relatively low intensity compared to peaks corresponding to R3/R4−ART, indicating side reactions involving hydrogen atom abstraction (HAA) from allylic C−H in CHANT did not occur significantly. Peaks corresponding to SR1−CHANT were observed, likely due to Cl$^-$ reaction with CHANT. Non-observation of SR1−ART corresponding peaks therefore further indicated that CHANT−trapped radicals were only produced through radical reactions.

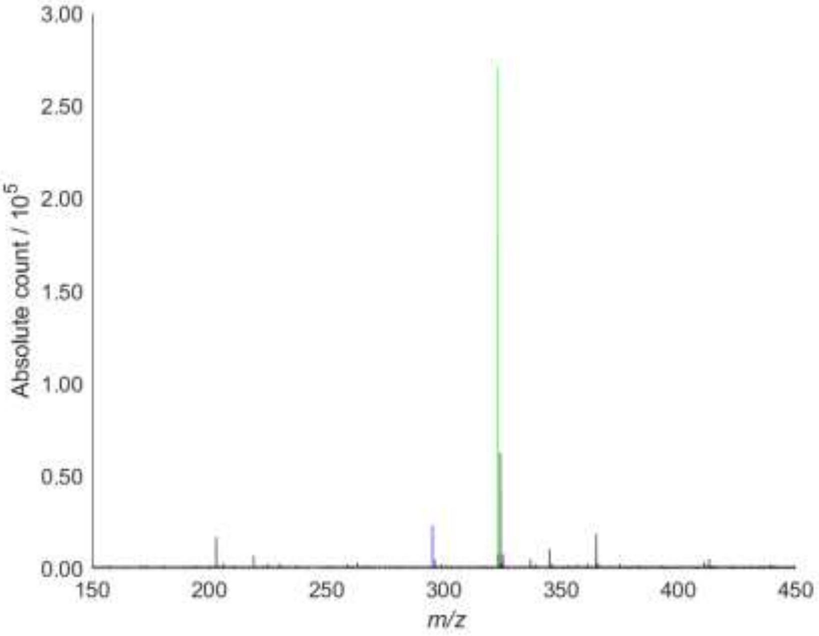

Figure S15: Mass spectrum from post-trapping of the HLF reaction (S5.3), showing peaks corresponding to unreacted CHANT (*m/z* 323.270, green) and R3/R4−ART (*m/z* 295.275, blue), recorded using lower resolution compact mass spectrometer.

S8.3.2. D_2O exchange

Table S6: D_2O exchange performed upon sample from post-trapping of the HLF reaction, using MS for characterisation (S5.3). Systematic *m/z* error = 0.0002; random error *m/z* = ±0.0008. ART denotes the trap residue, so R−ART represents a trapped R$^\bullet$ radical i.e., −ART is $-CH_2C(CONHC_6H_{11})=CH_2$ for CHANT).

Identified species		Relative intensity / % 0D	1D	2D	3D	Implies radical
Trap	$[CHANT+D]^+$	0	1.51	**98.49**	0	-
Products	[*N*-Butylpyridine+D]$^+$	10.34	89.66	0	0	-
Trapped radicals	[R3/R4−ART+D]$^+$	0.06	3.23	**96.71**	0[a]	R3
	[R3/R4−TEMPO+D]$^+$	0	0	**100**	0	R4

[a]Peak possibly present with low intensity but dominated by peak of different *m/z*.

S8.3.3. Tandem MS

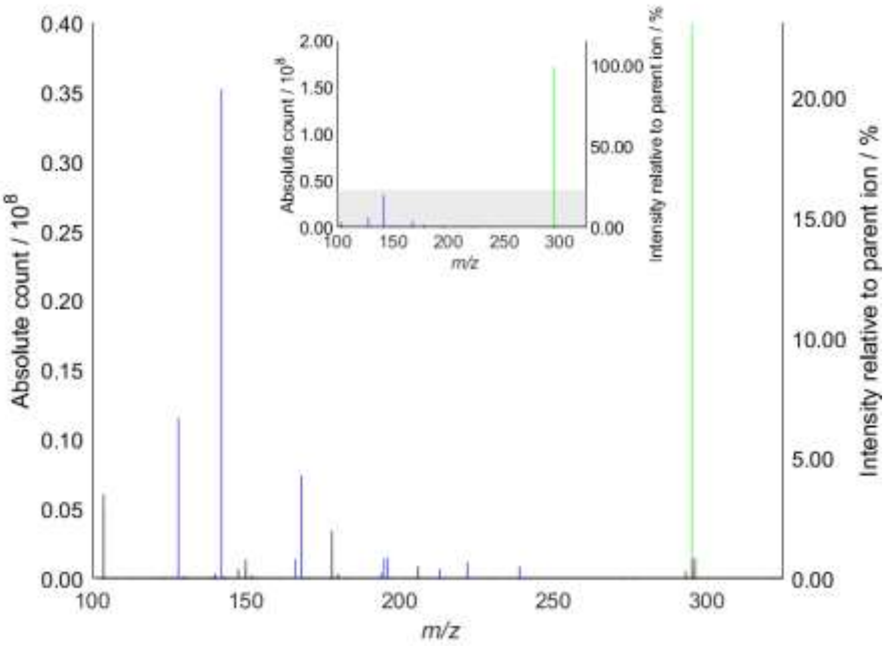

Figure S16: Tandem mass spectrum performed upon the peak corresponding to [R3/R4−ART+H]$^+$ (*m/z* 295.275, green) from post-trapping of the HLF reaction (S5.3). Structures could be assigned to most fragments (Table S7, blue).

Table S7: Observed fragment *m/z* and suggested structures identified from tandem MS upon peak corresponding to [R3/R4−ART+H]$^+$ (*m/z* 295.275) from post-trapping of the HLF reaction (S5.3). Fragments of *m/z* 222.185 and 194.154 could only be assigned to R4−ART. ART denotes the trap residue, so R−ART represents a trapped R$^\bullet$ radical i.e., −ART is $-CH_2C(CONHC_6H_{11})=CH_2$ for CHANT).

Identified species		Predicted *m/z*	Observed *m/z*	Relative intensity / %	Implies radical
[R3−ART+H]$^+$	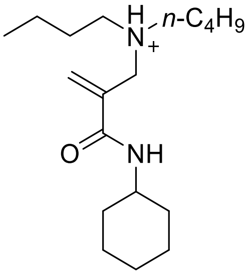	295.2749	295.2743	100	R3
[R4−ART+H]$^+$	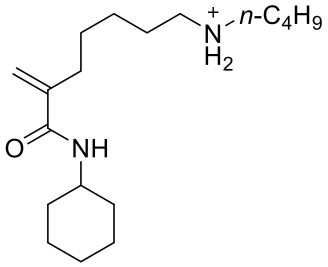	295.2749	295.2743	100	R4
Fragments	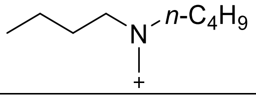	142.1596	142.1590	20.4	R3/R4

	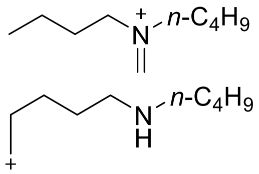				
	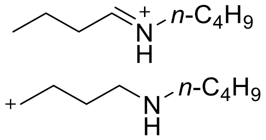	128.1439	128.1433	6.70	R3/R4
	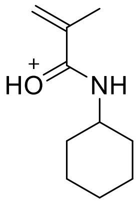	168.1388	168.1382	4.31	R3/R4
	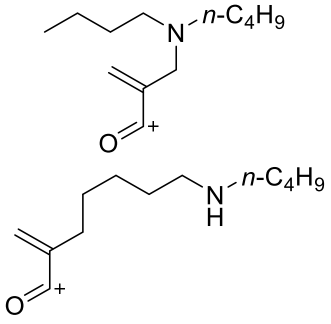	196.1701	196.1695	0.882	R3/R4
	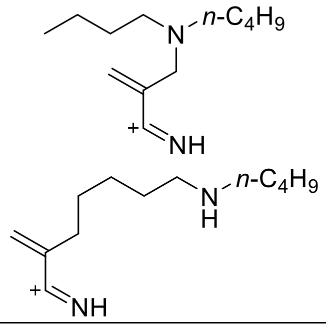	195.1861	195.1855	0.864	R3/R4
	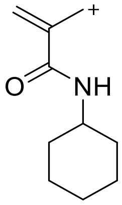	166.1232	166.1226	0.836	R3/R4
	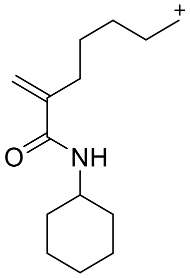	222.1858	222.1852	0.709	R4

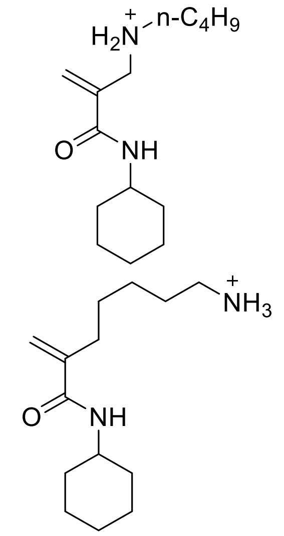	239.2123	239.2117	0.537	R3/R4
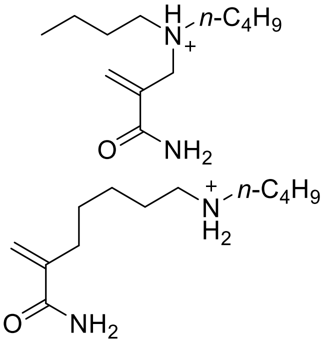	213.1967	213.1961	0.419	R3/R4
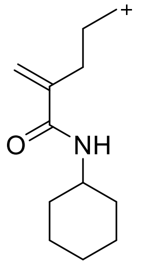	194.1545	194.1539	0.257	R4

S8.4. Alkene ozonolysis

S8.4.1. CHANT ozonolysis

Table S8: CHANT ozonolysis products identified in radical trapping of alkene ozonolysis, using MS for characterisation (S5.4). Systematic *m/z* error = -0.0005; random error *m/z* = ±0.0003; 100% intensity = 3.08×10^9 absolute count.

Species	Predicted *m/z*	Intensity relative to unreacted CHANT standard / %		
		No alkene	Cyclohexene	α-Pinene
$[CHANT+H]^+$	323.2698	0.164	48.4	18.3
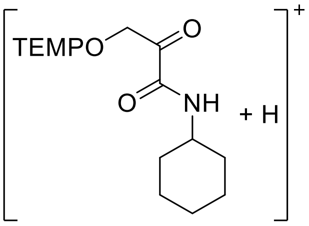	325.2491	1.86	0	0.010
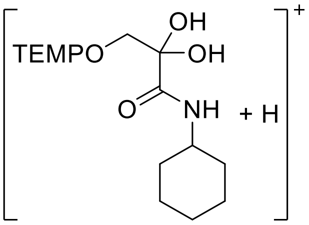	343.2597	6.52	0	0.036
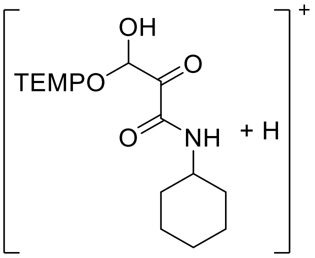	341.2440	0.11	0	0

S8.4.2. Cyclohexene ozonolysis

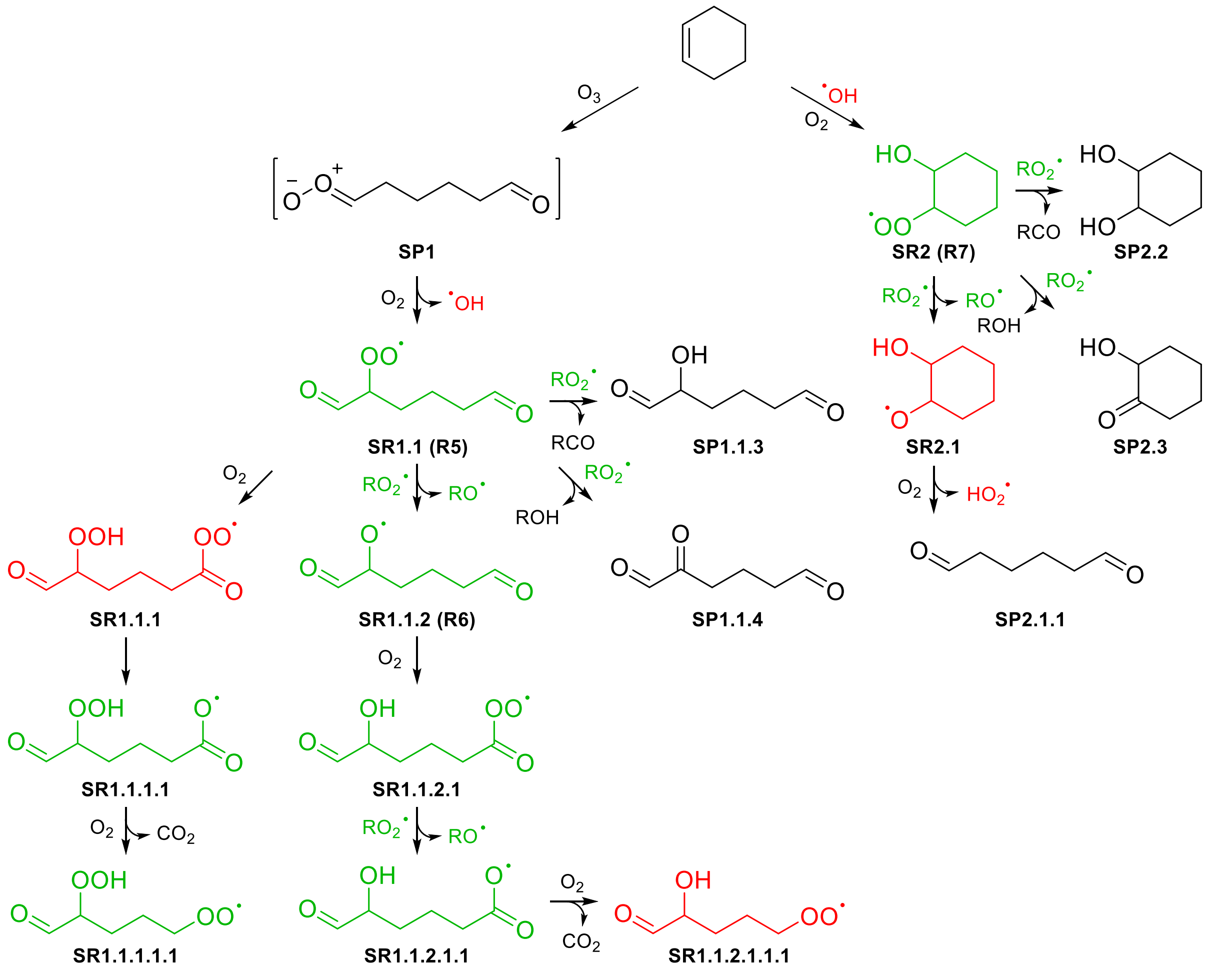

Figure S17: Non-comprehensive hypothesised mechanism of cyclohexene ozonolysis. Radicals detected by trapping (or their isomers) are shown in green whilst the presence of others (red) could not be confirmed. O_2 release is not shown. RCO represents carbonyl species. SR1.1 = R5, SR1.1.2 = R6, SR2 = R7.

Table S9: Species identified from radical trapping of cyclohexene ozonolysis, using MS for characterisation (S5.4). Systematic *m/z* error = -0.0005; random error *m/z* = ±0.0005; 100% intensity = 3.08×10^9 absolute count. ART denotes the trap residue, so R−ART represents a trapped R• radical i.e., −ART is $-CH_2C(CONHC_6H_{11})=CH_2$ for CHANT).

Species		Predicted *m/z*	Intensity relative to unreacted CHANT standard / %		
			Trapless control	Standard	Trapping reaction
Trap	$[CHANT+H]^+$	323.2698	0	100	48.4
Trapped radicals	$[R5-ART+Na]^+$	334.1630[a]	0	0	0.031
	$[SR1.1.1-ART+Na]^+$	366.1529	0	0	0
	$[SR1.1.1.1-ART+Na]^+$	350.1580[a]	0	0	0.004
	$[SR1.1.1.1.1-ART+Na]^+$	338.1579	0	0	0.005
	$[R6-ART+Na]^{+b}$	318.1681	0	0	0.032
	$[SR1.1.2.1-ART+Na]^+$	350.1580[a]	0	0	0.004
	$[SR1.1.2.1.1-ART+Na]^+$	334.1630[a]	0	0	0.031
	$[SR1.1.2.1.1.1-ART+Na]^+$	322.1630	0	0	0
	$[R7-ART+Na]^+$	320.1838	0	0	0.009
	$[SR2.1-ART+Na]^{+b}$	304.1889	0	0	0

	[SP1.1.3+Na]$^+$	153.0528[a]	0.037	0	0.095
	[SP1.1.4+Na]$^+$	151.0371	0.002	0	0.002
Products	[SP2.1.1+Na]$^+$	137.0579[a]	0.045	0	0.127
	[SP2.2+Na]$^+$	139.0735	0	0	0
	[SP2.3+Na]$^+$	137.0579[a]	0.045	0	0.127

[a]Other table entries have predicted species with identical *m/z*. [b]RO• radicals R6 and SR2.1 could have at least partially formed from the self-reaction of the parent RO_2• radicals in the trapping solution, see section S7.2).

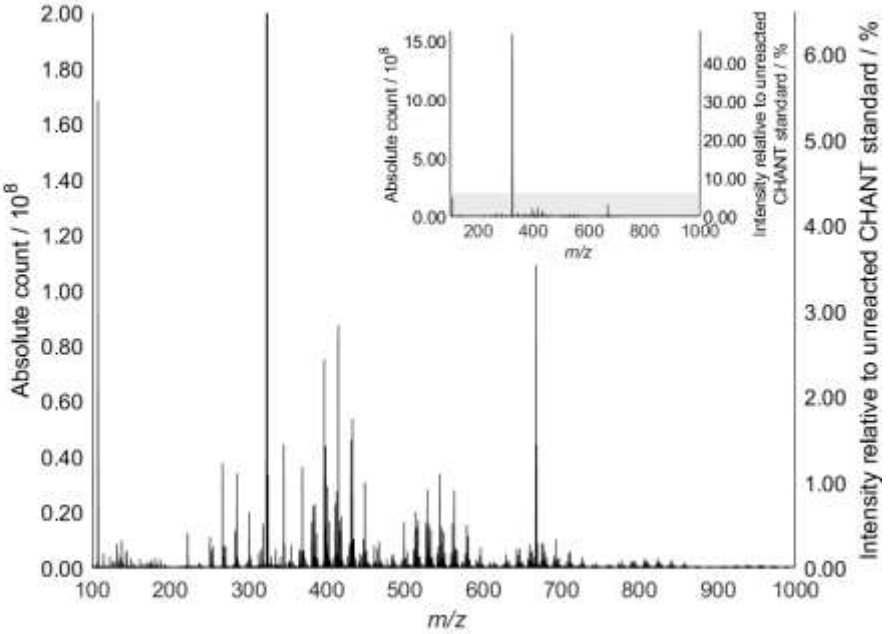

Figure S18: Background corrected mass spectrum from trapping reaction of cyclohexene ozonolysis (S5.4). 100% intensity = 3.08×10^9 absolute count.

S8.4.3. α-Pinene ozonolysis

S8.4.3.1. Reaction schemes

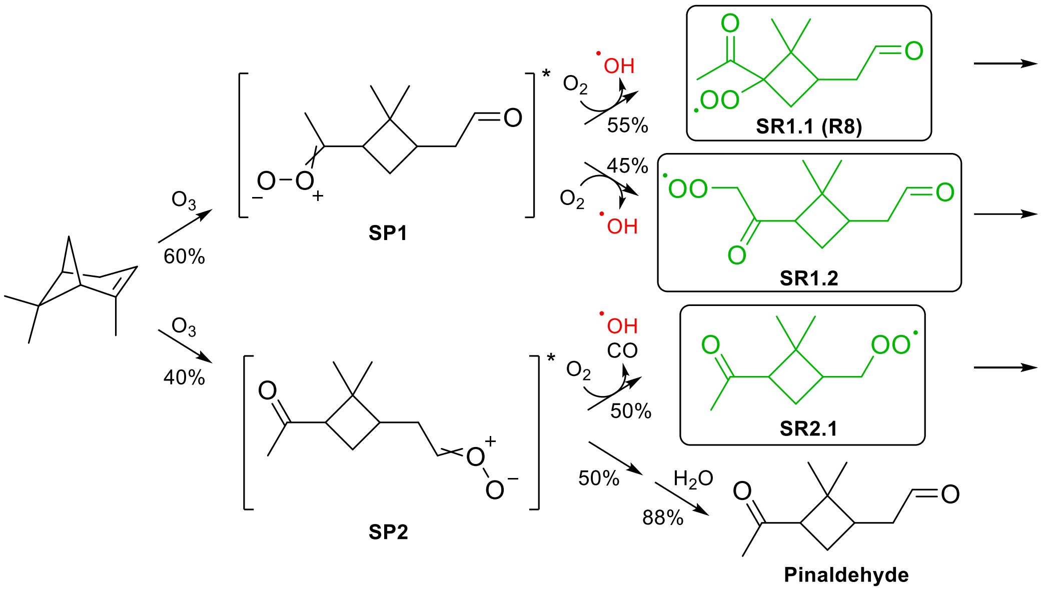

Figure S19: α-Pinene reaction with ozone leading to $RO_2^{\bullet}$ formation in α-pinene ozonolysis mechanism. Radicals detected by trapping (or their isomers) are shown in green whilst the presence of others (red) could not be confirmed. Structures and pathway probabilities were obtained from the MCM[S17]. SR1.1 = R8.

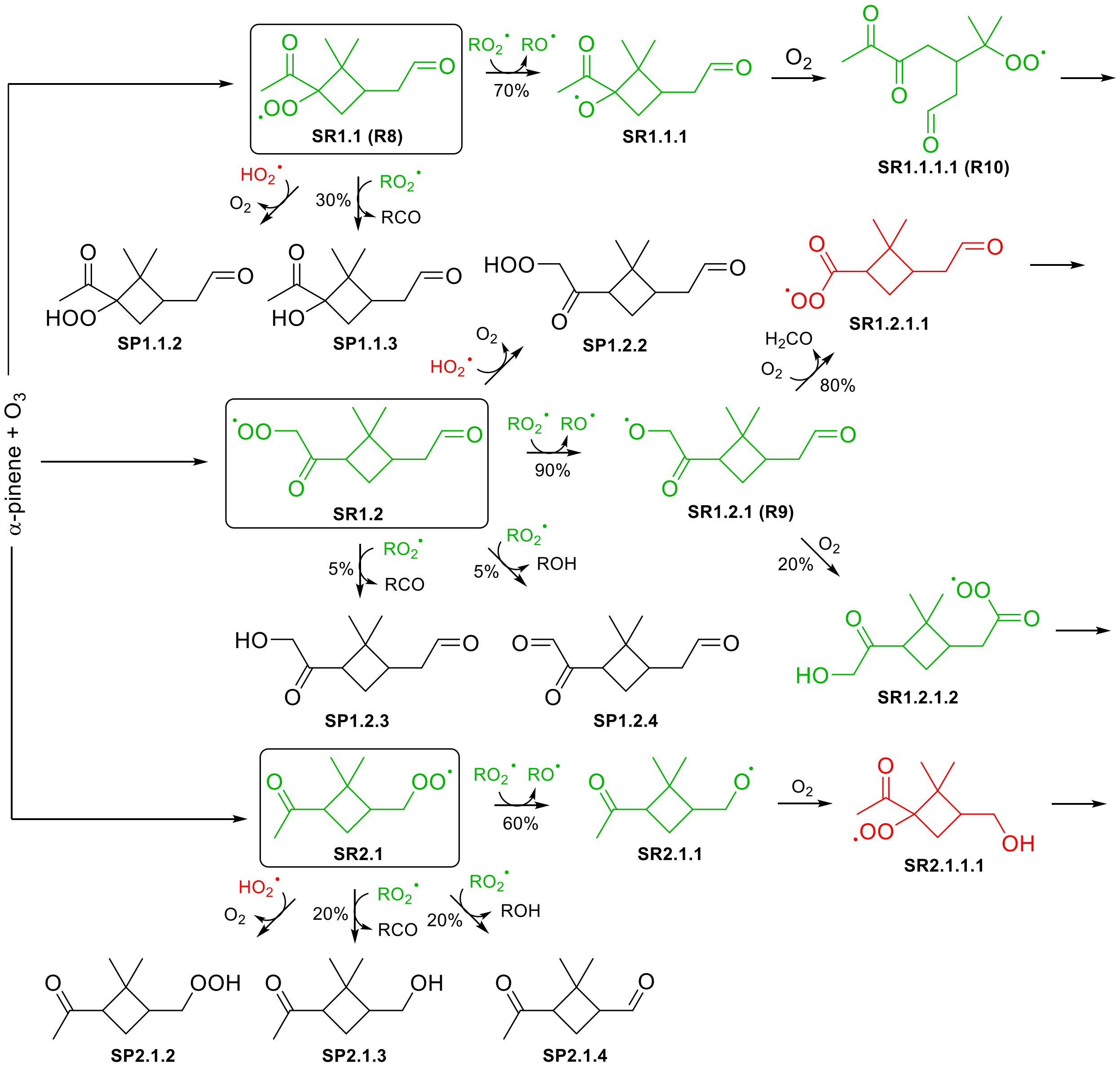

Figure S20: $RO_2^•$ degradation leading to $RO^•$ and further $RO_2^•$ in α-pinene ozonolysis mechanism. Radicals detected by trapping (or their isomers) are shown in green whilst the presence of others (red) could not be confirmed. O_2 release is not shown. RCO represent carbonyl species. Structures and pathway probabilities were obtained from the MCM [S17]. SR1.1 = R8, SR1.2.1 = R9, SR1.1.1.1 = R10.

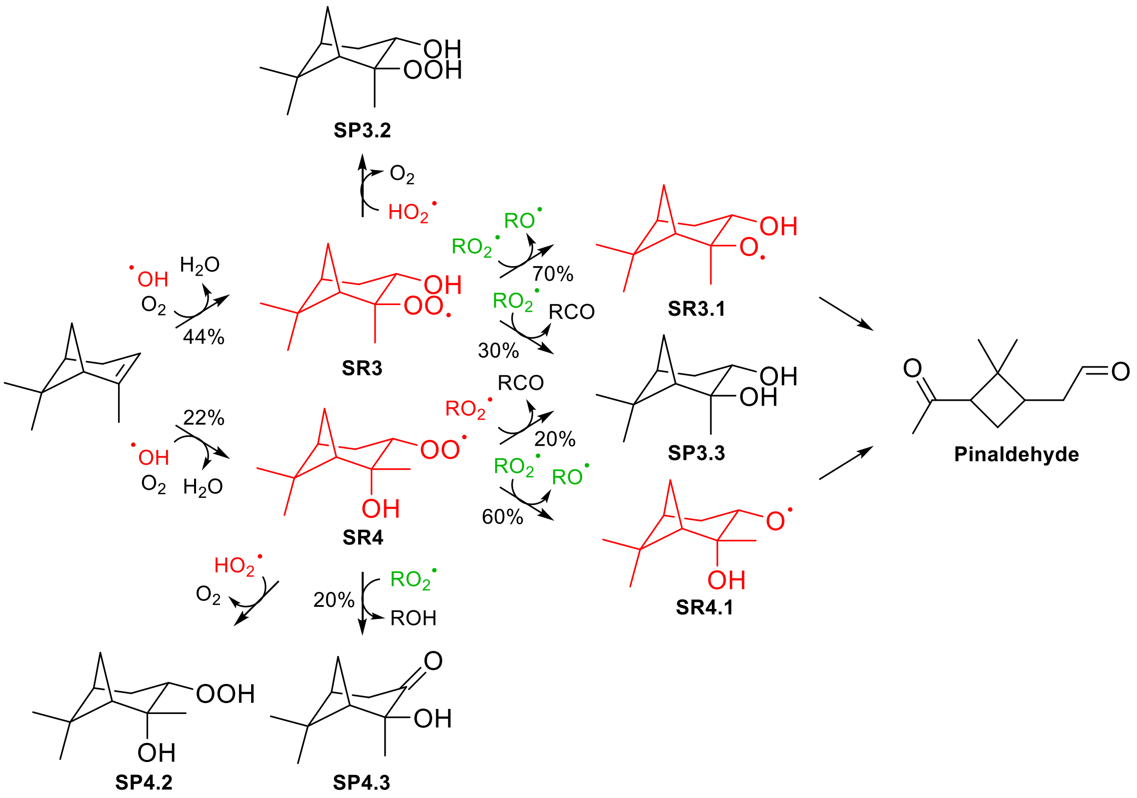

Figure S21: •OH addition to α-pinene leading to β-hydroxy $RO_2^{\bullet}$ and RO• in α-pinene ozonolysis mechanism. Radicals detected by trapping are shown in green whilst the presence of others (red) could not be confirmed. (red). O_2 release is not shown. RCO represent carbonyl species. Structures and pathway probabilities were obtained from the MCM and other sources [S17, S33-S35].

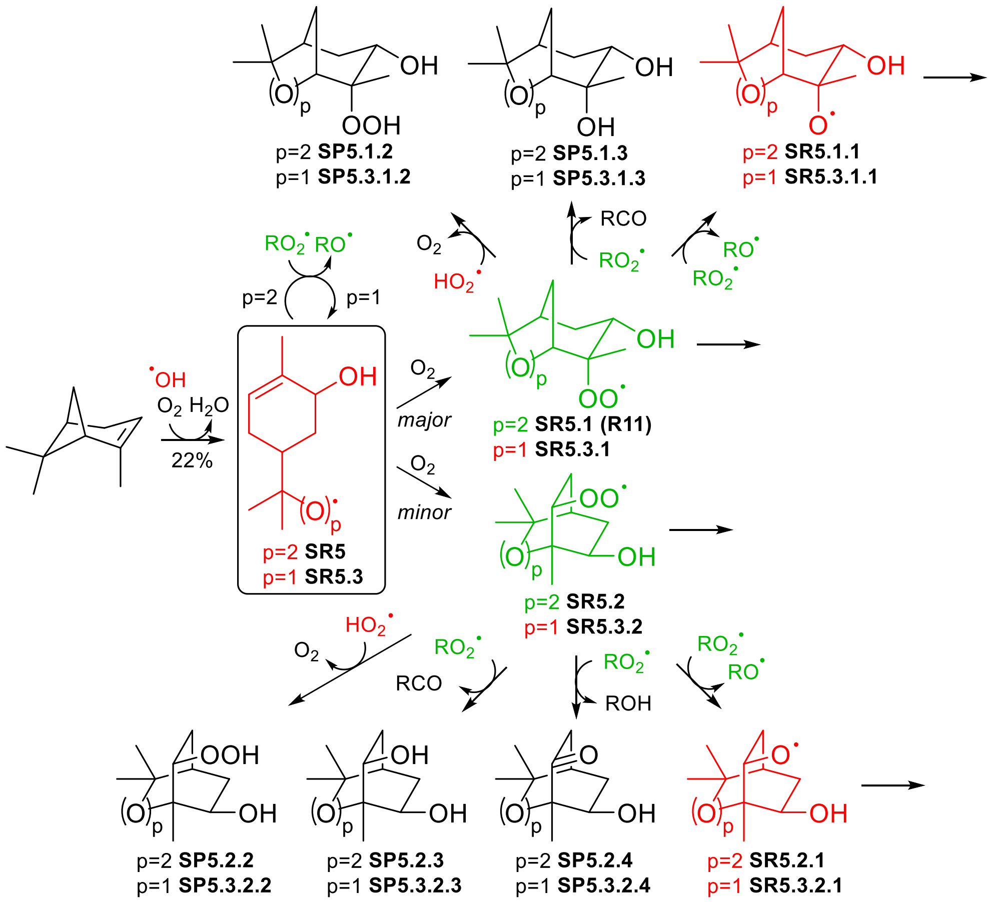

Figure S22: •OH addition to α-pinene leading to peroxy- or oxy-bridged $RO_2^{\bullet}$ and $RO^{\bullet}$ in α-pinene ozonolysis mechanism. Radicals detected by trapping (or their isomers) are shown in green whilst the presence of others (red) could not be confirmed. (red). O_2 release is not shown. RCO represent carbonyl species. Structures and pathway probabilities were obtained from multiple sources [S33-S35]. SR5.1 = R11.

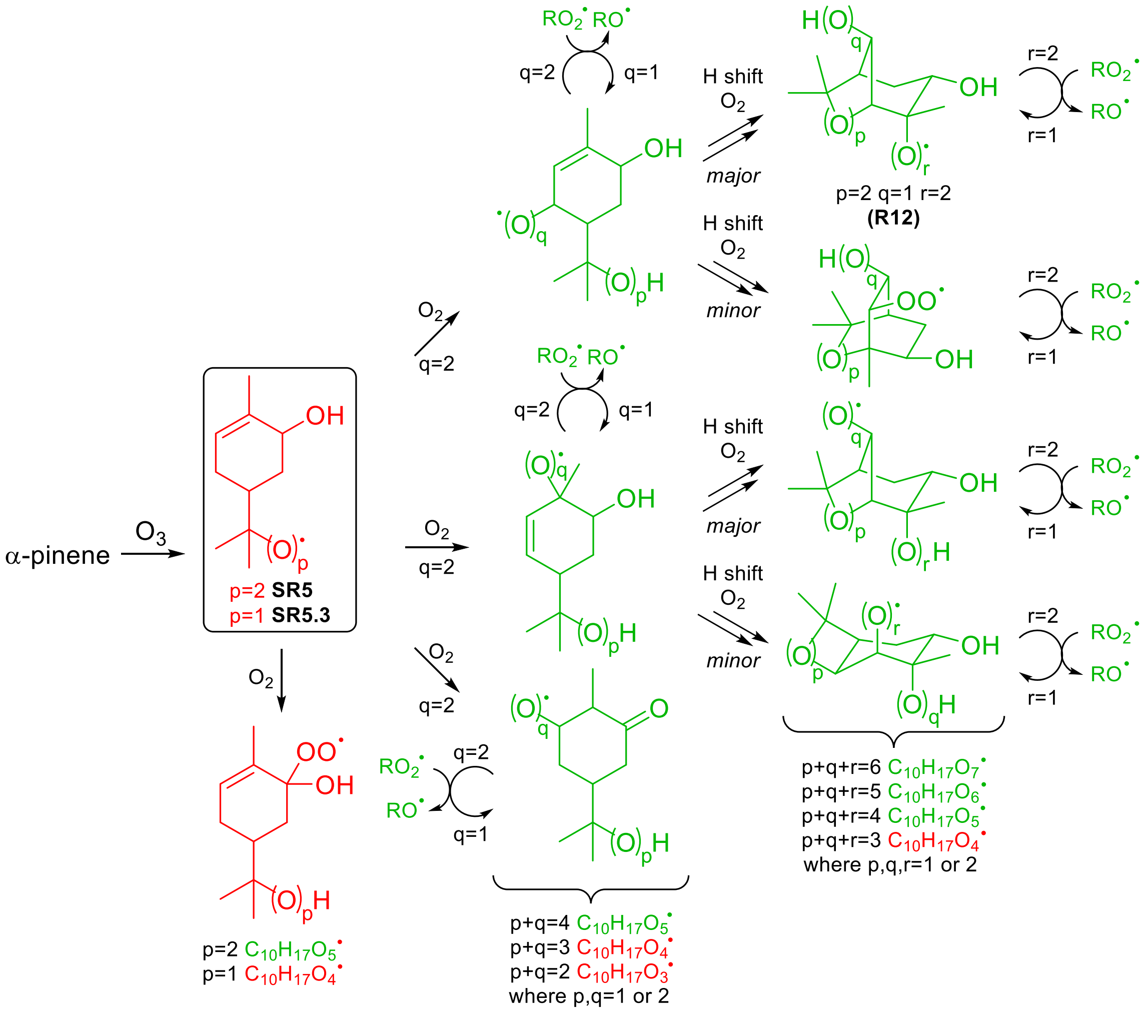

Figure S23: $RO_2^{\bullet}$ and $RO^{\bullet}$ intramolecular autooxidation in α-pinene ozonolysis mechanism. Radicals detected by trapping (or their isomers) are shown in green whilst the presence of others (red) could not be confirmed. (red). O_2 release is not shown. Structures and pathway probabilities were obtained from multiple sources [S33-S35].

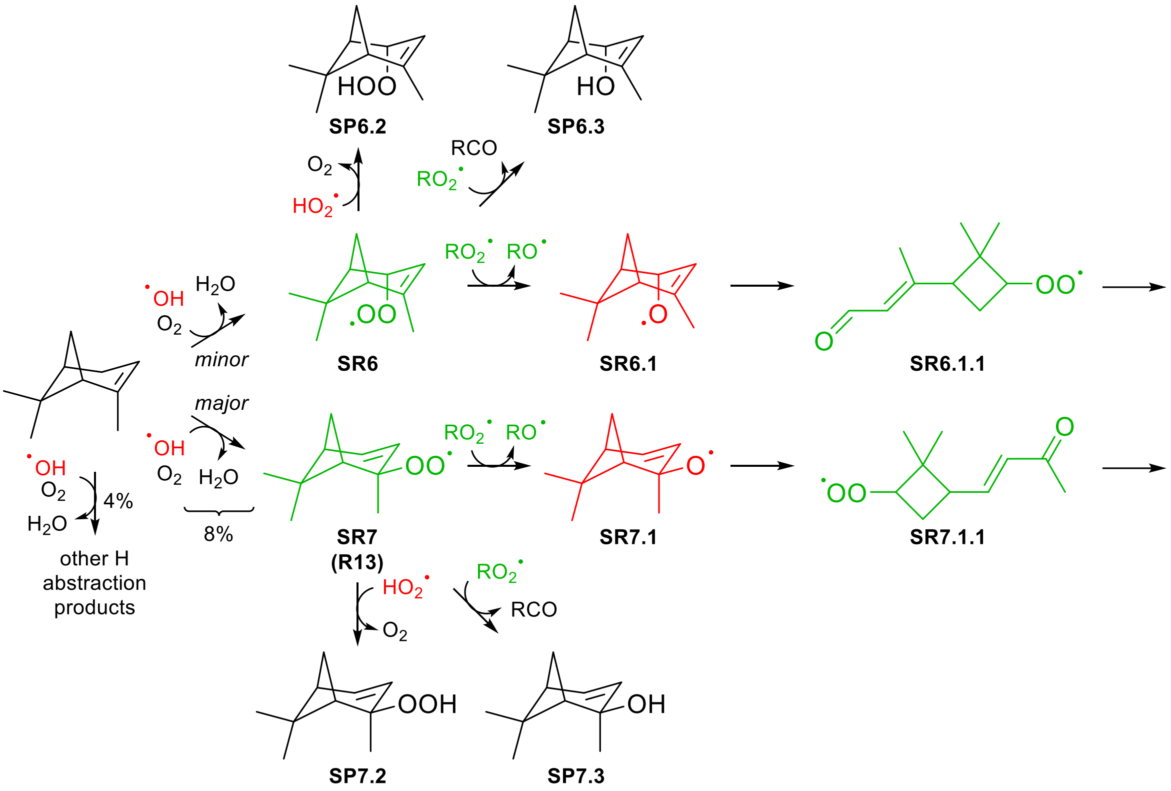

Figure S24: Hydrogen atom abstraction from α-pinene by •OH leading to $RO_2^•$ and RO• in α-pinene ozonolysis mechanism. Radicals detected by trapping (or their isomers) are shown in green whilst the presence of others (red) could not be confirmed. (red). O_2 release is not shown. RCO represent carbonyl species. Structures and pathway probabilities were hypothesised and calculated using multiple sources[S35, S36]. SR7 = R13.

S8.4.3.2. MS data

Table S10. Molecular formulae for unidentified structures not included in Table 1.

Observed *m/z*	Relative intensity / %	Corresponding radical molecular formula
440.2259	0.051	$C_{10}H_{19}O_7^•$
424.2304	0.016	$C_{10}H_{19}O_6^•$
408.2356	0.013	$C_{10}H_{19}O_5^•$

Table S11: Species identified from radical trapping of α-pinene ozonolysis, using MS for characterisation (S5.4). Systematic *m/z* error = -0.0005; random error *m/z* = ±0.0013; 100% intensity = 2.01×10^9 absolute count. ART denotes the trap residue, so R−ART represents a trapped R• radical i.e., −ART is $-CH_2C(CONHC_6H_{11})=CH_2$ for CHANT).

Species	Predicted *m/z*	Intensity relative to control unreacted CHANT standard / %			
		No substrate	No O_2 / No UV[a]	No trap	Trapping reaction[b]
$[CHANT+H]^+$	323.2698	0.019	98.1 / 92.5	0	29.5±1.2
$[$R8/SR1.2−ART+Na$]^+$	388.2100	0	0	0	0.033±0.003
$[$SR1.1.1/R9−ART+Na$]^+$	372.2151[c]	0	0	0	0.023±0.002
$[$R10−ART+Na$]^+$	404.2049[c]	0	0	0.002	0.015±0.001
$[$SR1.2.1.1−ART+Na$]^+$	374.1943	0	0	0	0

[SR1.2.1.2−ART+Na]$^+$	404.2049[c]	0	0	0.002	0.015±0.001
[SR2.1−ART+Na]$^+$	360.2151	0.005	0	0.002	0.015±0.001
[SR2.1.1−ART+Na]$^+$	344.2202	0	0	0	0.006±0.001
[SR2.1.1.1−ART+Na]$^+$	376.2100	0	0	0.007	0.007±0.001
[SR3/SR4−ART+Na]$^+$	374.2307[c]	0	0	0.002	0.003±0.001
[SR3.1/SR4.1−ART+Na]$^+$	358.2358[c]	0	0	0.005	0.006±0.001
[SR5−ART+Na]$^+$	374.2307[c]	0	0	0.002	0.003±0.001
[R11/SR5.2−ART+Na]$^+$	406.2205[c]	0	0	0.011	0.056±0.003
[SR5.1.1/SR5.2.1−ART+Na]$^+$	390.2256[c]	0	0	0	0
[SR5.3−ART+Na]$^+$	358.2358[c]	0	0	0.005	0.005±0.001
[SR5.3.1/SR5.3.2−ART+Na]$^+$	390.2256[c]	0	0	0	0
[SR5.3.1.1/SR5.3.2.1−ART+Na]$^+$	374.2307[c]	0	0	0.002	0.003±0.001
[$C_{10}H_{17}O_4$−ART+Na]$^+$	390.2256[c]	0	0	0	0
[$C_{10}H_{17}O_5$−ART+Na]$^+$	406.2205[c]	0	0	0.011	0.056±0.003
[R12−ART+Na]$^+$	422.2155	0	0	0.005	0.061±0.005
[$C_{10}H_{17}O_7$−ART+Na]$^+$	438.2104	0	0	0	0.011±0.001
[SR6/R13−ART+Na]$^+$	356.2202	0	0	0	0.100±0.006
[SR6.1/SR7.1−ART+Na]$^+$	340.2252	0	0	0	0
[SR6.1.1/SR7.1.1−ART+Na]$^+$	372.2151[c]	0	0	0	0.023±0.002
[Pinaldehyde+Na]$^+$	191.1048[c]	0.023	0	0.676	1.14±0.03
[SP1.1.2/SP1.2.2+Na]$^+$	223.0946[c]	0.005	0	0.070	0.068±0.002
[SP1.1.3/SP1.2.3+Na]$^+$	207.0997[c]	0.003	0	0.511	0.247±0.007
[SP1.2.4+Na]$^+$	205.0841	0	0	0	0
[SP2.1.2+Na]$^+$	195.0997	0.003	0	0.015	0.002±0.001
[SP2.1.3+Na]$^+$	179.1048	0	0	0.011	0.007±0.001
[SP2.1.4+Na]$^+$	177.0891	0.002	0	0.010	0.009±0.001
[SP3.2/SP4.2+Na]$^+$	209.1154[c]	0.009	0	0.080	0.104±0.009
[SP3.3+Na]$^+$	193.1204	0	0	0.049	0.120±0.010
[SP4.3+Na]$^+$	191.1048[c]	0.023	0	0.676	1.14±0.03
[SP5.1.2/SP5.2.2+Na]$^+$	241.1052	0	0	0.048	0.077±0.003
[SP5.1.3/SP5.2.3+Na]$^+$	225.1103[c]	0.001	0	0.918	1.64±0.05
[SP5.2.4+Na]$^+$	223.0946[c]	0.005	0	0.070	0.068±0.002
[SP5.3.1.2/SP5.3.2.2+Na]$^+$	225.1103[c]	0.001	0	0.918	1.64±0.05
[SP5.3.1.3/SP5.3.2.3+Na]$^+$	209.1154[c]	0.009	0	0.080	0.104±0.009
[SP5.3.2.4+Na]$^+$	207.0997[c]	0.003	0	0.511	0.247±0.007
[SP6.2/SP7.2+Na]$^+$	191.1048[c]	0.023	0	0.676	1.14±0.03
[SP6.3/SP7.3+Na]$^+$	175.1099	0	0	0	0

[a]No UV and no N_2 controls combined into single column, with “/” used when values differ. [b]Three repeats undertaken and an average and associated error calculated. [c]Other table entries have predicted species with same *m/z*.

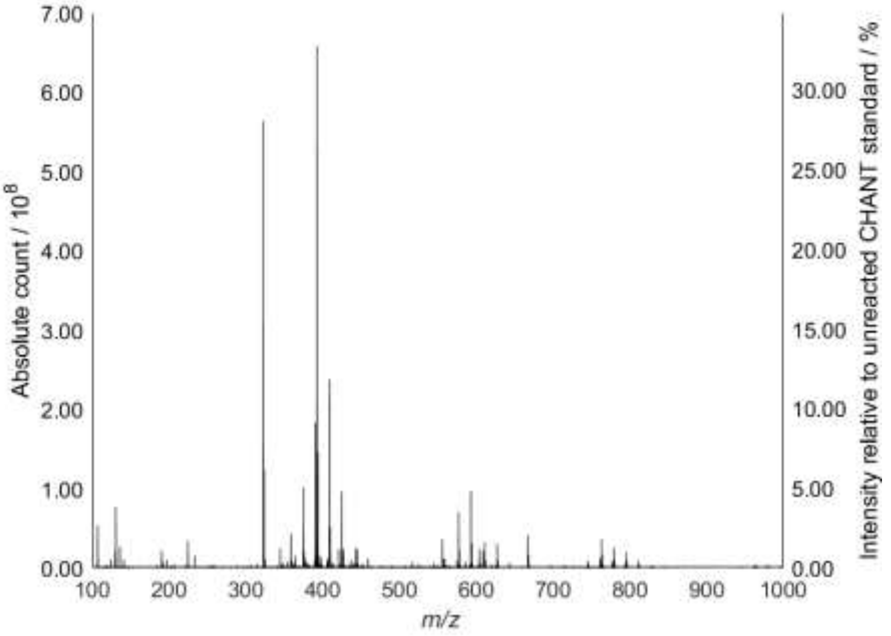

Figure S25: Background corrected mass spectrum from radical trapping of α-pinene ozonolysis (S5.4). 100% intensity = 2.01×10^9 absolute count.

S8.4.3.3. Monoisotopic peak to ^{13}C satellite ratio

Example for SR6/R13−ART:

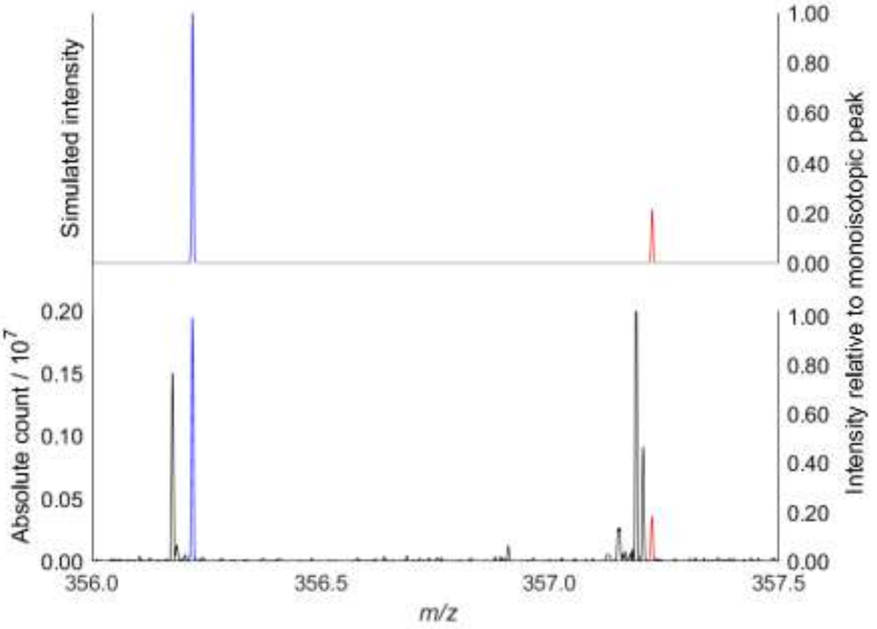

Figure S26: Background corrected mass spectra showing peaks corresponding to monoisotopic CHANT-trapped SR6/R13 (blue) and its corresponding first ^{13}C satellite (red) from radical trapping of α-pinene ozonolysis (S5.4, bottom) and corresponding simulation (top), showing excellent agreement between relative intensities.

[SR6/R13−ART+Na]$^+$ (*m/z* 356.2202) has molecular formula $C_{20}H_{31}NO_3Na$. Ratio of monoisotopic peak (*m/z* 356.2202) intensity: first ^{13}C satellite (*m/z* 357.2235) intensity = 5.36 → corresponds to $C_{18.5}$. Averaged across three repeats = $C_{19.2\pm0.6}$ → implies C_{19-20} → matches predicted C_{20}.

S8.4.3.4. Tandem MS

Example for R8/SR1.2−ART:

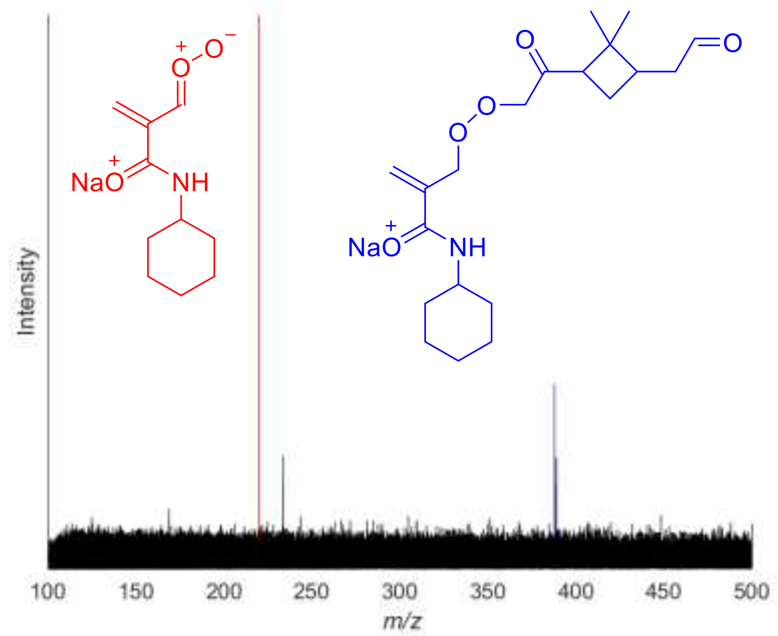

Figure S27: Tandem mass spectrum of the peak corresponding to [R8−ART+Na]$^+$ (*m/z* 388.210, blue) from radical trapping of α-pinene ozonolysis, yielding suggested fragment (*m/z* 220.095, red).

S8.4.3.5. D_2O exchange

Table S12: D exchanges observed for MS peak corresponding to CHANT-trapped SR6/R13. Systematic *m/z* error = -0.0003; random *m/z* error = ±0.0010. ART denotes the trap residue, so R−ART represents a trapped R• radical i.e., −ART is $-CH_2C(CONHC_6H_{11})=CH_2$ for CHANT).

Species	Predicted *m/z* for 0D shift	Predicted D shift	Proportion of total intensity of all D-shifted peaks for each species / %				
			0D	1D	2D	3D	4D
[SR6/R13−ART+Na]$^+$	356.2202	1D	3.0	**97.0**	0	0	0

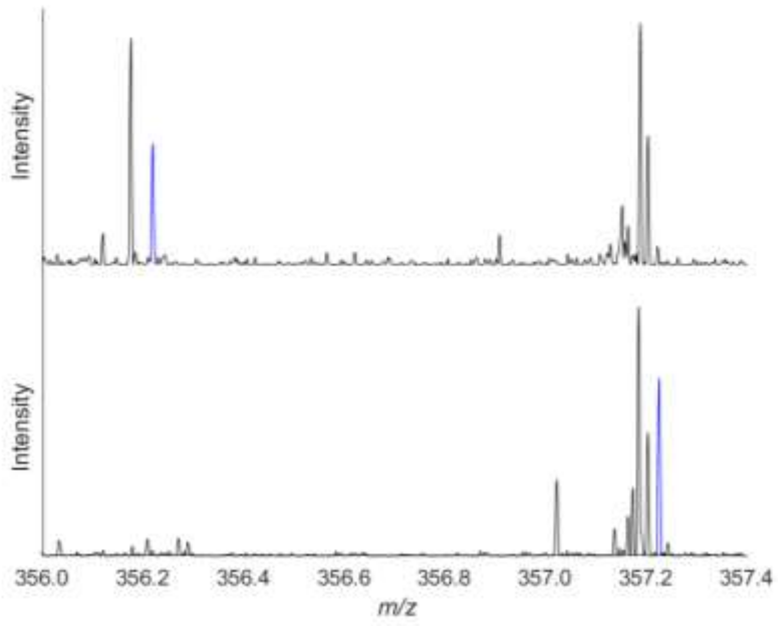

Figure S28: Background corrected mass spectra run in protonated (top) and deuterated (bottom) solvent, showing peaks corresponding to R13−ART (blue) detected from D_2O exchange of CHANT trapping of α-pinene ozonolysis.

S8.5. Gaseous •OH-initiated *n*-nonane degradation

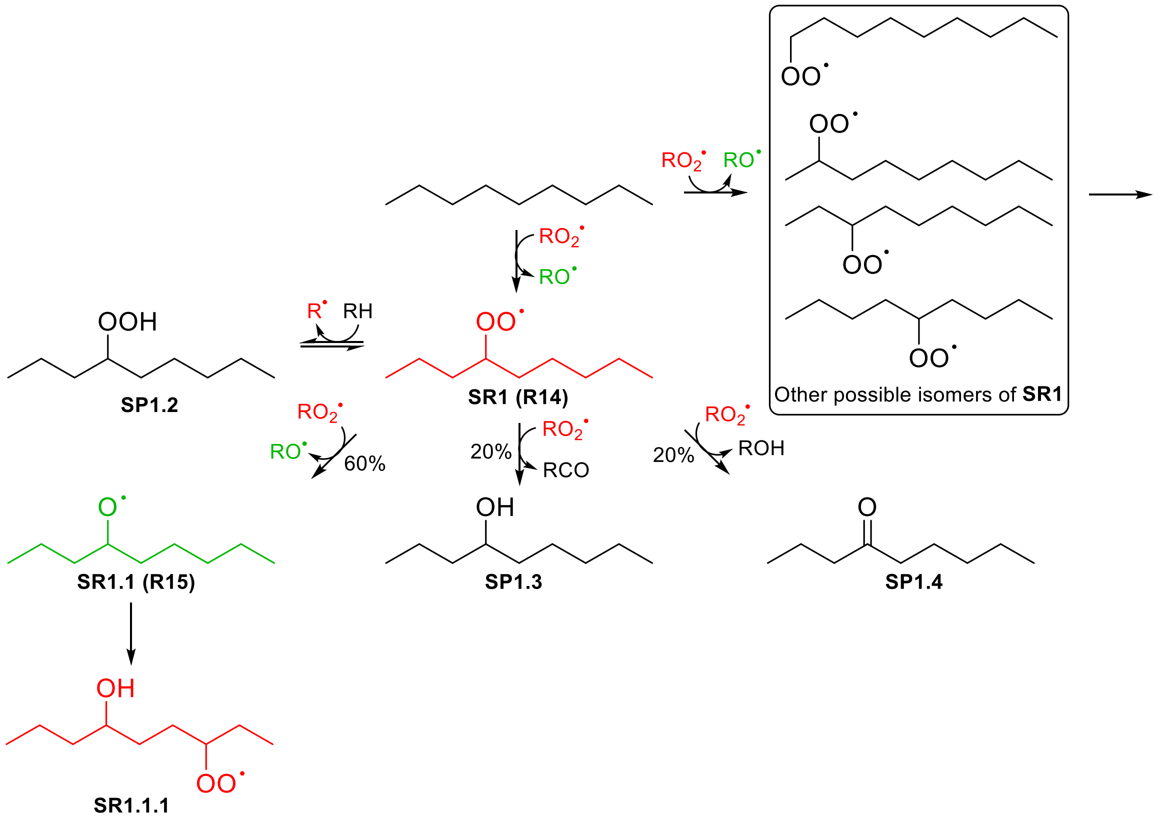

Figure S29: •OH-initiated *n*-nonane degradation mechanism. Radicals detected by trapping (or their isomers) are shown in green whilst the presence of others (red) could not be confirmed. Structures and pathway probabilities were obtained from the MCM[S17]. SR1 = R14, SR1.1 = R15.

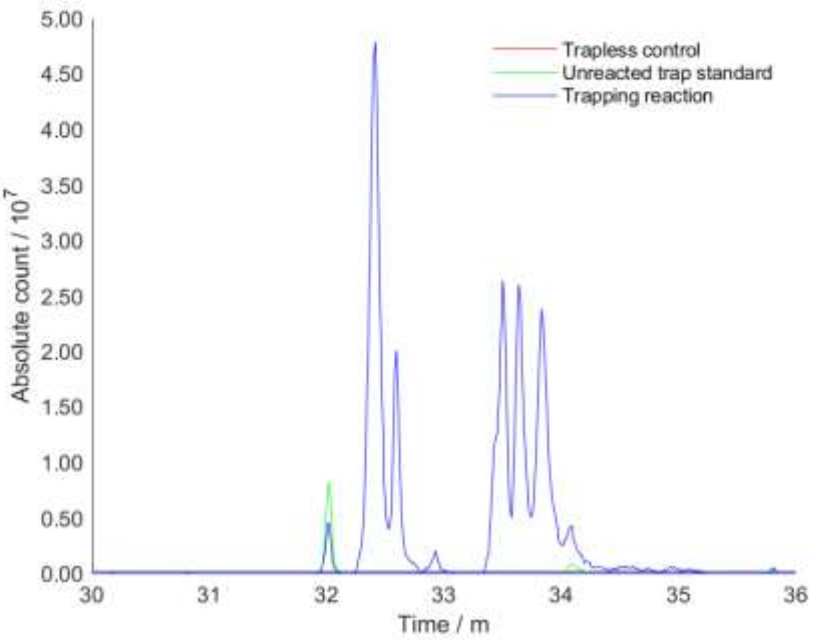

Figure S30: HPLC-MS chromatograms of the peak corresponding to DEADANT-trapped RO• (*m/z* 299.270±0.002) from radical trapping of •OH-initiated *n*-nonane degradation, using DEADANT with controls (S5.5). The five distinct peaks not visible in the controls but observed in the trapping reaction are believed to correspond to the five possible RO• structural isomers.

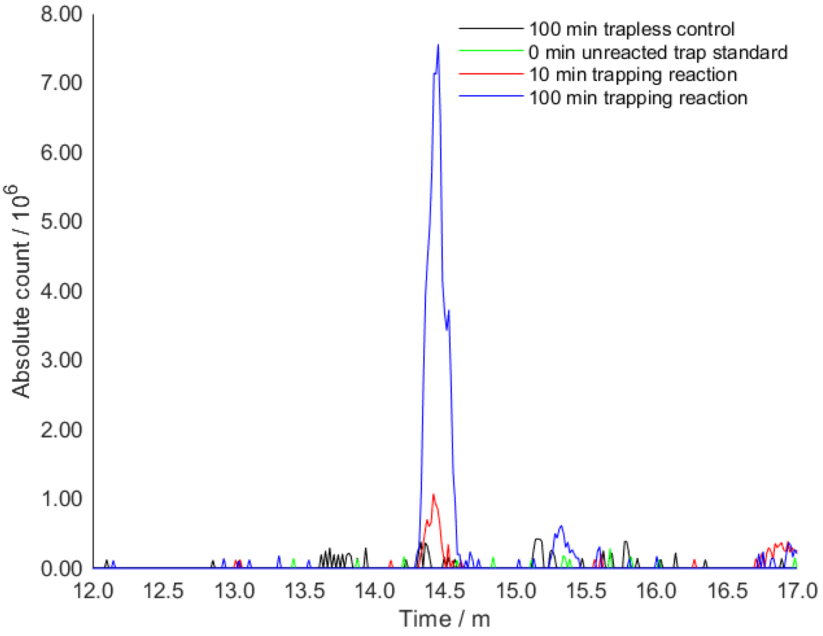

Figure S31: HPLC-MS chromatograms of peaks corresponding to CHANT-trapped RO• (*m/z* 310.275±0.002 and *m/z* 332.257±0.002) from radical trapping of •OH-initiated *n*-nonane degradation, using CHANT for 10 min and 100 min with controls (S5.5). HPLC output was sent to waste between 13.5-14.0 min, to prevent injection of unreacted CHANT.

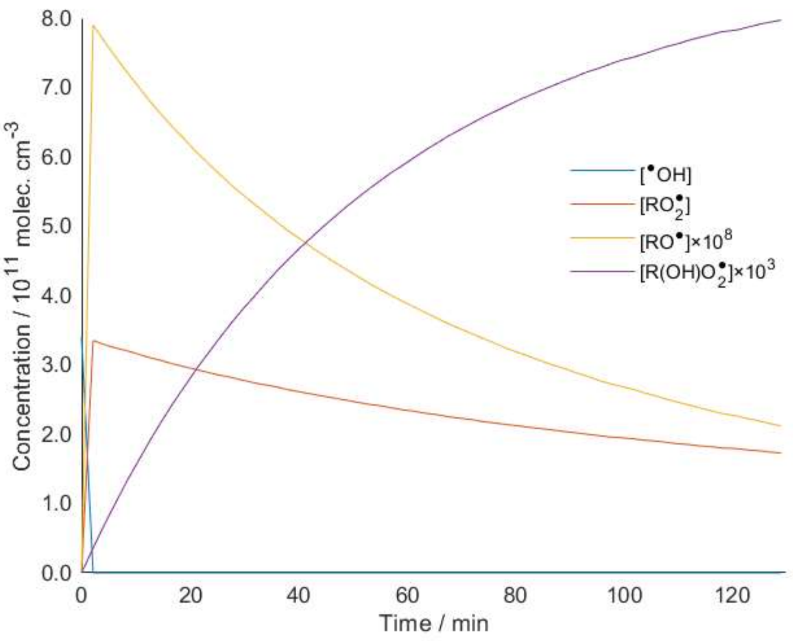

Figure S32: Modelling of •OH-initiated *n*-nonane degradation immediately prior to trapping, showing gaseous [•OH] (blue), [R14] (orange), [R15]×10^8 (yellow) and [SR1.1.1]×10^3 (purple).

S8.6. Barton reaction

The Barton reaction was first developed in 1960 by Derek Barton[S37], who later published a widely accepted mechanism for this reaction (Figure S33)[S9]. Novel radical trapping was applied to the Barton reaction, using isopentyl nitrite as reagent and CHANT as trap (S5.6). MS characterisation showed evidence for previously proposed radicals (Figure S34).

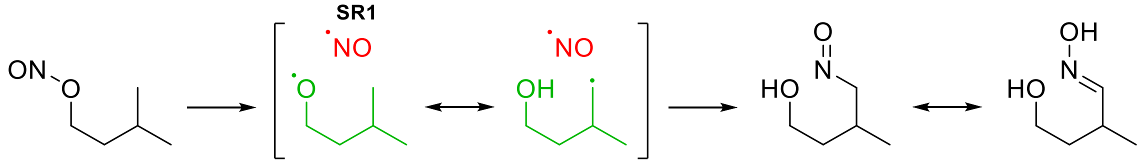

Figure S33: Widely accepted mechanism of Barton reaction for isopentyl nitrite[S9]. Radicals detected by trapping are shown in green whilst the presence of others (red) could not be confirmed.

Table S13: Species identified from radical trapping of the Barton reaction, using MS for characterisation (S5.6). Systematic *m/z* error = -0.0008; random error *m/z* = ±0.0005; 100% intensity = 3.66×10^9 absolute count. ART denotes the trap residue, so R−ART represents a trapped R• radical i.e., −ART is $-CH_2C(CONHC_6H_{11})=CH_2$ for CHANT).

Species		Predicted *m/z*	Intensity relative to unreacted CHANT pre-initiation / % Trapless control	Pre-initiation	Post-trapping
Trap	$[CHANT+H]^+$	323.2698	0.036	100	43.6
Trapped radicals	$[SR1-ART+H]^+$	197.1290	0	0	0
	$[SR1-ART+Na]^+$	219.1109	0	0	0
	$[SR2/SR3-ART+H]^+$	254.2120	0	0	0.026
	$[SR2/SR3-ART+Na]^+$	276.1939	0	0	0.184
	$[SR2/SR3-TEMPO+H]^+$	244.2276	0.003	0	0.003

Peaks corresponding to SR1 were not observed. This was hypothesised to be due to reaction of SR1 (•NO) with alkenes being very slow. In contrast, peaks corresponding to SR2/SR3 were observed, indicating SR2/SR3 were producing during the reaction, offering mechanistic validity.

S8.7. Hunsdiecker reaction

The Hunsdiecker Reaction was discovered by Alexander Borodin in 1861[S38], however was developed into a general method by Clӓre and Heinz Hunsdiecker around 1940[S39]. The Hunsdiecker Reaction involves silver carboxylate decarboxylation and subsequent alkane bromination. A mechanism was later proposed for this reaction (Figure S34)[S40]. Novel radical trapping was applied to the Barton reaction, using silver octanoate as reagent and CHANT as trap (S5.7). MS characterisation showed evidence for previously proposed radicals (Table S14).

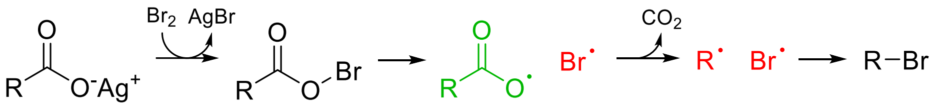

Figure S34: Proposed mechanism for the Hunsdiecker reaction[S40]. Radicals detected by trapping are shown in green whilst the presence of others (red) could not be confirmed.

Table S14: Species identified from radical trapping of the Hunsdiecker reaction, using MS for characterisation (S5.7). Br containing species are shown with ^{79}Br only. 100% = 3.66×10^9 absolute count. Systematic *m/z* error = -0.0002; random error *m/z* = ±0.0005. ART denotes the trap residue, so R−ART represents a trapped R• radical i.e., −ART is −$CH_2C(CONHC_6H_{11})=CH_2$ for CHANT).

Species		Predicted *m/z*	Intensity relative to unreacted CHANT standard / % Trapless control	Pre-initiation	Post-trapping
Trap	[CHANT+H]$^+$	323.2698	0.873	0	39.5
Trapped radicals	[SR1−ART+H]$^+$	310.2382	0	0	0.001
	[SR1−ART+Na]$^+$	332.2202	0.002	0	3.89
	[SR2−ART+H]$^+$	246.0493	0	0	0
	[SR2−ART+Na]$^+$	268.0313	0	0	0
	[SR3−ART+H]$^+$	266.2484	0	0	0
	[SR3−ART+Na]$^+$	288.2303	0	0	0
	[SR3−TEMPO+H]$^+$	256.2640	0	0	0

MS peaks corresponding to SR1 were observed exclusively or much more intensely post-trapping compared to controls, as expected. No other trapped radicals were observed. It was hypothesised that SR2−ART would ionise poorly and hence was unobserved. This may also have been true for SR3−ART. Another possibility was that rapid trapping of SR1 prevented decarboxylation and hence SR3 formation.

S8.8. Decarboxylate aromatic iodination reaction

Perry *et al.* developed a decarboxylate aromatic iodination reaction[S12]. Initially Perry *et al.* suggested this reaction proceeds through a radical decarboxylation-radical recombination pathway (Figure S35) but this was dismissed in favour of a non-radical concerted decarboxylation-iodination pathway[S12]. Novel radical trapping was applied to this reaction, using *p*-anisic acid as reagent and CHANT as trap. MS characterisation showed evidence for previously proposed radicals (Table S15).

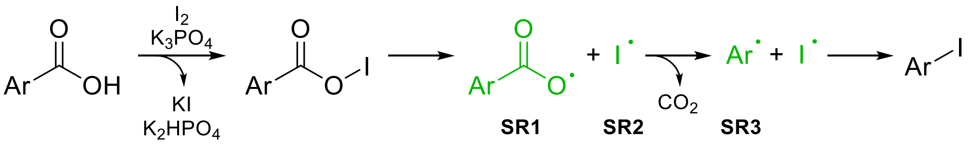

Figure S35: Originally proposed radical mechanism for decarboxylate aromatic iodination reaction developed by Perry *et al.*[S12]. Radicals detected by trapping are shown in green whilst the presence of others (red) could not be confirmed.

Table S15: Species identified from radical trapping of decarboxylate aromatic iodination reaction, using MS for characterisation (S5.8). 100% = 4.71×10^9 absolute count. Systematic *m/z* error = -0.0001; random error *m/z* = ±0.0006. ART denotes the trap residue, so R−ART represents a trapped R• radical i.e., −ART is $-CH_2C(CONHC_6H_{11})=CH_2$ for CHANT).

Species responsible		Predicted *m/z*	Intensity relative to unreacted trap pre-trapping reaction / % Trapless control	Pre-initiation	Post-trapping
Trap	$[CHANT+H]^+$	323.2698	3.08	100	22.0
Trapped radicals	$[SR1-ART+H]^+$	318.1705	0	0	4.32
	$[SR1-ART+Na]^+$	340.1525	0.018	0.004	3.32
	$[SR2-ART+H]^+$	294.0355	0	0.002	0.013
	$[SR2-ART+Na]^+$	316.0174	0	0.009	0.012
	$[SR3-ART+H]^+$	274.1807	0	0	0.011
	$[SR3-ART+Na]^+$	296.1626	0	0	0
	$[SR3-TEMPO+H]^+$	264.1963	0.001	0	0

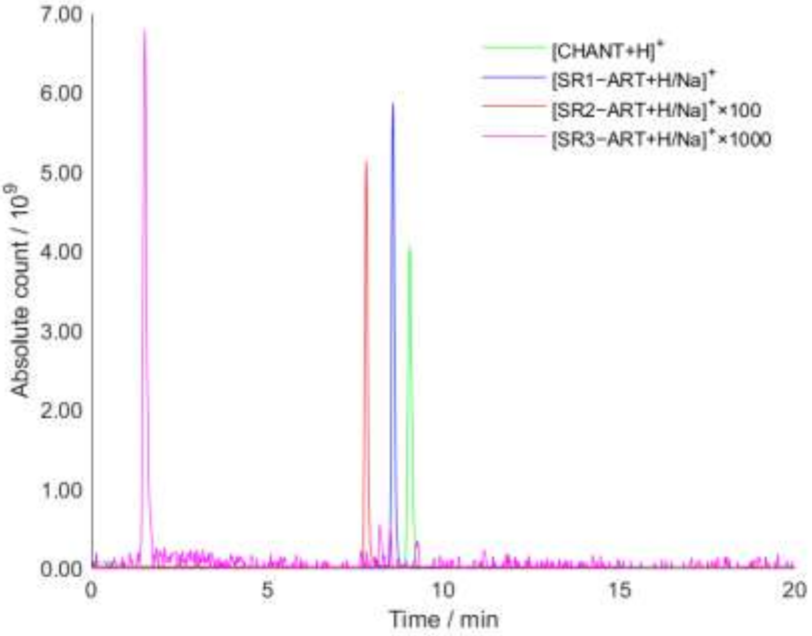

Figure S36: HPLC-MS chromatograms of peaks corresponding to $[CHANT+H]^+$ (*m/z* 323.270±0.002, 9.06 min, green), $[R1-ART+H/Na]^+$ (*m/z* 318.171±0.002 and *m/z* 340.152±0.002, 8.57 min, blue), $[R2-ART+H/Na]^+\times100$ (*m/z* 294.035±0.002 and *m/z* 316.017±0.002, 7.82 min, red) and $[R3-ART+H/Na]^+\times1000$ (*m/z* 274.181±0.002 and *m/z* 296.163±0.002, 1.53 min, pink) detected from TART trapping of radical decarboxylative aromatic iodination, using *p*-anisic acid as substrate and CHANT as trap.

MS peaks corresponding to SR1 and SR3 were observed exclusively or with significantly greater intensity post-trapping compared to controls, as expected. Peaks corresponding to SR2 were observed with similar intensity pre-initiation and post-trapping. These trapped radicals likely arose from trapping of I• (SR2) from intrinsic I_2 homolysis.

Although the concentrations of the trapped radicals have not been quantified, their ionisation efficiencies are unlikely to be significantly different from the other radicals observed in this work, and the high intensity of the MS peaks of the trapped carboxylate radical suggests a radical pathway. This outcome seemingly contradicts the results of the radical clock reaction which suggested a non-radical pathway[S12]. However the radical clock was carried out at *ca.* 1M concentration of I_2 [S12]. Aromatic compounds are known to complex I_2, and under reaction conditions, *ca.* 20-40% of the aromatic reagent will be complexed[S41]. If the O-I bond homolysis of the hypoiodite intermediate is catalysed by the complexation, then cross-coupling of the aromatic radical with the complexed iodine could plausibly outcompete even the very fast radical clock cyclisation (rate constant *ca.* 8×10^9 M^{-1} s^{-1}).

S8.9. Aqueous •OH-initiated degradation of methanol

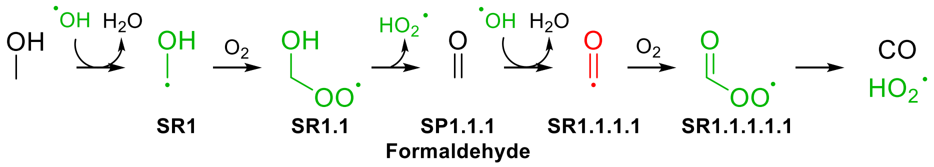

Figure S37: Hypothesised mechanism of aqueous •OH-initiated methanol degradation. Radicals detected by trapping are shown in green whilst the presence of others (red) could not be confirmed.

Table S16: Species identified from DEADANT trapping of •OH-initiated methanol degradation. Systematic *m/z* error = -0.0005; random *m/z* error = ±0.0003; 100% intensity = 1.14×10^9 absolute count.

			Intensity relative to unreacted DEADANT standard / %		
	MS species	Predicted *m/z*	No substrate	Trapless control	Trapping reaction
Trap	[DEADANT+H]$^+$	312.2651	13.1	0.030	6.53
Trapped radicals	[SR1−ART+H]$^+$	187.1446	0	0	0.013
	[SR1−TEMPO+H]$^+$	188.1650	0	0	0
	[SR1.1−ART+H]$^+$	219.1345	0.004	0	0.141
	[SR1.1.1.1−ART+H]$^+$	185.1290	0	0	0
	[SR1.1.1.1−TEMPO+H]$^+$	186.1494	0	0	0
	[SR1.1.1.1.1−ART+H]$^+$	217.1188	0.090	0	0.385
	[OH−ART+H]$^+$	173.1290	0.018	0	0.018
	[HO_2−ART+H]$^+$	189.1239	0	0	0.007

S8.10. Aqueous •OH-initiated degradation of thymine

Unquenched radicals produced as by-products during oxidative metabolism, such as reactive oxygen species (ROS) $O_2^{\bullet-}$ and •OH, cause cell damage. •OH is believed to be able to induce DNA crosslinking, which changes the DNA structure and could cause the cell to behave abnormally, either causing cell death or incorrect DNA replication, which could eventually cause cancer[S42]. Therefore, novel radical trapping was used to investigate the mechanism of aqueous •OH-initiated thymine degradation (Figure S38), using DEADANT as trap. DEADANT dissolved in the acidic •OH-initiated thymine degradation aqueous system, due to its basic 3° amine functionality. MS characterisation indicated presence of hypothesised trapped radicals (Table S17).

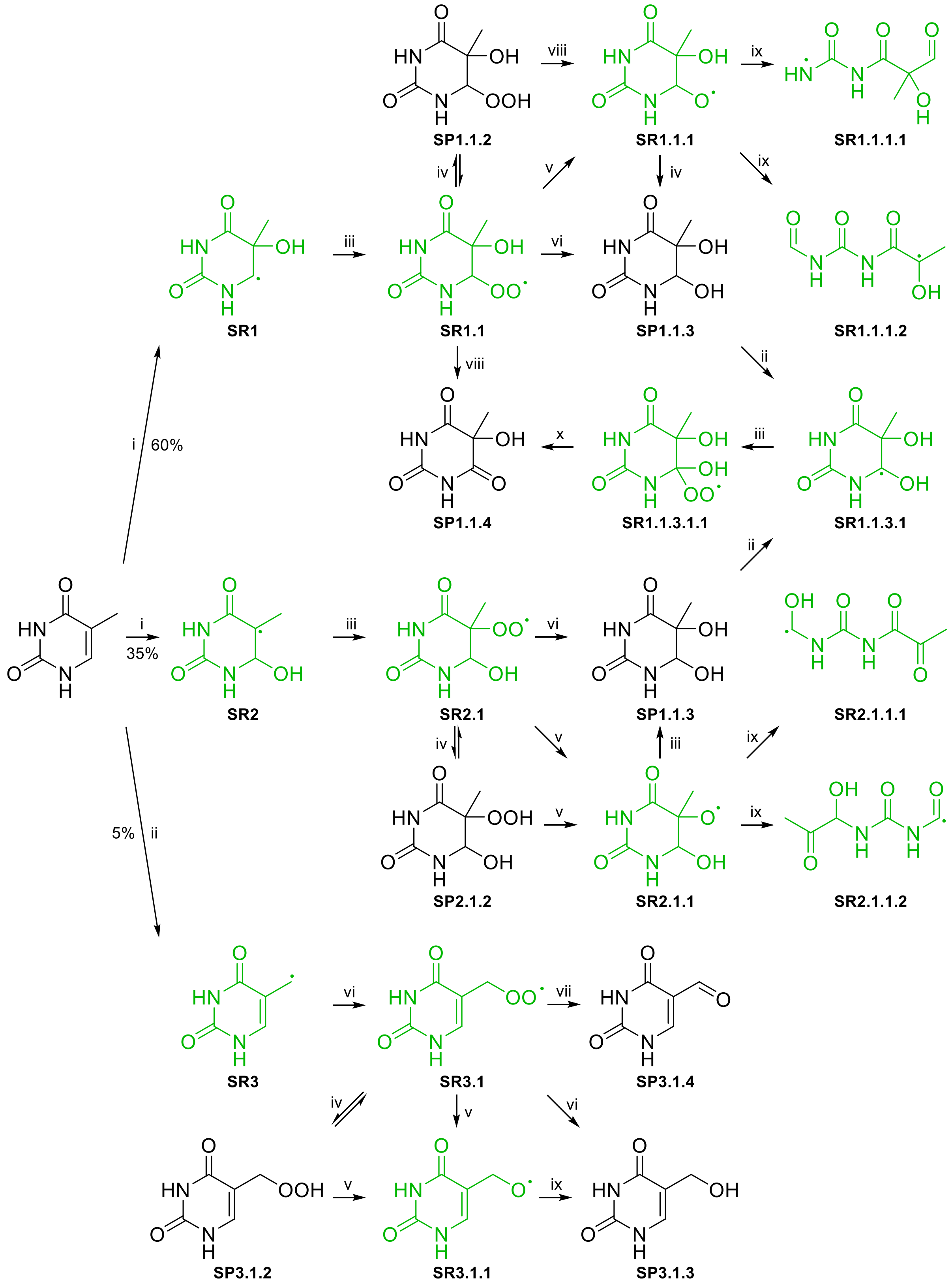

Figure S38: Non-comprehensive mechanism of aqueous •OH-initiated thymine degradation. Structures and pathway probabilities were obtained or hypothesised using literature sources[S43]. i) + •OH. ii) + •OH, - H_2O. iii) + O_2. iv) + RH, - R•. v) + RO_2•, - RO•. vi) + RO_2•, - RCO. vii) + RO_2•, - ROH. viii) - •OH. ix) Fragmentation. x) - HO_2•. Radical trapping indicated presence of all (or their isomers) radicals (green).

Table S17: Species identified from radical trapping of •OH-initiated thymine degradation, using MS for characterisation (S5.10). Systematic *m/z* error = -0.0002; random *m/z* error = ±0.0005; 100% intensity = 1.09x10^9 absolute count.

	Species	Predicted *m/z*	Intensity relative to unreacted trap standard / % Trapless control	Standard	Trapping reaction
Trap	[DEADANT+H]$^+$	312.2651	0	100	51.4
Reagents	[Thymine+Na]$^+$	149.0327	0.024	0	0.036
Trapped radicals	[SR1/SR2–ART+H]$^+$	299.1719	0	0	1.62
	[SR1/SR2–TEMPO+H]$^+$	300.1923	0	0	1.45
	[SR1.1/SR2.1–ART+H]$^+$	331.1618	0	0	0.456
	[SR1.1.1/SR2.1.1–ART+H]$^+$	315.1668[a]	0	0	1.55
	[SR1.1.1.1/SR1.1.1.2–ART+H]$^+$	315.1668[a]	0	0	1.55
	[SR1.1.1.2–TEMPO+H]$^+$	316.1872[a]	0	0	0.727
	[SR1.1.3.1–ART+H]$^+$	315.1668[a]	0	0	1.55
	[SR1.1.3.1–TEMPO+H]$^+$	316.1872[a]	0	0	0.727
	[SR1.1.3.1.1–ART+H]$^+$	347.1567	0	0	0.134
	[SR2.1.1.1/SR2.1.1.2–ART+H]$^+$	315.1668[a]	0	0	1.55
	[SR2.1.1.1/SR2.1.1.2–TEMPO+H]$^+$	316.1872[a]	0	0	0.727
	[SR3–ART+H]$^+$	281.1614	0	0	0.033
	[SR3–TEMPO+H]$^+$	282.1818	0	0	1.23
	[SR3.1–ART+H]$^+$	313.1512	0	0	0.090
	[SR3.1.1–ART+H]$^+$	297.1563	0	0	0.032
	[OH–ART+H]$^+$	173.1290	0	0	0.010
	[HO$_2$–ART+H]$^+$	189.1239	0	0	0
Products	[SP1.1.2/SP2.1.2+Na]$^+$	199.0331	0.042	0	0.025
	[SP1.1.3+Na]$^+$	183.0382	0.064	0	0.054
	[SP1.1.4+Na]$^+$	181.0225[a]	0.033	0	0.022
	[SP3.1.2+Na]$^+$	181.0225[a]	0.033	0	0.022
	[SP3.1.3+Na]$^+$	165.0276	0.003	0	0.007
	[SP3.1.4+Na]$^+$	163.0120	0.007	0	0

[a]Other table entries have predicted species with same *m/z*.

Peaks corresponding to most products were observed with similar intensities in the trapless control and trapping reaction. In contrast, peaks corresponding to most hypothesised trapped radicals were observed exclusively post-trapping reaction.

S8.11. Aqueous •OH-initiated degradation of glucose

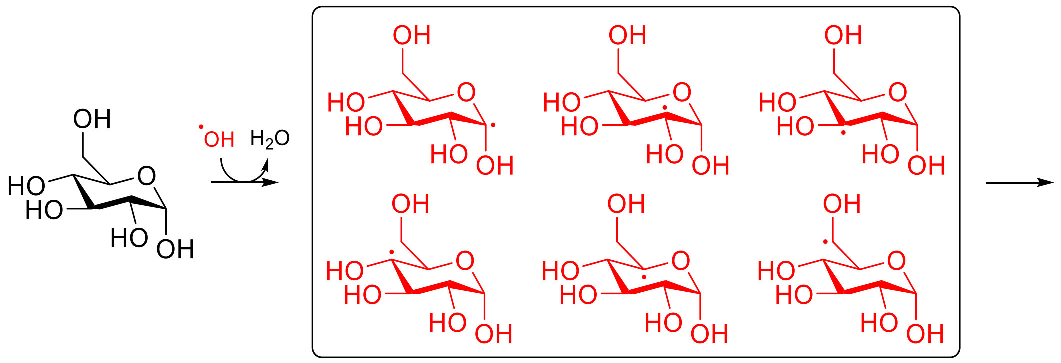

Figure S39: Plausible radicals formed in •OH-initiated glucose degradation.

Table S18: Identified radicals from the most intense MS peaks attributed to monomeric non-fragmented, DEADANT-trapped radicals in •OH-initiated glucose degradation. Molecular formula limits were set as $C_{14}H_{0\text{-}28}N_2O_{1\text{-}12}$ and *m/z* limits 100-500. Unreasonable molecular formulae were eliminated. RCO represents formation of carbonyl from an existing alcohol group, i.e. R• and R(CO)• differ by H_2. Systematic *m/z* error = -0.0002; random *m/z* error = ±0.0005; 100% intensity = 1.09×10^9 absolute count.

Entry	Observed *m/z*	Intensity relative to DEADANT standard / %	Corresponding radical molecular formula	Suggested radical functionality
1	365.1566	0.142	$C_6H_9O_8$•	$R(CO)O_2$•<br>R(CO)(OH)O•<br>R(CO)(OOH)•<br>$R(CO)(OH)_2$•
2	363.1410	0.140	$C_6H_7O_8$•	$R(CO)_2O_2$•<br>$R(CO)_2(OH)O$•<br>$R(CO)_2(OOH)$•<br>$R(CO)_2(OH)_2$•
3	381.1516	0.114	$C_6H_9O_9$•	$R(CO)(OH)O_2$•<br>R(CO)(OOH)O•<br>$R(CO)(OH)_2O$•<br>R(CO)(OH)(OOH)•<br>$R(CO)(OH)_3$•
4	379.1361	0.081	$C_6H_7O_9$•	$R(CO)_2(OH)O_2$•<br>$R(CO)_2(OOH)O$•<br>$R(CO)_2(OH)_2O$•<br>$R(CO)_2(OH)(OOH)$•<br>$R(CO)_2(OH)_3$•
5	347.1460	0.072	$C_6H_7O_7$•	$R(CO)_2O$•<br>$R(CO)_2(OH)$•
6	349.1617	0.049	$C_6H_9O_7$•	R(CO)O•<br>R(CO)(OH)•
7	397.1467	0.038	$C_6H_9O_{10}$•	$R(CO)(OOH)O_2$•<br>$R(CO)(OH)_2O_2$•<br>R(CO)(OH)(OOH)O•<br>$R(CO)(OH)_3O$•<br>$R(CO)(OOH)_2$•<br>$R(CO)(OH)_2(OOH)$•<br>$R(CO)(OH)_4$•
8	367.1722	0.034	$C_6H_{11}O_8$•	RO_2•<br>R(OH)O•<br>R(OOH)•<br>$R(OH)_2$•
9	383.1673	0.027	$C_6H_{11}O_9$•	$R(OH)O_2$•<br>R(OOH)O•<br>$R(OH)_2O$•<br>R(OH)(OOH)•<br>$R(OH)_3$•
10	331.1509	0.023	$C_6H_7O_6$•	$R(CO)_2$•
11	333.1662	0.022	$C_6H_9O_6$•	R(CO)•
12	361.1247	0.020	$C_6H_5O_8$•	$R(CO)_3O_2$•<br>$R(CO)_3(OH)O$•<br>$R(CO)_3(OOH)$•<br>$R(CO)_3(OH)_2$•

13	345.1298	0.017	$C_6H_5O_7^•$	$R(CO)_3O^•$<br>$R(CO)_3(OH)^•$

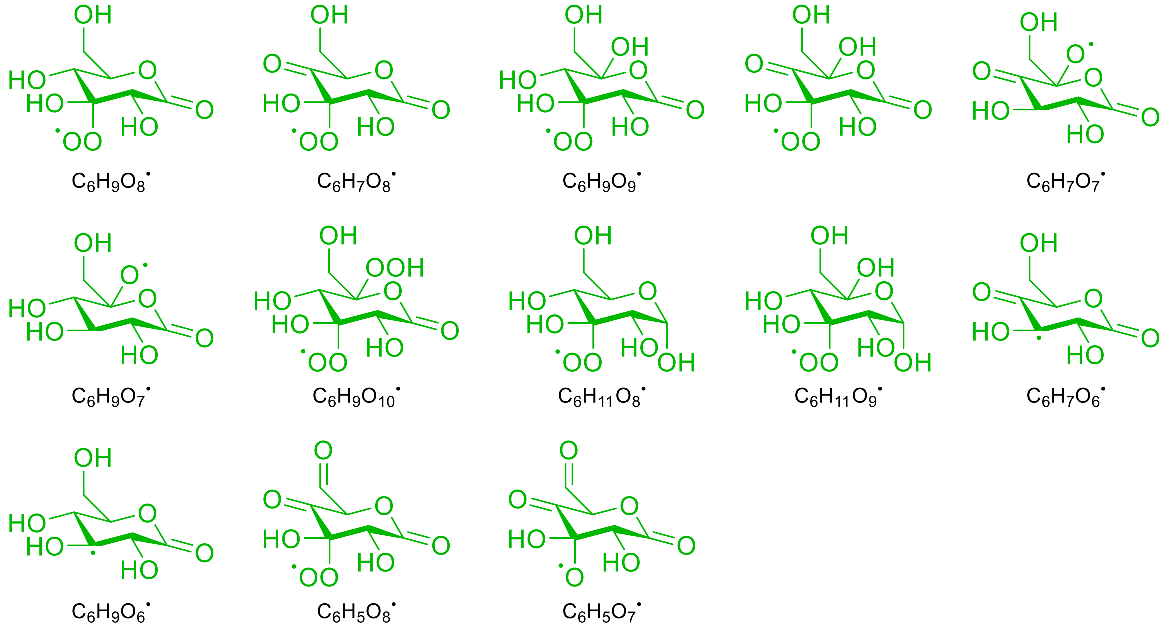

Figure S40: Example structures possibly produced during from •OH-initiated glucose degradation. Radical trapping indicated presence of all radicals (or their isomers) (green).

S8.12. Aqueous $^{\bullet}$OH-initiated degradation of ascorbic acid

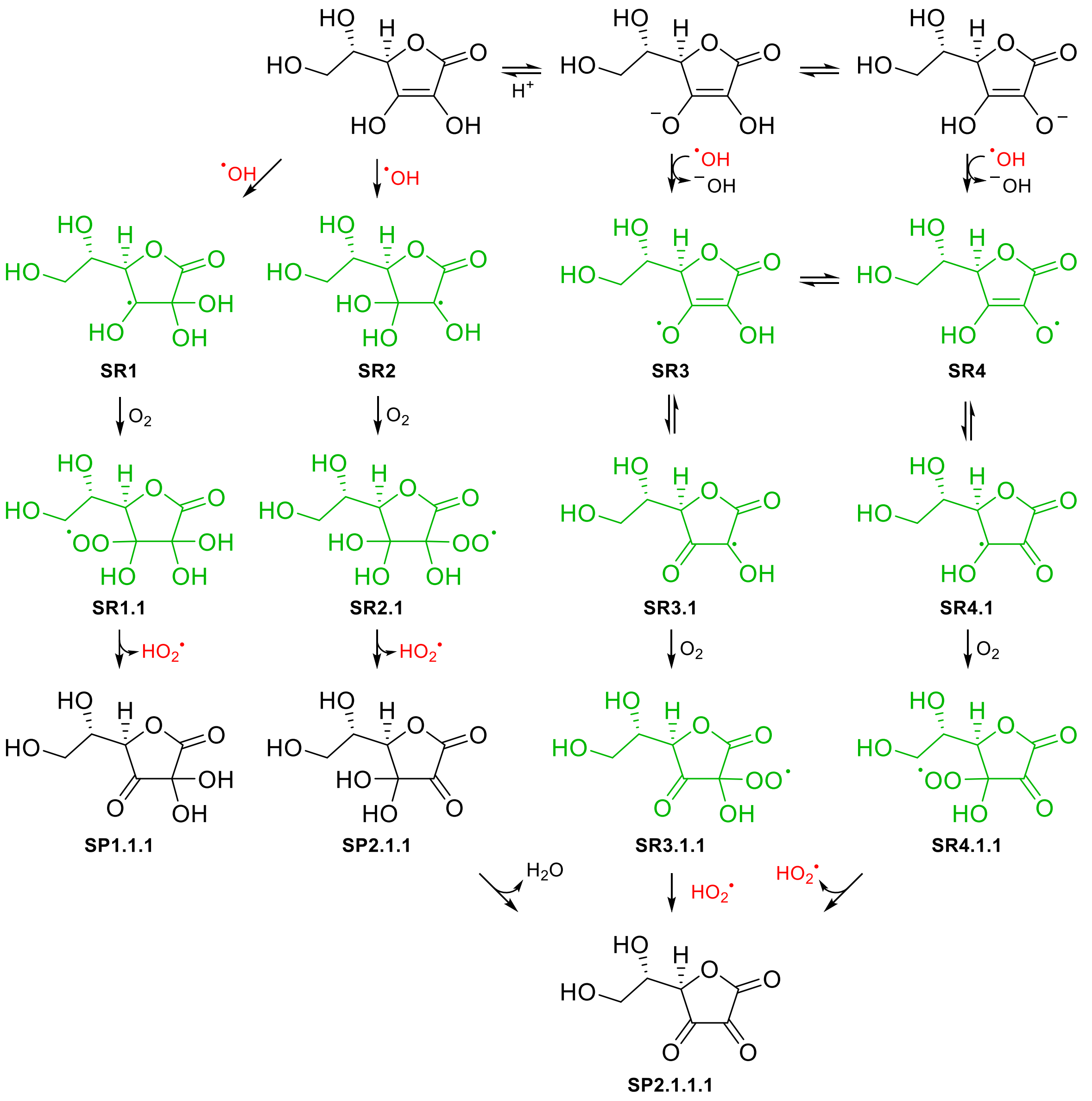

Figure S41: $^{\bullet}$OH-initiated ascorbic acid degradation in aqueous solution. Radicals detected by trapping (or their isomers) are shown in green whilst the presence of others (red) could not be confirmed.

Table S19: Species identified from DEADANT trapping of •OH-initiated ascorbic acid degradation. Systematic *m/z* error = -0.0005; random *m/z* error = ±0.0008; 100% intensity = 1.09×10^{10} absolute count[a].

	Species	Predicted *m/z*	Intensity relative to unreacted trap standard[a] / %: Trapless control	Standard[a]	Trapping reaction
Trap	$[DEADANT+H]^+$	312.2651	0	100	0
Reactants	$[Ascorbic\ acid+Na]^+$	199.0219	0.0004	0	0
Trapped radicals	$[SR1/SR2{-}ART+H]^+$	349.1611	0	0	0.0021
	$[SR1/SR2{-}TEMPO+H]^+$	350.1815	0	0	0.0008
	$[SR1.1/SR2.1{-}ART+H]^+$	381.1509	0	0	0.0039
	$[SR3/SR4{-}ART+H]^+$	331.1505[b]	0	0	0.0050
	$[SR3.1/SR4.1{-}ART+H]^+$	331.1505[b]	0	0	0.0050
	$[SR3.1/SR4.1{-}TEMPO+H]^+$	332.1709[b]	0	0	0.0466
	$[SR3.1.1/SR4.1.1{-}ART+H]^+$	363.1404	0	0	0.0315
	$[OH{-}ART+H]^+$	173.1290	0	0	0
	$[HO_2{-}ART+H]^+$	189.1239	0	0	0
Products	$[SP1.1.1/SP2.1.1+Na]^+$	215.0168	0.0241	0	0.0461
	$[SP2.1.1.1+Na]^+$	197.0062	0.0007	0	0.0017

[a]Standard scaled ×10 to match dilution of samples. [b]Other table entries have predicted species with same *m/z*.

S9. Spectra

S9.1. Trap synthesis

S9.1.1. Methyl 2-(TEMPOmethyl)acrylate

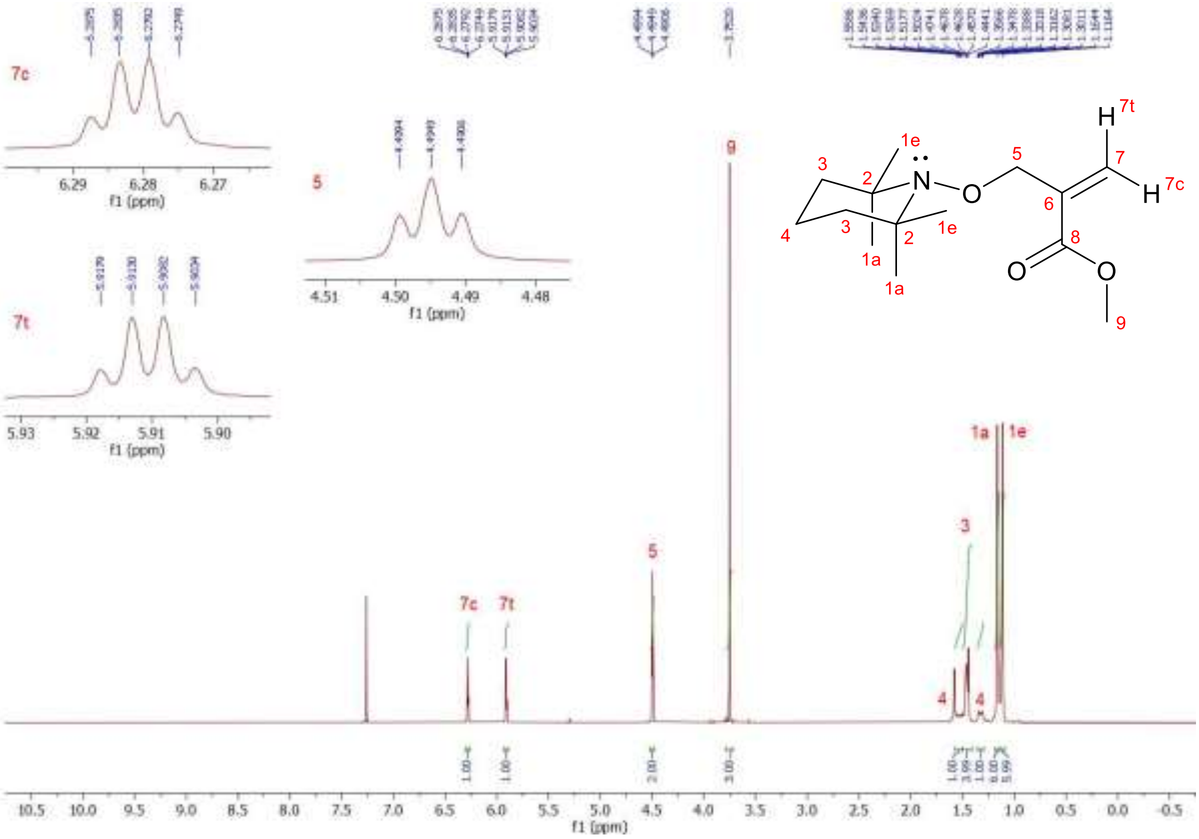

Figure S42: Methyl 2-(TEMPOmethyl)acrylate ^{1}H NMR spectrum ($CDCl_3$, 400 MHz, 298 K).

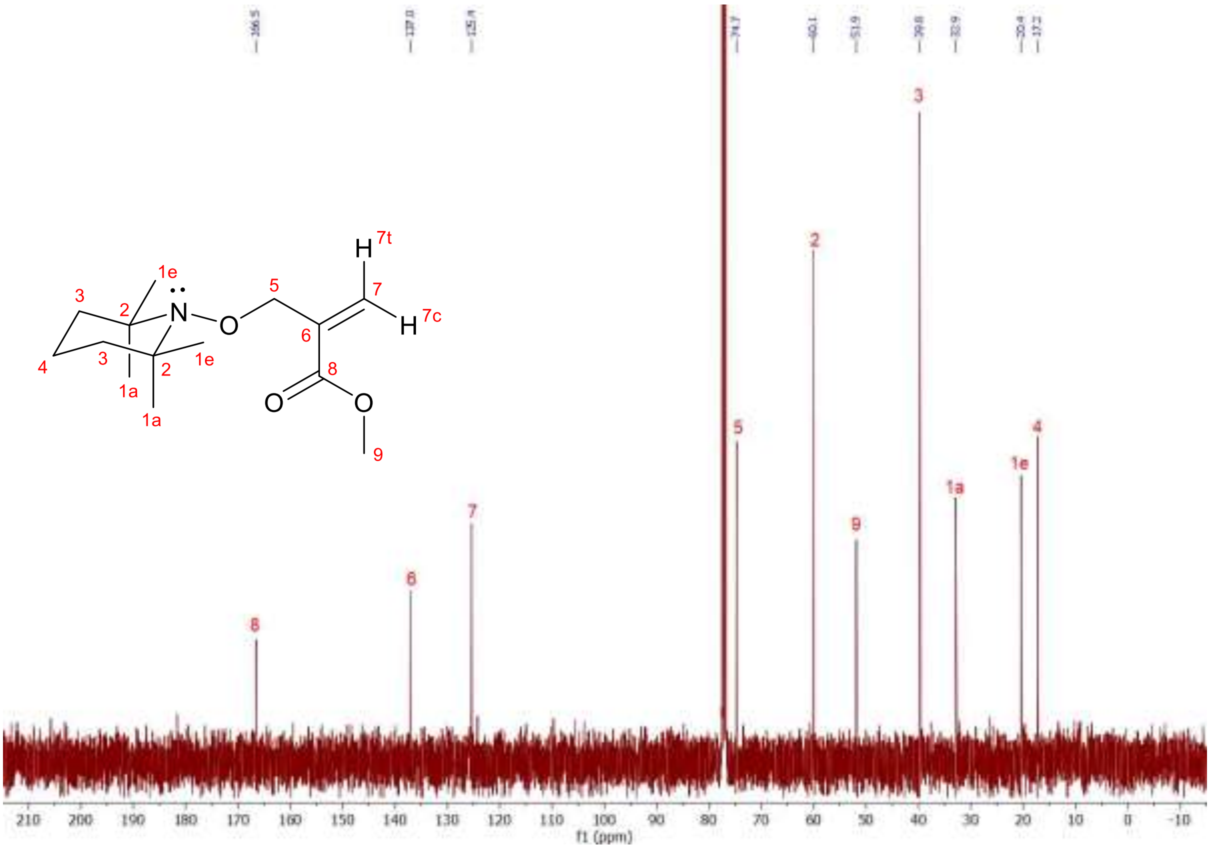

Figure S43: Methyl 2-(TEMPOmethyl)acrylate ^{13}C NMR spectrum ($CDCl_3$, 100 MHz, 298 K).

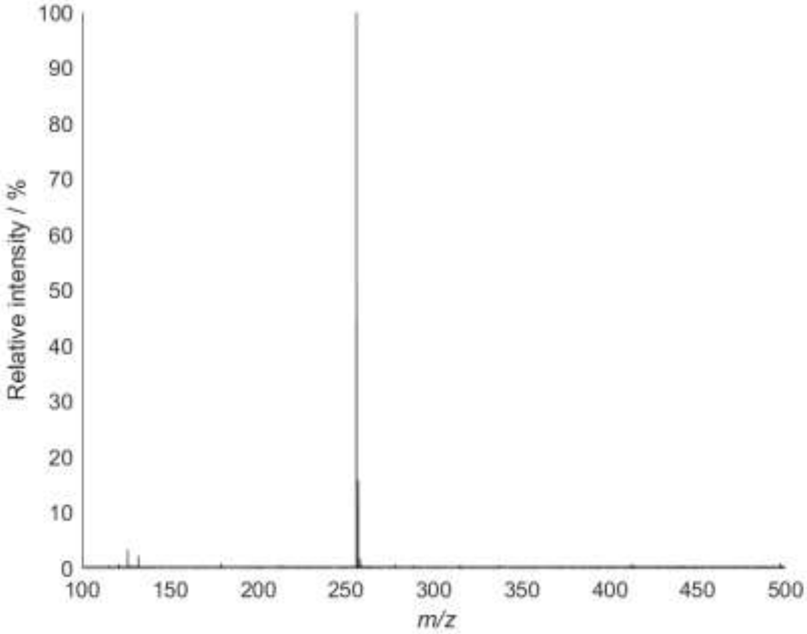

Figure S44: Methyl 2-(TEMPOmethyl)acrylate mass spectrum (Pos ESI).

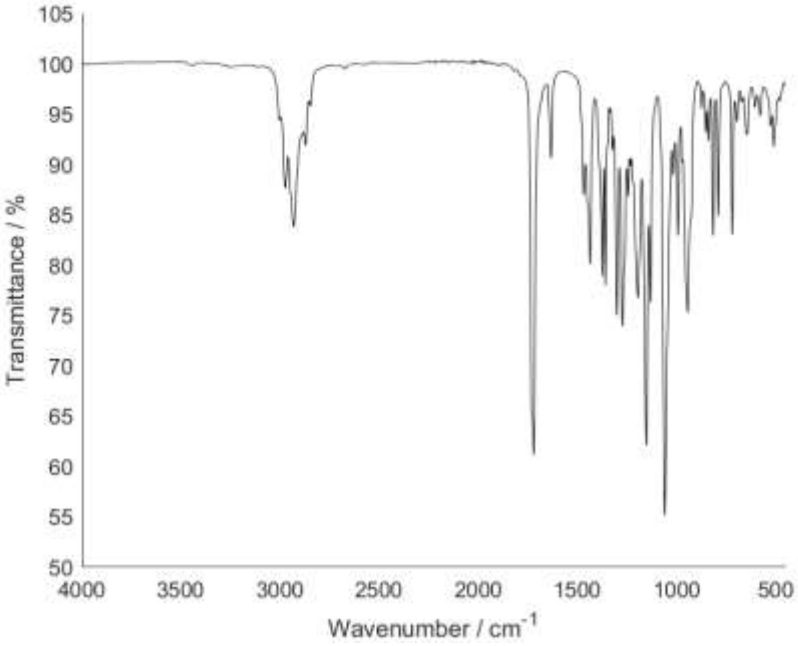

Figure S45: Methyl 2-(TEMPOmethyl)acrylate IR spectrum.

S9.1.2. 2−(TEMPOmethyl)acrylic acid

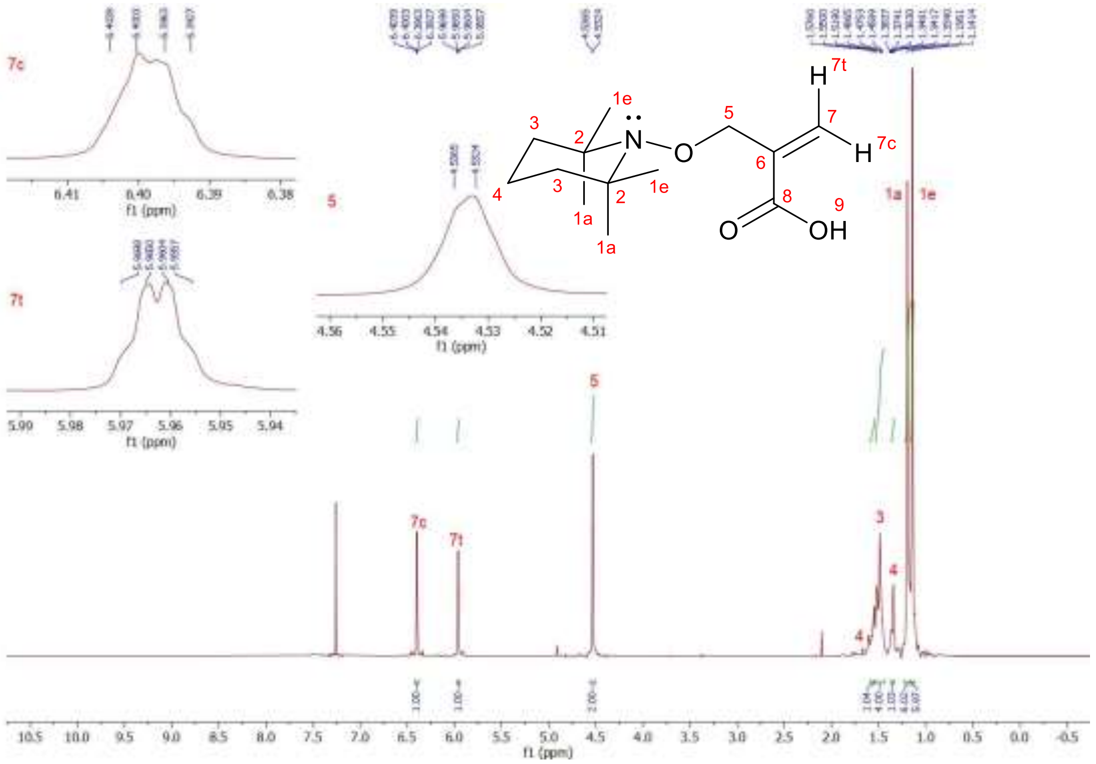

Figure S46: 2-(TEMPOmethyl)acrylic acid ^1H NMR spectrum ($CDCl_3$, 400 MHz, 298 K).

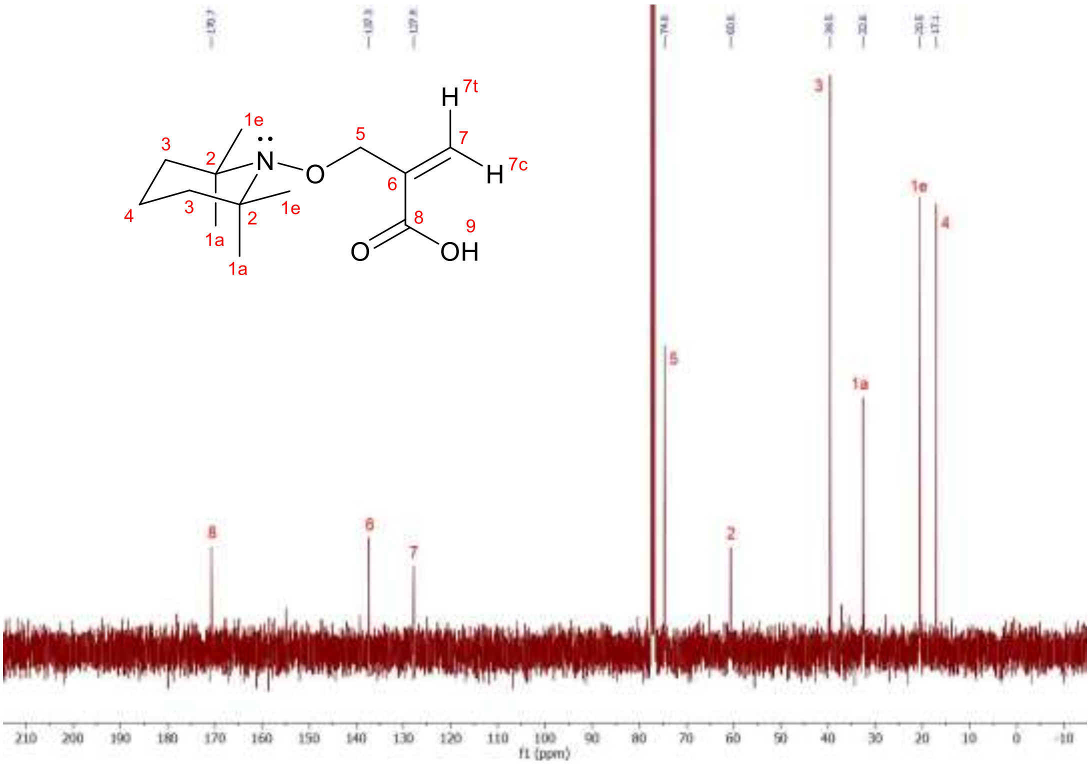

Figure S47: 2-(TEMPOmethyl)acrylic acid ^{13}C NMR spectrum ($CDCl_3$, 100 MHz, 298 K).

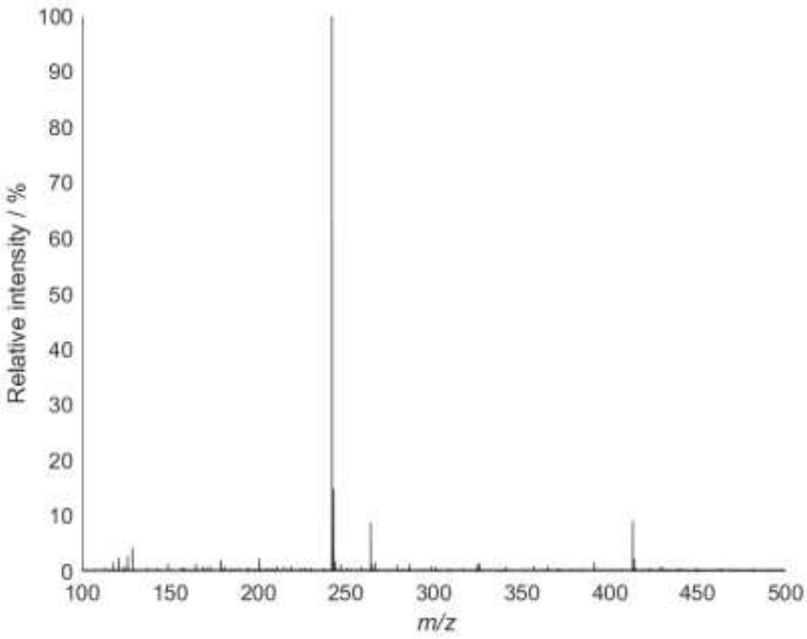

Figure S48: 2-(TEMPOmethyl)acrylic acid mass spectrum (Pos ESI).

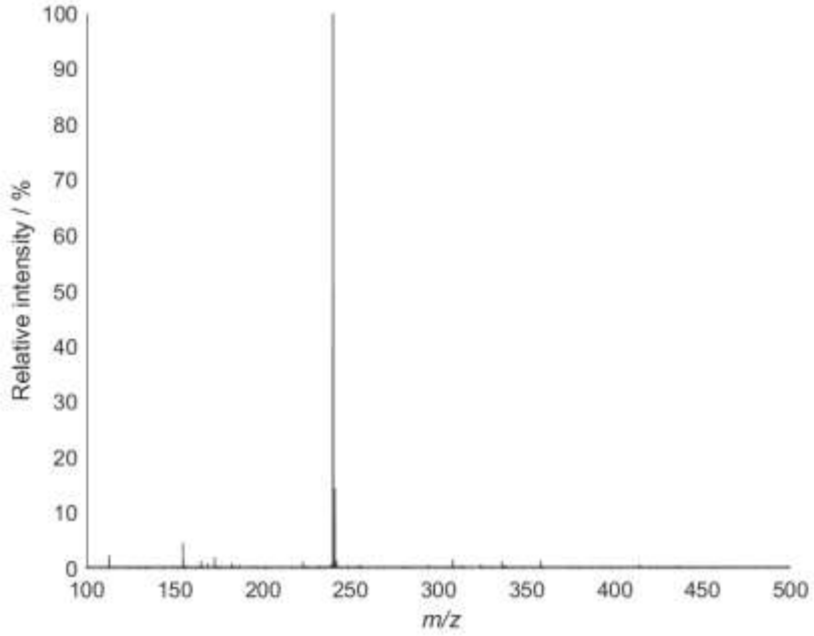

Figure S49: 2-(TEMPOmethyl)acrylic acid mass spectrum (Neg ESI).

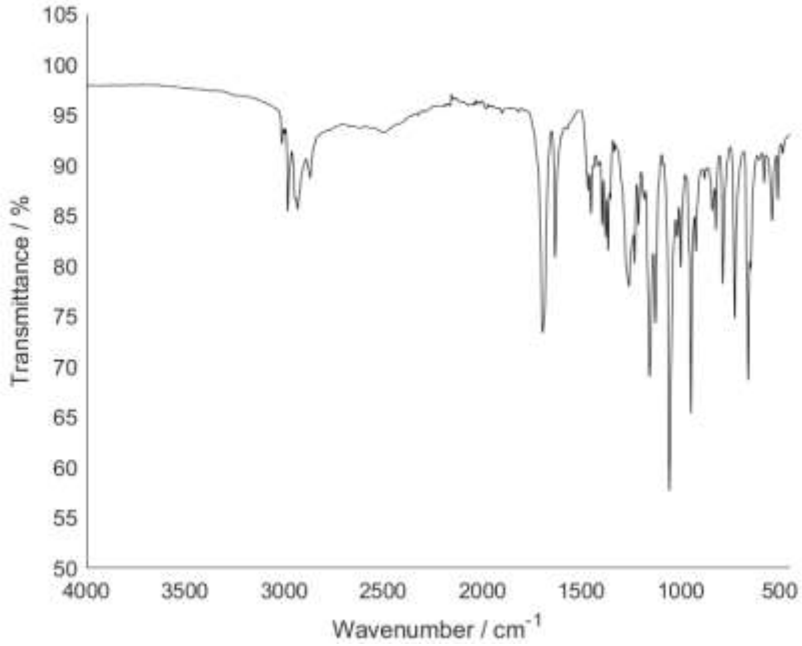

Figure S50: 2-(TEMPOmethyl)acrylic acid IR spectrum.

S9.1.3. CHANT

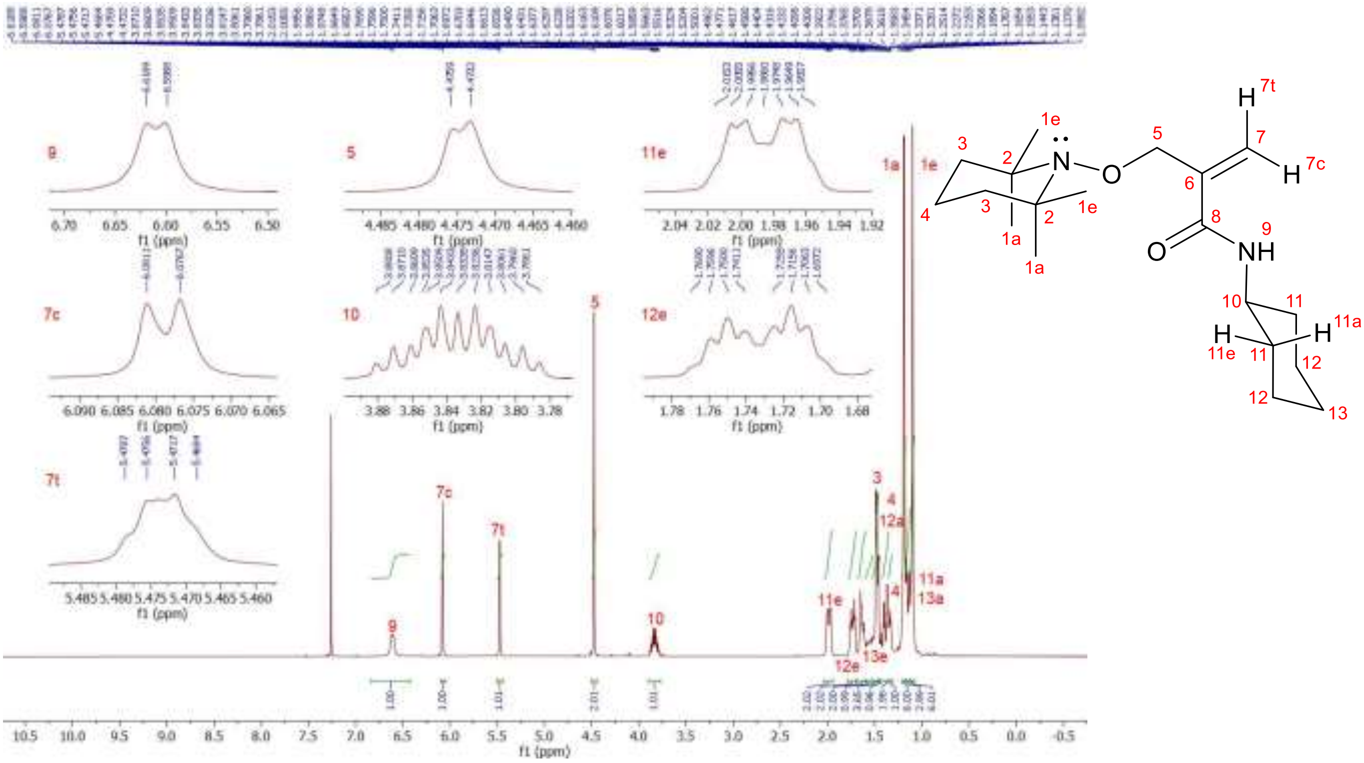

Figure S51: ^{1}H NMR spectrum ($CDCl_3$, 400 MHz, 298 K).

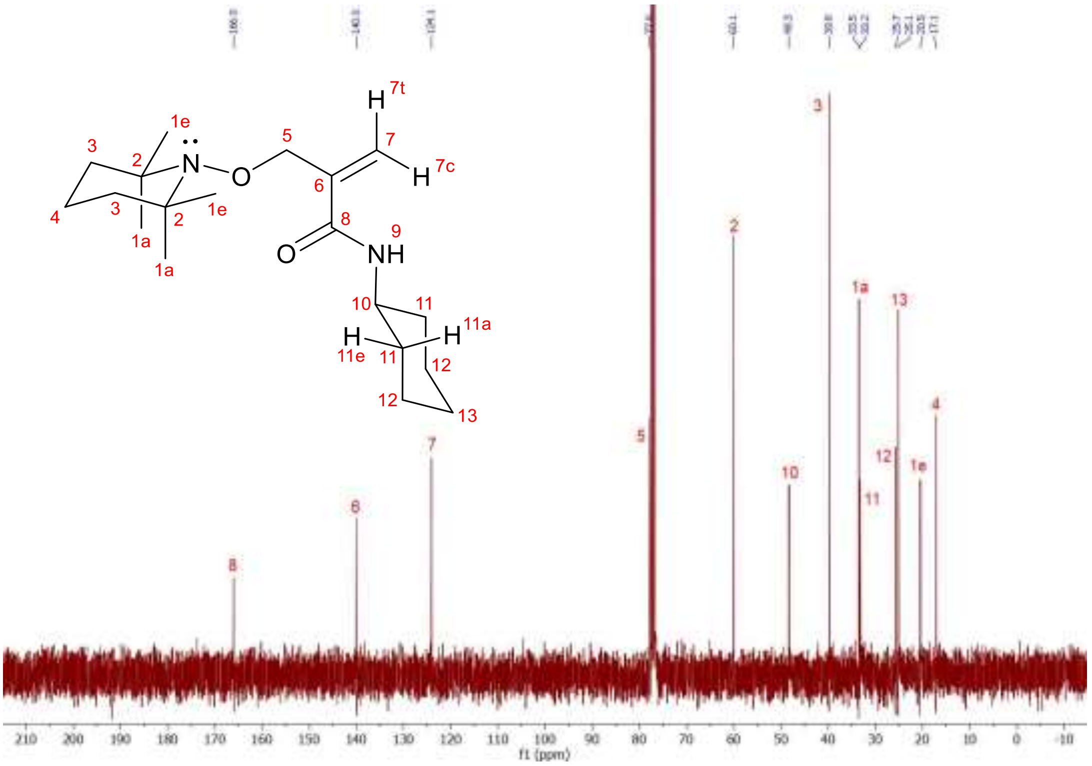

Figure S52: CHANT ^{13}C NMR spectrum ($CDCl_3$, 100 MHz, 298 K).

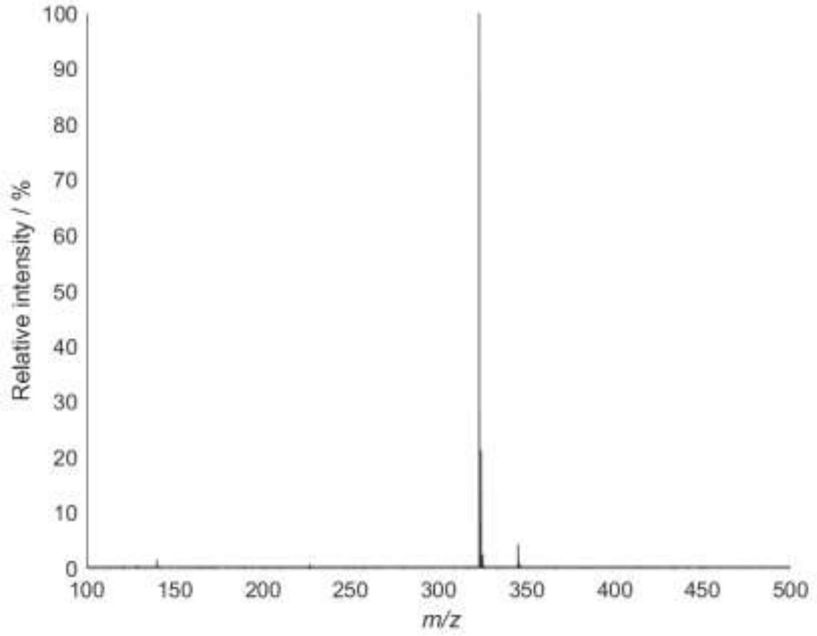

Figure S53: CHANT mass spectrum (Pos ESI).

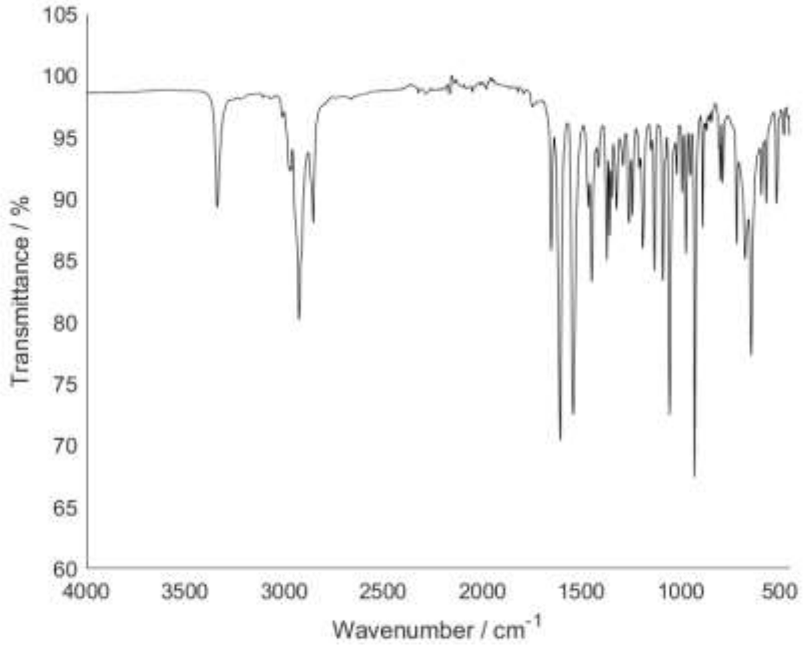

Figure S54: CHANT IR spectrum.

S9.1.4. DEADANT

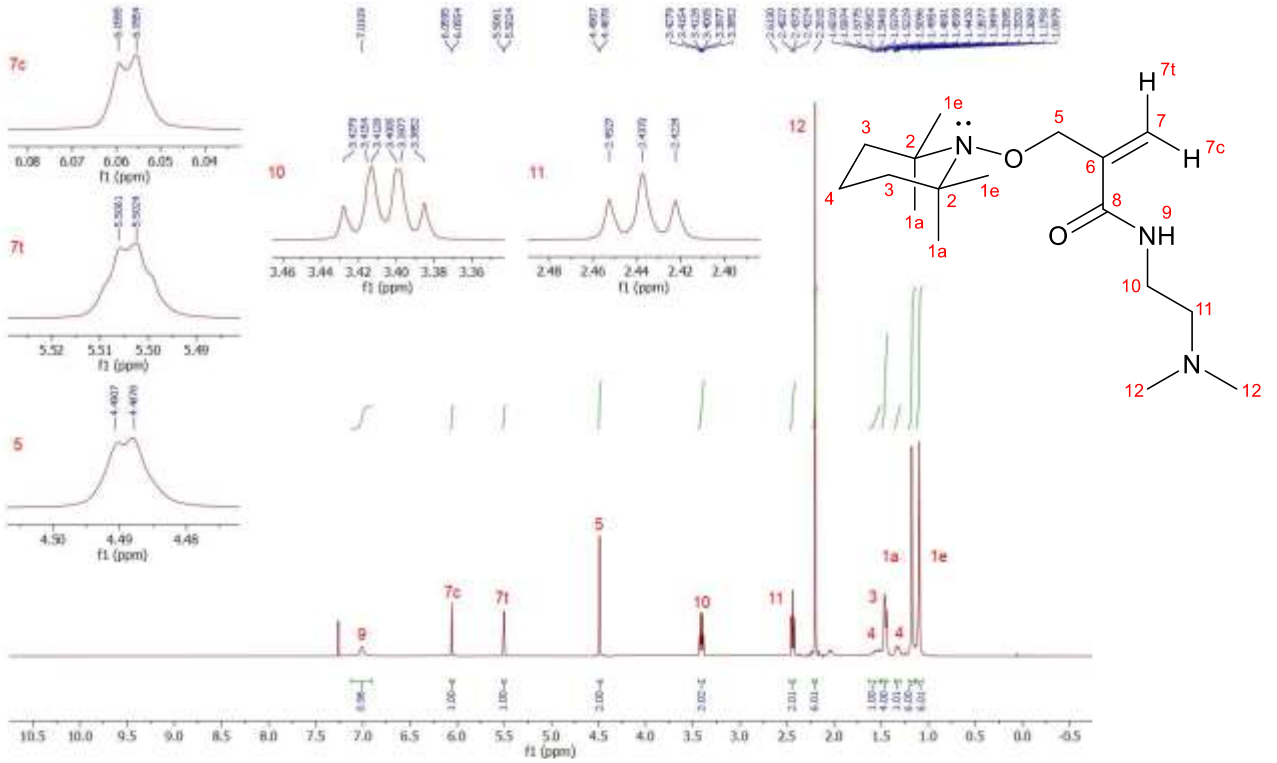

Figure S55:: DEADANT ^{1}H NMR spectrum ($CDCl_3$, 400 MHz, 298 K).

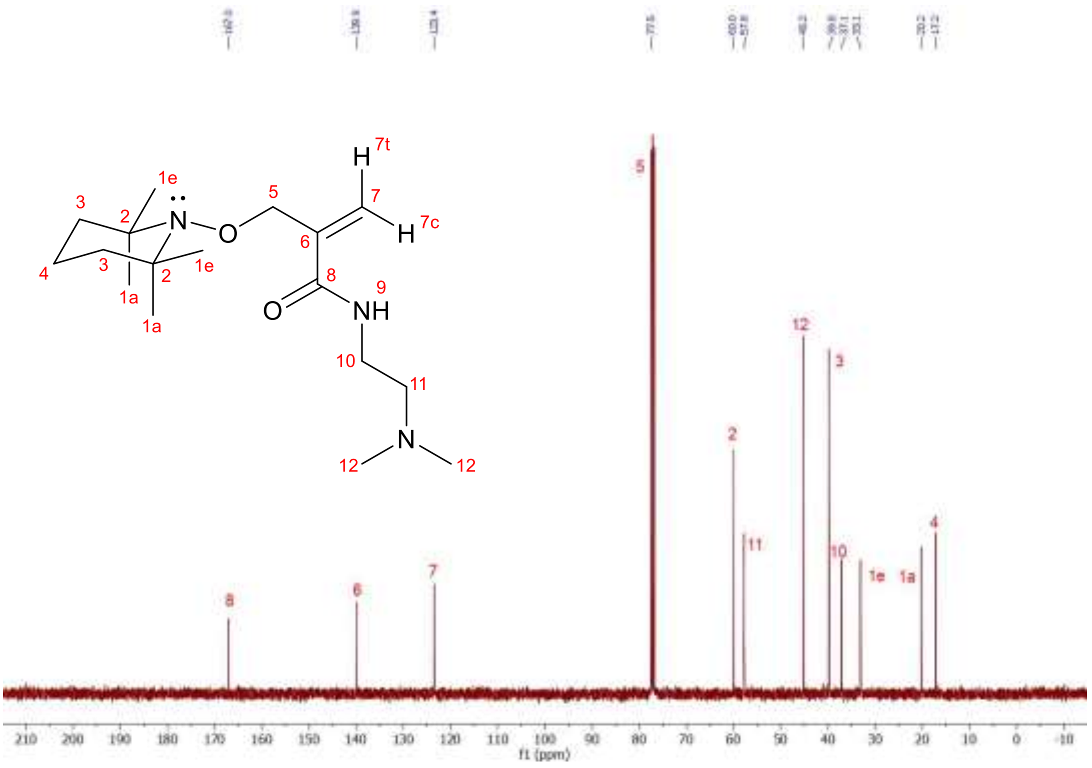

Figure S56: DEADANT ^{13}C NMR spectrum ($CDCl_3$, 100 MHz, 298 K).

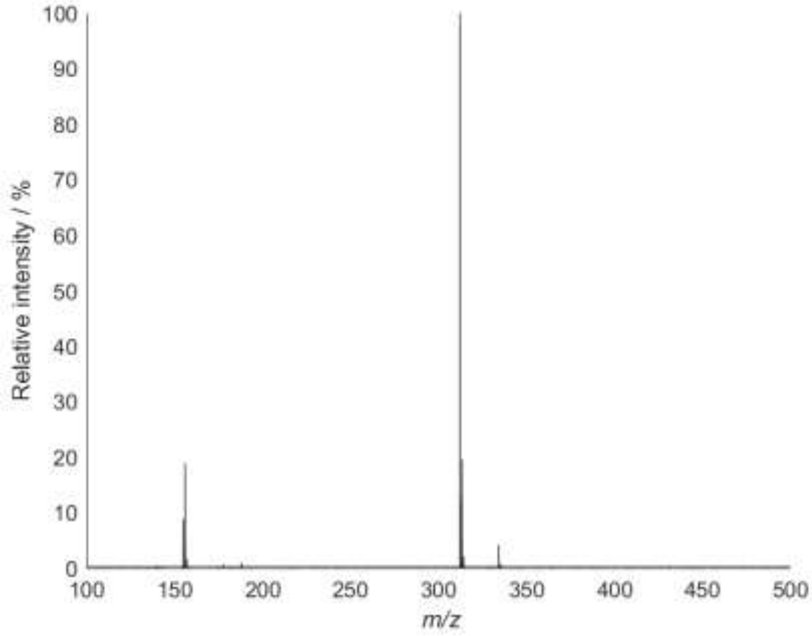

Figure S57: DEADANT mass spectrum (Pos ESI).

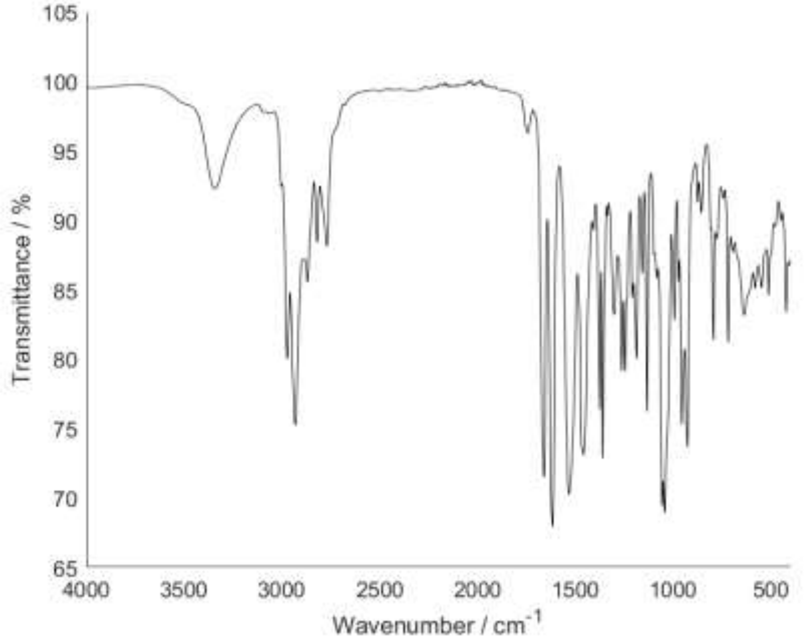

Figure S58: DEADANT IR spectrum.

S9.2. Trapping reactions

S9.2.1. Thiol-ene addition: Isolation of CHANT-trapped PhS• (*N*-cyclohexyl-2-[(phenylsulfanyl)methyl]acrylamide)

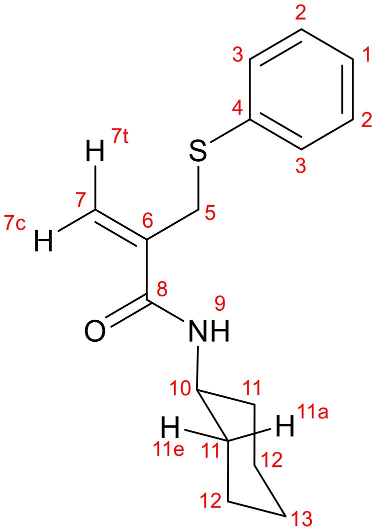

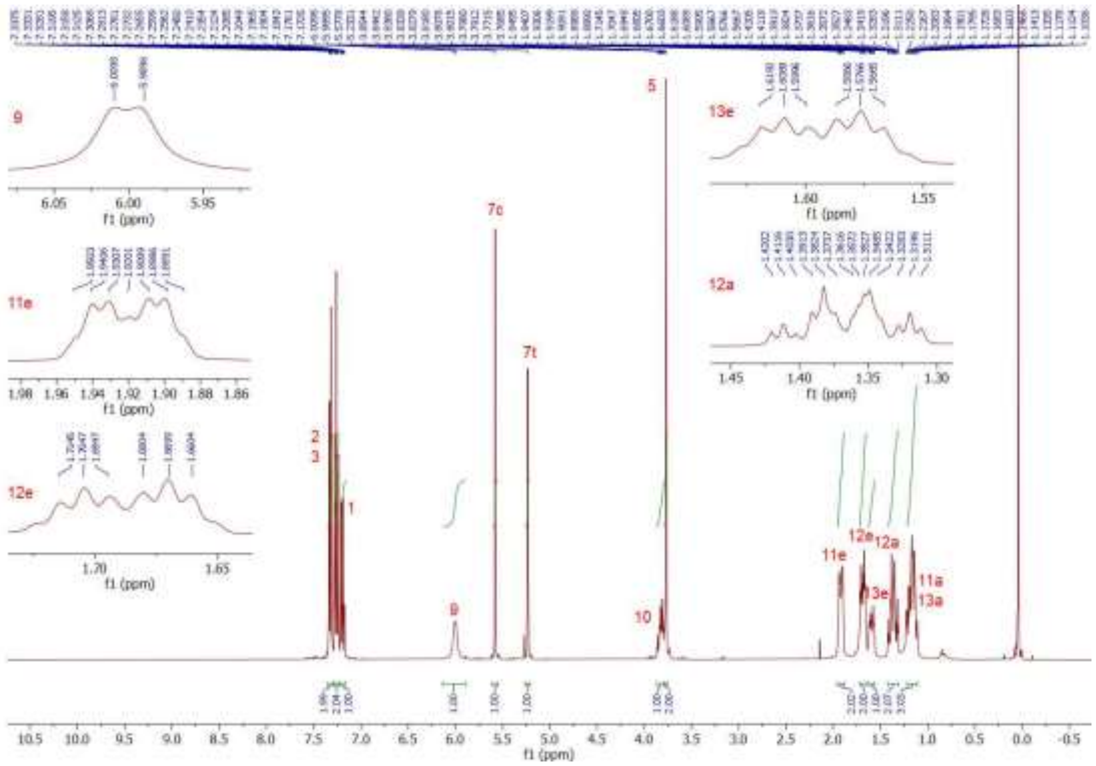

Figure S59:: *N*-Cyclohexyl-2-[(phenylsulfanyl)methyl]acrylamide ^{1}H NMR spectrum ($CDCl_3$, 400 MHz, 298 K).

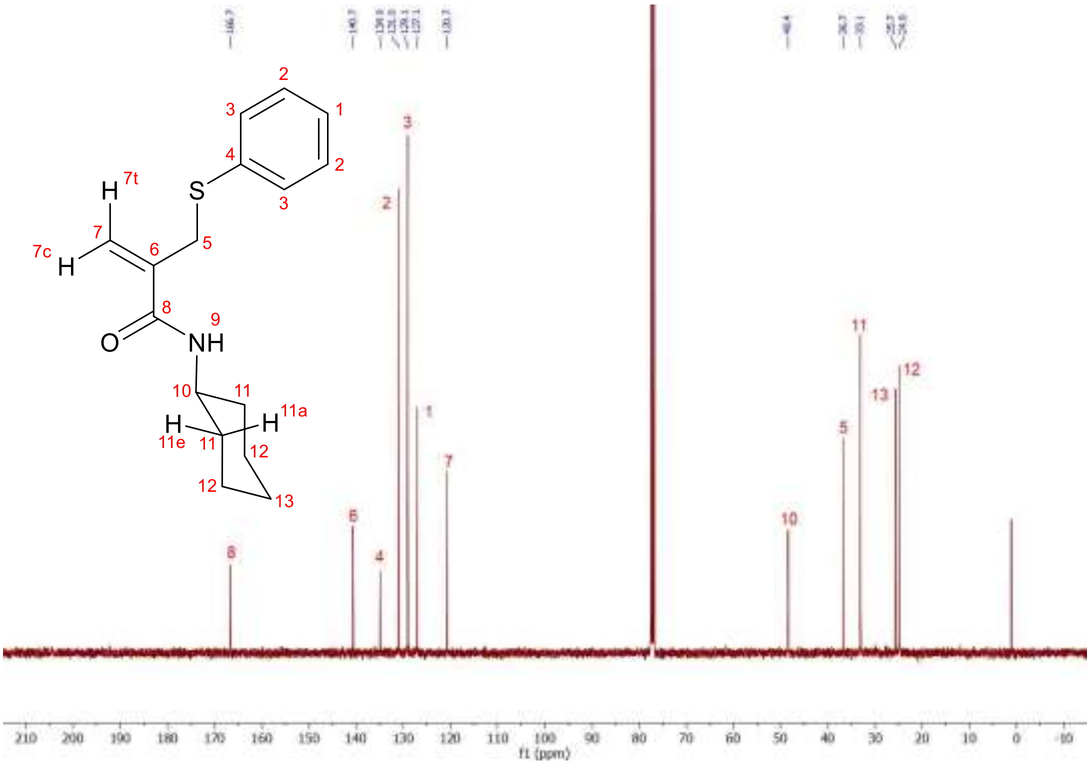

Figure S60: *N*-Cyclohexyl-2-[(phenylsulfanyl)methyl]acrylamide ^{13}C NMR spectrum ($CDCl_3$, 100 MHz, 298 K).

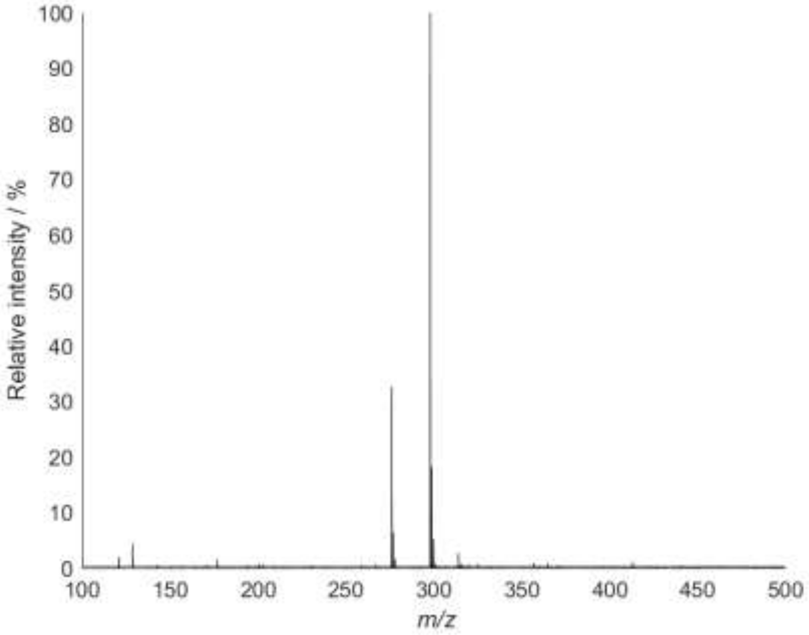

Figure S61: *N*-Cyclohexyl-2-[(phenylsulfanyl)methyl]acrylamide mass spectrum (Pos ESI).

S9.2.2. Hofmann-Löffler-Freytag (HLF) reaction: characterisation of precursor

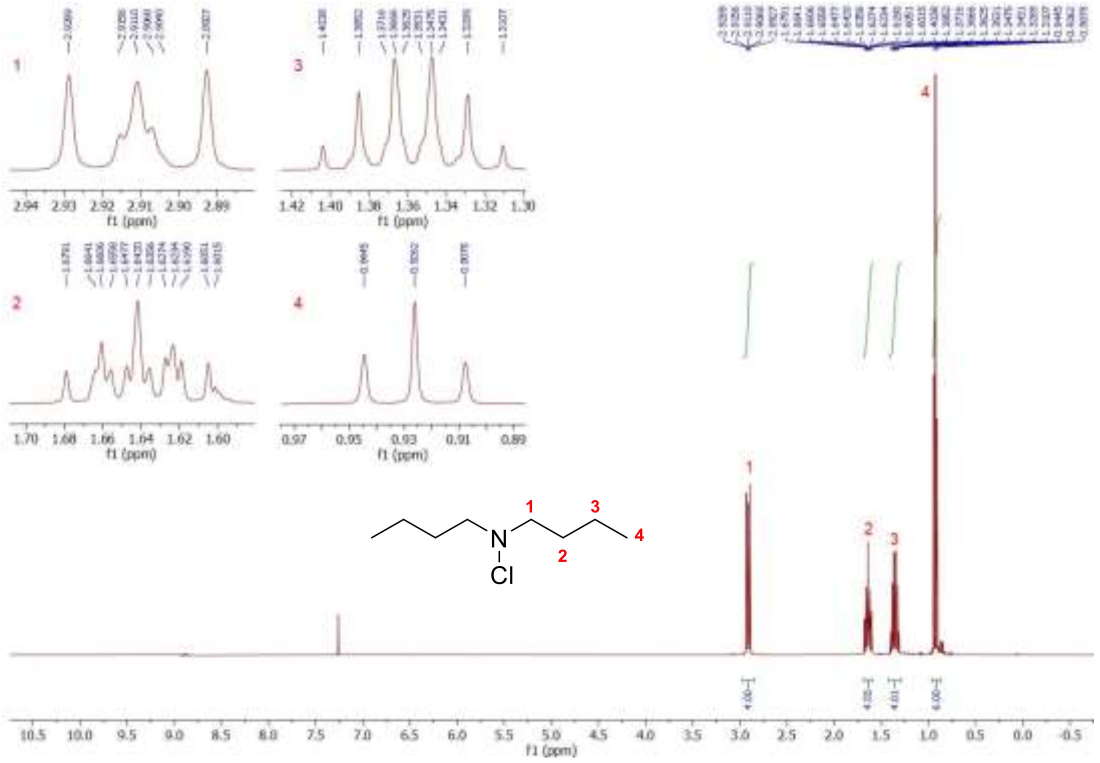

Figure S62: *N*-Chlorodibutylamine ^{1}H NMR spectrum ($CDCl_3$, 400 MHz, 298 K).

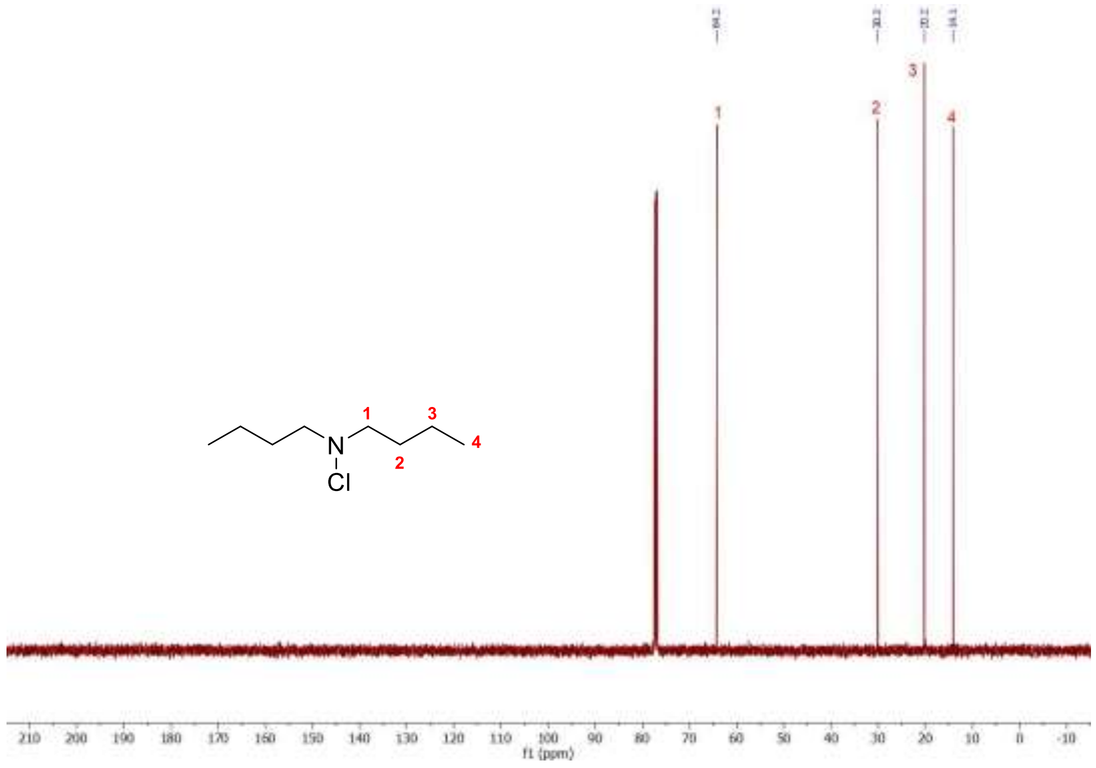

Figure S63: *N*-Chlorodibutylamine ^{13}C NMR spectrum ($CDCl_3$, 100 MHz, 298 K).

S10. Kinetic modelling equations

Table S20: Reactions and their kinetic factors used for gas phase modelling of •OH-initiated *n*-nonane degradation. This model was imported from the MCM and truncated[S17]. Key parameters: residence time = 129 ms. Concentrations / molec. cm^{-3}: [•OH] = $3.4\pm0.5\times10^{11}$; [HO_2•] = $3.4\pm0.5\times10^{11}$; [*n*-nonane] = 1.0×10^{16}.

A	E_a	Reaction[a,b]
1.7E-12	940	OH + O3 ==> HO2
7.7E-12	2100	OH + H2 ==> HO2
1	0	OH + CO ==> HO2
2.9E-12	160	OH + H2O2 ==> HO2
2.03E-16	-693	HO2 + O3 ==> OH
4.8E-11	-250	OH + HO2 ==> H2O + O2
2.2E-13	0	HO2 + HO2 ==> H2O2
2.51E-17	-447	OH + NC9H20 ==> NONO2
2.5899E-13	-1300	NONO2 + HO2 ==> NONOOH
5.00E-14	0	NONO2 + RO2 ==> NON3ONE
1.5E-13	0	NONO2 + RO2 ==> NONO
5E-14	0	NONO2 + RO2 ==> NONOH
3.65E-11	0	OH + NONOOH ==> NON3ONE + OH
2.58E+11	3430	NONO ==> HO3C96O2
1.07E-11	0	OH + NON3ONE ==> C91O2
1.87E-11	0	OH + NONOH ==> NON3ONE + HO2
2.5899E-13	-1300	HO3C96O2 + HO2 ==> HO3C96OOH
5E-14	0	HO3C96O2 + RO2 ==> HO36C9
1.5E-13	0	HO3C96O2 + RO2 ==> HO3C96O
5E-14	0	HO3C96O2 + RO2 ==> HO4CO7C9
2.5899E-13	-1300	C91O2 + HO2 ==> C91OOH
2.5E-13	0	C91O2 + RO2 ==> C91O
4.55E-11	0	OH + HO3C96OOH ==> HO4CO7C9 + OH
5.55E+11	2945	HO3C96O ==> HO4CO7C9 + HO2
2.43E-11	0	OH + HO36C9 ==> HO4CO7C9 + HO2
6.0496E-12	0	OH + HO4CO7C9 ==> C93O2
1.38504E-11	0	OH + HO4CO7C9 ==> CO36C9 + HO2
2.88E-11	0	OH + C5H11CHO ==> C5H11CO3
8.8E-11	0	OH + C91OOH ==> C91O2
1.15E+11	3430	C91O ==> C92O2
1.14E-11	0	OH + CO36C9 ==> C94O2

[a]NC9H20 = *n*-nonane; NONO2 = **SR1**; NONOOH = **SP1.2**; NON3ONE = **SP1.4**; NONO = **SR1.1**; NONOH = **SP1.3**; HO3C96O2 = **SR1.1.1**

S11. References

S1. E. L. Tyson, M. S. Ament, T. P. Yoon, Transition Metal Photoredox Catalysis of Radical Thiol-Ene Reactions. *J. Org. Chem.* **78**, 2046–2050 (2013).

S2. R. Göttlich, Copper(I)-Catalyzed Intramolecular Addition of N-Chloroamines to Double Bonds under Aprotic Conditions. Towards a Stereoselective Catalytic Radical Reaction. *Synthesis (Stuttg).* **11**, 1561–1564 (2000).

S3. S. Pandiancherri, D. W. Lupton, Preparation of 2-azaallyl anions and imines from N-chloroamines and their cycloaddition and allylation. *Tetrahedron Lett.* **52**, 671–674 (2011).

S4. Y. Xie, M. Sun, H. Zhou, Q. Cao, K. Gao, C. Niu, H. Yang, Enantiospecific Total Synthesis of (+)-Tanikolide via a Key [2,3]-Meisenheimer Rearrangement with an Allylic Amine N-Oxide-Directed Epoxidation and a One-Pot Trichloroisocyanuric Acid N-Debenzylation and N-Chlorination. *J. Org. Chem.* **78**, 10251–10263 (2013).

S5. S. L. Titouani, J.-P. Lavergne, P. Viallefont, R. Jacquier, Nouvelles synthèses de l-amino-acids — I: Synthèse stèréospécifique de l-proline, cis(trans) méthyl-3(4) l-prolines. *Tetrahedron*. **36**, 2961–2965 (1980).

S6. L. Onel, A. Brennan, P. W. Seakins, L. Whalley, D. E. Heard, A new method for atmospheric detection of the CH_3O_2 radical. *Atmos. Meas. Tech.* **10**, 3985–4000 (2017).

S7. J. Ortega, C. González, S. Galván, Vapor-Liquid Equilibria for Binary Systems Composed of a Propyl Ester (Ethanoate, Propanoate, Butanoate) + an n-Alkane (C7, C9). *J. Chem. Eng. Data.* **46**, 904–912 (2001).

S8. J. W. Raymonda, W. T. Simpson, Experimental and Theoretical Study of Sigma-Bond Electronic Transitions in Alkanes. *J. Chem. Phys.* **47**, 430–448 (1967).

S9. D. H. R. Barton, R. H. Hesse, M. M. Pechet, L. C. Smith, The Mechanism of the Barton Reaction. *J. Chem. Soc., Perkin Trans. 1*, 1159–1165 (1979).

S10. J. Cason, M. J. Kalm, R. H. Mills, Some Aspects of the Reaction of Silver Carboxylates with Bromine. *J. Org. Chem.* **18**, 1670–1674 (1953).

S11. H. Merten, H. Gilman, Some Long-chained Organosilicon Compounds. *J. Am. Chem. Soc.* **76**, 5798–5799 (1954).

S12. G. J. P. Perry, J. M. Quibell, A. Panigrahi, I. Larrosa, Transition-Metal-Free Decarboxylative Iodination: New Routes for Decarboxylative Oxidative Cross-Couplings. *J. Am. Chem. Soc.* **139**, 11527–11536 (2017).

S13. M. Kimura, M. Tohma, T. Tomita, Metal Ion Catalyzed Oxidation of Steorids. II. Oxidation of Cholestrerol by Fenton's Reagent in Acetic Acid Solution. *Chem. Pharm. Bull.* **20**, 2185–2091 (1972).

S14. J. Woodward, K. W. Lennon, G. Zanin, M. Wagner, M. A. Scott, Interaction between hydrogen peroxide and ferrous sulfate as a basis for glucose determinations. *Biotechnol. Lett.*, **7**, 197–202 (1985).

S15. S.-S. Liang, Y.-L. Shiue, C.-J. Kuo, S.-E. Guo, W.-T. Liao, E.-M. Tsai, Online Monitoring Oxidative Products and Metabolites of Nicotine by Free Radicals Generation with Fenton Reaction in Tandem Mass Spectrometry. *Sci. World J.*, **2013**, 189162 (2013).

S16. J. C. Ianni, Kintecus, Windows Version 6.50 (2018), (available at www.kintecus.com).

S17. Master Chemical Mechanism, MCM v3.3.1 (Jenkin et al., Atmos. Environ., 31, 81, 1997; Saunders et al., Atmos. Chem. Phys., 3, 161, 2003; Jenkin et al., Atmos. Chem. Phys., 15, 11433, 2015, available at http://mcm.york.ac.uk).

S18. J. A. Howard, Absolute Rate Constants for Hydrocarbon Autoxidation. XXII. The Autoxidation of some Vinyl Compounds. *Can. J. Chem.* **50**, 2298–2304 (1972).

S19. J. A. Howard, *Reactions of Organic Peroxyl Radicals in Organic Solvents*, in: *The Chemistry of Free Radicals: Peroxyl Radicals*, Z. B. Alfassi, Ed., Wiley, 1997, pp. 283–334.

S20. J. E. Bennett, G. Brunton, J. R. Lindsay Smith, T. M. F. Salmon, D. J. Waddington, Reactions of alkyl peroxyl radicals in solution. Part 2: A kinetic and product study of self-reaction of 2-propylperoxyl radicals between 253 and 323 K, *J. Chem. Soc., Faraday Trans. 1.* **83**, 2433–2447 (1987).

S21. P. Neta, R. E. Huie, A. B. Ross, Rate Constants for Reactions of Peroxyl Radicals in Fluid Solutions, *J. Phys. Chern. Ref. Data*. **19**, 413–513 (1990).

S22. A. R. Costello, J. R. Lindsay Smith, M. S. Stark, D. J. Waddington, Kinetic study of the reactions of tert-butyl radicals in the liquid phase in the presence and absence of oxygen, J. Chem. Soc., Faraday Trans. **92**, 3497–3507 (1996).

S23. P. C. Wong, D. Griller, J. C. Scaiano, A Kinetic Study of the Reactions of tert-Butoxyl with Alkenes: Hydrogen Abstraction vs. Addition. *J. Am. Chem. Soc*. **104**, 5106–5108 (1982).

S24. J. H. Horner, S.-Y. Choi, M. Newcomb, Laser flash photolysis studies of alkoxyl radical kinetics using 4-nitrobenzenesulfenate esters as radical precursors. *Org. Lett.* **2**, 3369–3372 (2000).

S25. S. Goldstein, A. Samuni, Kinetics and mechanism of peroxyl radical reactions with nitroxides, *J. Phys. Chem. A.* 111, 1066–1072 (2007).

S26. B. H. Northrop, R. N. Coffey, Thiol–Ene Click Chemistry: Computational and Kinetic Analysis of the Influence of Alkene Functionality. *J. Am. Chem. Soc.* **134**, 13804–13817 (2012).

S27. G. Povie, A.-T. Tran, D. Bonnaffé, J. Habegger, Z. Hu, C. Le Narvor, P. Renaud, Repairing the Thiol-Ene Coupling Reaction. *Angew. Chem. Int. Ed.* **53**, 3894–3898 (2014).

S28. U. Biermann, J. O. Metzger, Regioselectivity of Radical Addition of Thiols to 1-Alkenes, *Eur. J. Org. Chem.* **2018**, 730–734 (2018).

S29. C. Chatgilialoglu, A. Altieri, H. Fischer, The Kinetics of Thiyl Radical-Induced Reactions of Monounsaturated Fatty Acid Esters. *J. Am. Chem. Soc.* **124**, 12816–12823 (2002).

S30. F. Dénès, M. Pichowicz, G. Povie, P. Renaud, *Thiyl Radicals in Organic Synthesis.* **114**, 2587–2693 (2014).

S31. M. Vicevic, K. Novakovic, K. Boodhoo, Free-Radical Polymerization of Styrene: Kinetic Study in a Spinning Disc Reactor (SDR), *Front. Chem. Eng.* 3: 661498 (2021).

S32. E. J. Corey, W. R. Hertler, A Study of the Formation of Haloamines and Cyclic Amines by the Free Radical Chain Decomposition of N-Haloammonium Ions (Hofmann-Löffler Reaction). *J. Am. Chem. Soc*. **82**, 1657–1668 (1960).

S33. L. Vereecken, J. F. Müller, J. Peeters, Low-volatility poly-oxygenates in the OH-initiated atmospheric oxidation of α-pinene: impact of non-traditional peroxyl radical chemistry. *Phys. Chem. Chem. Phys.* **9**, 5241–5248 (2007).

S34. L. Vereecken, J. Peeters, Non-traditional (Per)oxy Ring-Closure Paths in the Atmospheric Oxidation of Isoprene and Monoterpenes. *J. Phys. Chem. A*. **108**, 5197–5204 (2004).

S35. T. Berndt, Peroxy Radical Processes and Product Formation in the OH Radical-Initiated Oxidation of α-Pinene for Near-Atmospheric Conditions. *J. Phys. Chem. A*. **125**, 9151–9160 (2021).

S36. M. E. Jenkin, R. Valorso, B. Aumont, A. R. Rickard, T. J. Wallington, Estimation of rate coefficients and branching ratios for gas-phase reactions of OH with aliphatic organic compounds for use in automated mechanism construction. *Atmos. Chem. Phys*. **18**, 9297–9328 (2018).

S37. D. R. Barton, J. M. Beaton, L. E. Geller, M. M. Pechet, A New Photochemical Reaction. *J. Am. Chem. Soc*. **83**, 4076–4083 (1961).

S38. A. Borodin, Über Bromvaleriansäure und Brombuttersäure. *Justus Liebigs Ann. Chem*. **119**, 121–123 (1861).

S39. H. Hunsdiecker, C. Hunsdiecker, Über den Abbau der Salze aliphatischer Säuren durch Brom. *Chem. Ber*. **75**, 291–297 (1942).

S40. R. G. Johnson, R. K. Ingham, The degradation of carboxylic acid salts by means of halogen: The Hunsdiecker reaction. *Chem. Rev*. **56**, 219–269 (1956).

S41. W. K. Plucknett, H. L. Richards, Stability Constants for Iodine-Aromatic Complexes in Cyclohexane. *J. Chem. Eng. Data* **8**, 239–240 (1963).

S42. Y. P. A. de Oliveira, L. C. Pontes-de-Carvalho, R. D. Couto, A. A. Noronha-Dutra, Oxidative stress in sepsis. Possible production of free radicals through an erythrocyte-mediated positive feedback mechanism. *Brazilian J. Infect. Dis*. **21**, 19–26 (2017).

S43. J. Cadet, T. Delatour, T. Douki, D. Gasparutto, J. P. Pouget, J. L. Ravanat, S. Sauvaigo, Hydroxyl radicals and DNA base damage. *Mutat. Res*. **424**, 9–21 (1999).
